# A synthetic approach towards drug modification: 2-hydroxy-1-naphthaldehyde based imine-zwitterion preparation, single-crystal study, Hirshfeld surface analysis, and computational investigation†

**DOI:** 10.1039/d3ra08727a

**Published:** 2024-02-21

**Authors:** Abida Naseem Malik, Akbar Ali, Muhammad Ashfaq, Muhammad Nawaz Tahir, Mohammad Mahtab Alam, Mohamed S. Mostafa, Aleksey Kuznetsov

**Affiliations:** a Department of Physics, University of Sargodha Sargodha 40100 Pakistan muhammadashfaq1400@gmail.com; b Department of Chemistry, Government College University Faisalabad 38000-Faisalabad Pakistan akbarchm@gmail.com akbarali@gcuf.edu.pk; c Department of Basic Medical Sciences, College of Applied Medical Science, King Khalid University Abha 61421 Saudi Arabia; d Department of Physical Sciences, College of Science, Jazan University P.O. Box 114 Jazan 45142 Saudi Arabia; e Departamento de Química, Campus Santiago Vitacura, Universidad Tecnica Federico Santa María Av. Santa María 6400 Vitacura 7660251 Chile aleksey.kuznetsov@usm.cl

## Abstract

The current work is about the modification of primary amine functionalized drugs, pyrimethamine and 4-amino-*N*-(2,3-dihydrothiazol-2-yl)benzenesulfonamide, *via* condensation reaction with 2-hydroxy-1-naphthaldehyde to produce new organic zwitterionic compounds (*E*)-1-(((4-(*N*-(2,3-dihydrothiazol-2-yl)sulfamoyl)phenyl)iminio)methyl)naphthalen-2-olate (DSPIN) and (*E*)-1-(((4-amino-5-(4-chlorophenyl)-6-ethylpyrimidin-2-yl)iminio)methyl)naphthalen-2-olate (ACPIN) in methanol as a solvent. The crystal structures of both compounds were confirmed to be imine-based zwitterionic products *via* single-crystal X-ray diffraction (SC-XRD) analysis which indicated that the stabilization of both crystalline compounds is achieved *via* various noncovalent interactions. The supramolecular assembly in terms of noncovalent interactions was explored by the Hirshfeld surface analysis. Void analysis was carried out to predict the crystal mechanical response. Compound geometries calculated in the DFT (Density Functional Theory) study showed reasonably good agreement with the experimentally determined structural parameters. Frontier molecular orbital (FMO) analysis showed that the DSPIN HOMO/LUMO gap is by 0.15 eV smaller than the ACPIN HOMO/LUMO gap due to some destabilization of the DSPIN HOMO and some stabilization of its LUMO. The results of the charge analysis implied formation of intramolecular hydrogen bonds and suggested formation of intermolecular hydrogen bonding and dipole–dipole and dispersion interactions.

## Abstract 2

This work is about the modification of pyrimethamine and 4-amino-*N*-(2,3-dihydrothiazol-2-yl)benzenesulfonamide *via* condensation reaction with 2-hydroxy-1-naphthaldehyde to produce new organic zwitterionic compounds DSPIN and ACPIN in methanol.

## Graphical abstract

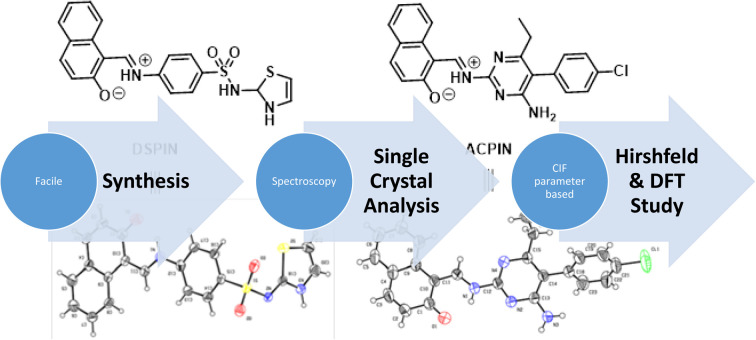

## 1. Introduction

Diseases like influenza, typhoid, typhus, and malaria are conventionally considered as fatal sicknesses due to the unapproachability of operative medications.^1^ Pyrimidine-based chemical building blocks have been effectively used as medications for malaria.^2^ The physicians no more prescribe pyrimethamine as a medication for malaria because of the drug resistance phenomenon.^3^ As pyrimethamine alone has lost its efficacy, its combinations with various chemical building blocks like sulfadoxine-pyrimethamine, sulfamethoxazole-trimethoprim, and sulfalene-pyrimethamine have been used as new antimalarial drugs but they also lost their effectiveness against malaria as time has passed.^4^ These drugs can further be modified for the sake of new medicinal applications. Previously, we have reported the modification of pyrimethamine *via* co-crystallization and the structural and electronic features of the modified compounds were studied by computational methods.^5^

Another important chemical architecture from the point of view of the synthesis and drug development is represented by the sulfonamide derivatives. This class of compounds has presented diverse pharmacological activities including anti-carbonic anhydrase,^6^ antibacterial,^7^ hypoglycaemic,^8^ diuretic,^9^ antithyroid,^10^ and antitumor activity *in vitro* and/or *in vivo*.^11^ The modification of these two pharmacologically significant compound classes is important for the syntheses of diversely functionalized chemical building blocks that have medicinal applications as well as interesting optoelectronic properties. For studies of structures, electronic, optical, and reactivity properties of various compounds, the density functional theory (DFT) has been developed as a very promising tool based on computer-based calculations. There are several features of organic compounds that can be investigated *via* DFT studies, namely, energies of the frontier molecular orbitals, HOMO (the highest occupied molecular orbital) and LUMO (the lowest unoccupied molecular orbital), HOMO–LUMO and optical gaps, noncovalent interactions involving weak attractive forces, and non-linear optical (NLO) properties.^12,13^ Our collaborative research group has been working on the syntheses and theoretical exploration *via* DFT analysis of different classes of compounds with organic origin such as β-hydroxycarbonyl compounds and chalcones,^14^ hydrazones,^15,16^ piperidone derivatives,^17^ peptoids,^18^ phosphonates,^19^ functionalized esters,^20^ monocarbonyl curcuminoids,^21,22^ functionalized pyrimidines,^23,24^ functionalized indoles,^25^ unsymmetrical acyl thioureas,^26^ as well as organic salt systems.^27,28^

In the current study, the primary amine functionalized drugs, that is, pyrimethamine and 4-amino-*N*-(2,3-dihydrothiazol-2-yl)benzenesulfonamide, have been modified *via* the condensation reaction with 2-hydroxy-1-naphthaldehyde which is also an important chemical unit known as a versatile fluorophore for naked eye and fluorometric detections of different anions, cations, and neutral molecules. The final products, namely, (*E*)-1-(((4-(*N*-(2,3-dihydrothiazol-2-yl)sulfamoyl)-phenyl)iminio)methyl)naphthalen-2-olate (DSPIN) and (*E*)-1-(((4-amino-5-(4-chlorophenyl)-6-ethylpyrimidin-2-yl)iminio)methyl)naphthalen-2-olate (ACPIN), have been obtained in crystalline imine-zwitterion forms. Due to their crystalline nature, the products have been studied by the SC-XRD approach, and their structural and electronic features have further been investigated using the Hirshfeld surface analysis and DFT calculations.

## 2. Experimental part

### 2.1. Chemicals and instrumentation

Solvents and chemicals of the highest possible quality were used without any further purification. Single-crystal analysis was performed at temperature 296 K using Bruckner made diffractometer (Kappa APEX II CCD), equipped with a graphite monochromator. The XRD data collection was performed using the fine focus of Mo *K*_α_ X-rays and APEX2 software.^29^ The data reduction was accomplished using SAINT-27 software. Structural parameters (bond distances, hydrogen bond characteristics, torsion angles, and bond angles) were obtained using PLATON software.^30^ SHELXL-97 program was used for the structure solution and direct method was employed for the structure refinement.^31^ ORTEP-3 software^32^ was used for thermal ellipsoid diagram creation while for cyclic interaction diagram creation Mercury 4.0 (ref. 33) was used.

### 2.2. The preparation procedure

**Scheme 1:**
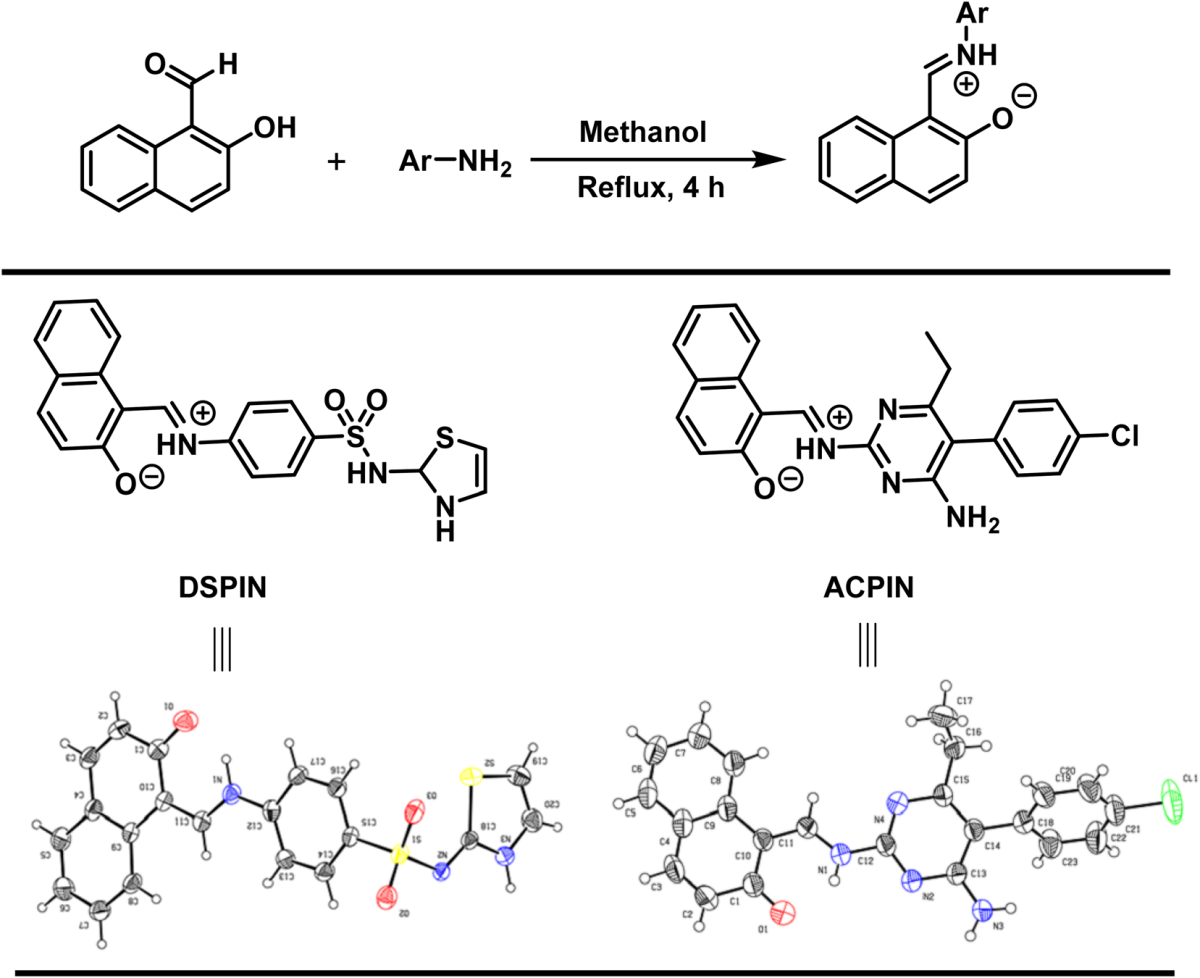
Synthetic scheme for the preparation of (*E*)-1-(((4-(*N*-(2,3-dihydrothiazol-2-yl)sulfamoyl)phenyl)iminio)methyl)naphthalen-2-olate (DSPIN) and (*E*)-1-(((4-amino-5-(4-chlorophenyl)-6-ethylpyrimidin-2-yl)iminio)methyl)naphthalen-2-olate (ACPIN).

Both crystalline compounds were prepared by the condensation reaction. Accordingly, 1.2 mmol each of the 2-hydroxy-1-naphthaldehyde and substituted aromatic primary amines were placed in a 50 mL round bottom flask containing 15 mL of methanol as a solvent. The mixture was stirred under reflux conditions for 4 hours. Once the reaction was completed (monitored by thin layer chromatography), the reaction mixture was cooled to room temperature and was left for 24 hours to obtain crystals of the title organic zwitterion compounds DSPIN and ACPIN (Scheme 1).

### 2.3. Computational details

DFT studies were performed with the Gaussian 16 software.^34^ Using as the starting geometries the structures from the SC-XRD analysis, we optimized the DSPIN and ACPIN molecules without any symmetry constraints and then performed frequency calculations to verify that the optimized structures are true energy minima. All calculations were performed with the hybrid density functional B3LYP^35^ with the triple-zeta split-valence polarized basis set 6-311+G(d,p)^36,37^ (with two sets of polarization functions, on hydrogens and on heavier atoms, and one set of diffuse functions). This approach is furthermore referred to as B3LYP/6-311+G(d,p). We did the computational studies and all analyses listed below with the B3LYP/6-311+G(d,p) approach and with the implicit effects from ethanol (dielectric constant *ε* = 24.852) taken into account, employing the self-reliable IEF-PCM approach^38^ with the UFF default model as implemented in the Gaussian 16 software, with the electrostatic scaling factor *α* = 1.0. Below we compare the calculated structural parameters, Natural Population Analysis (NPA) charges, Natural Bond Orbitals (NBO) orbital interactions,^39^ and frontier molecular orbitals (FMOs) for both compounds. We used the values of the energies of HOMO and LUMO to compute the global reactivity parameters (GRP)^40–42^ (see eqn (1)–(6)). eqn (1) and (2) were used to calculate the values of the ionization potential (IP) and electron affinity (EA):

1 IP = −*E*_HOMO_

2 EA = −*E*_LUMO_

For global hardness *η* and electronegativity *X* values we used eqn (3) and (4):3
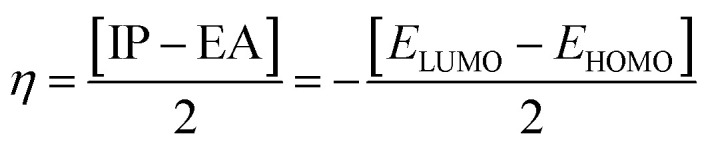
4
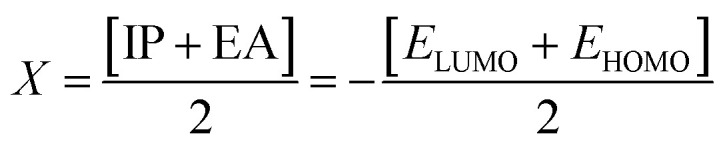


Global electrophilicity *ω* value was calculated by eqn (5):5
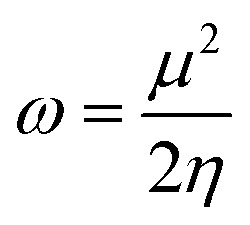
where 
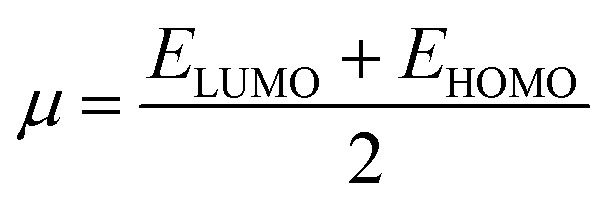
 is the chemical potential of the system.

Finally, the global softness *σ* value was computed with the eqn (6):6
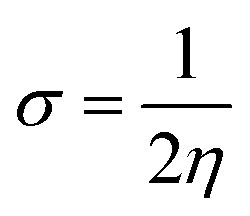


Open GL version of Molden 5.8.2 visualization program was used for the visualization of the structures of the title compounds.^43^ Avogadro, version 1.1.1, was used to visualize the molecular orbitals and molecular electrostatic potential (MEP) maps.^44,45^

## 3. Results and discussions

### 3.1. Single-crystal XRD study of DSPIN and ACPIN

**Fig. 1:**
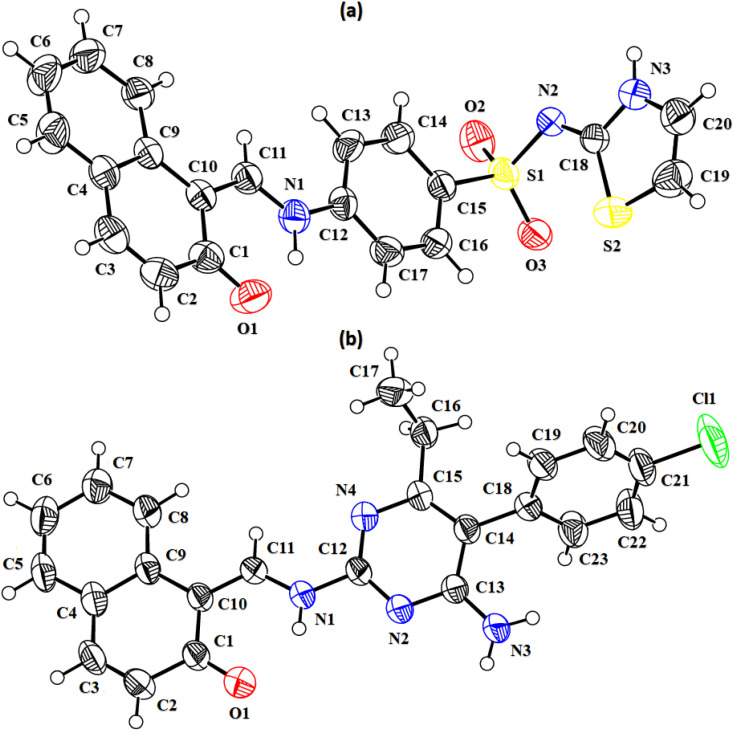
ORTEP diagrams of the compound DSPIN (a) and ACPIN (b) drawn at a probability level of 50%. H-atoms shown by tiny circles of arbitrary radii.

**Fig. 2:**
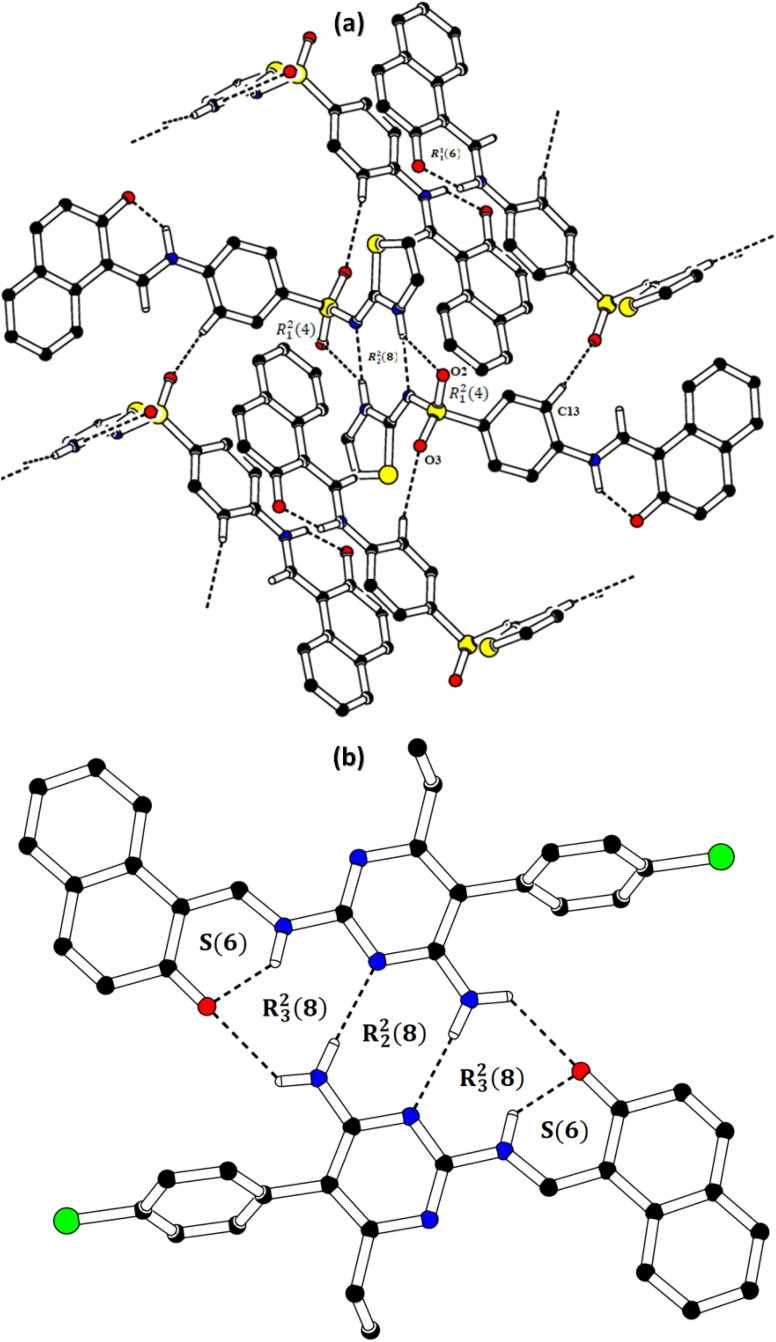
Packing diagrams of DSPIN (a) and ACPIN (b). Only selected H-atoms shown for clarity.

**Fig. 3:**
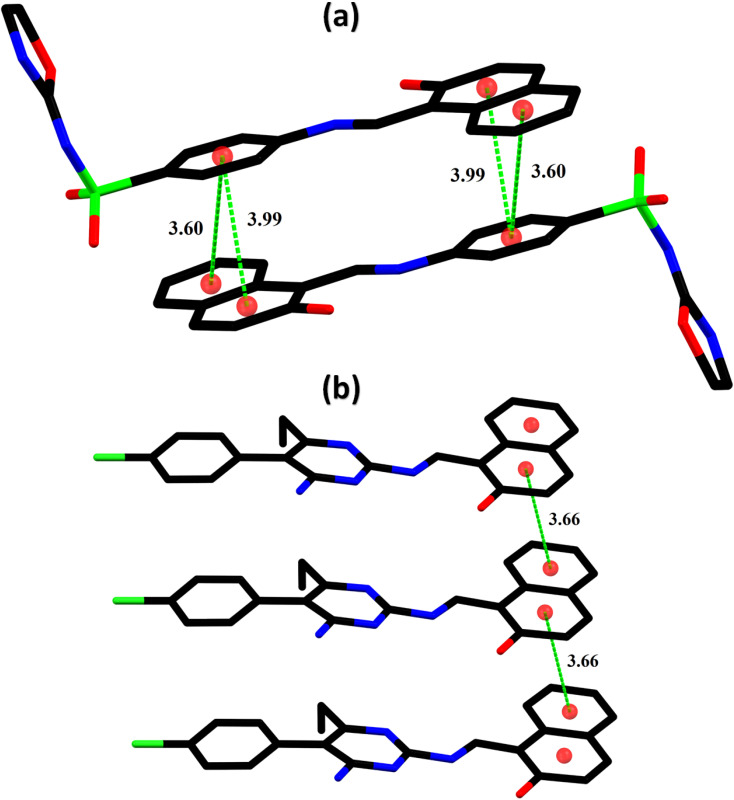
Graphical representation of the off-set π⋯π stacking interactions in DSPIN (a) and ACPIN (b). H-atoms not shown for clarity. Distances measured in Å.

In the zwitterion crystal structure of 1-((*Z*)-((4-((*E*)-*N*-(thiazol-2(3*H*)-ylidene)-sulfamoyl)phenyl)iminio)methyl)naphthalen-2-olate (Fig. 1a and Table 1), the 1-methylnaphthalen-2-olate group A (C1–C11/O1), 4-aminobenzenethiol group B (C12–C17/N1/S1), and thiazol-2(3*H*)-imine group C (C18–C20/N2/N3/S2) are roughly planar with root mean square (r.m.s.) deviations of 0.0227, 0.0042, and 0.0078 Å, respectively. The central group B is oriented at the dihedral angle of 3.63(7)° and 77.2(7)° with respect to the groups A and C, respectively. The dihedral angles showed that groups A and B are almost parallel to each other. The geometry around sulphur atom (S1) is a distorted tetrahedron as the value of the structural parameter (τ_4_) is 0.92. The molecular configuration is stabilized by intramolecular N–H⋯O bonding to form S(6) H-bonded loop. The molecules are interlinked in the form of a dimer through N–H⋯N bonding to form *R*_2_^2^(8) loops^46^ where the N-atom attached to the sulfonyl group acts as H-bond acceptor and NH of the five-membered ring acts as H-bond donor (Fig. 2a and Table 3). The same NH group interacts with one of the O-atoms of the sulfonyl group and completes two bifurcated *R*_1_^2^(4) loops. The other O-atom of the sulfonyl group acts as H-bond acceptor for CH of the phenyl ring. As the result of N–H⋯N, N–H⋯O, and C–H⋯O bonding interactions, one-dimensional network of molecules is formed with the base vector [0 0 1]. The crystal packing is further stabilized by off-set π⋯π stacking interactions between aromatic rings of the molecules that are related by inversion symmetry (Fig. 3a). The inter-centroid separation ranges from 3.60 to 3.99 Å and ring off-set ranges from 0.580 to 2.098 Å. Cambridge structural database search provides two hints that have close similarity with the crystal structure of DSPIN with reference codes WUFFUA^47^ and UTANAG.^48^ The bond lengths and bond angles of DSPIN (Table 2) are consistent with the corresponding structural parameters in the related structures. The crystal structure with the reference code WUFFUA is crystallized in the monoclinic system with the space group *P*1̄ and is a polymorph of DSPIN whereas UTANAG is the hydrated form of DSPIN. Just like WUFFUA and UTANAG, the molecule of DSPIN is non-planar and has intramolecular N–H⋯O bonding. The crystal packing of DSPIN is different from the crystal packing of its polymorph. In WUFFUA, C–H⋯π interactions are present which are absent in DSPIN. Similarly, C–H⋯O bonding is absent in WUFFUA but present in DSPIN. The crystal packing of DSPIN has stronger π⋯π stacking interactions as compared to WUFFUA. The presence of water molecules makes the crystal packing of UTANAG entirely different from the crystal packing of DSPIN and WUFFUA. In UTANAG, both H-atoms of the water molecule act as H-bond donors for the O-atom of the sulfonyl group.

**Table:** SC-XRD experimental details of the compounds DSPIN and ACPIN

Crystal data	DSPIN	ACPIN
CCDC	2 263 537	2 263 538
Chemical formula	C_20_H_15_N_3_O_3_S_2_	C_27_H_21_ClN_4_O
*M*_r_	409.47	402.87
Crystal system, space group	Monoclinic, *P*2_1_/*c*	Triclinic, *P*1̄
Temperature (K)	296	296
*a*, *b*, *c* (Å)	7.8389 (8), 13.8151 (16), 17.198 (2)	4.5436 (6), 12.5255 (16), 17.673 (2)
*α*, *β*, *γ*°	94.032 (4)	88.308 (8), 89.195 (8), 82.611 (9)
*V* (Å^3^)	1857.9 (4)	996.9 (2)
*Z*	4	2
Density (calculated) g cm^−3^	1.464	1.324
*F*(000)	848	420
Radiation type	Mo *K*α	Mo *K*α
Wavelength (*λ*)	0.71073 Å	0.71073 Å
*μ* (mm^−1^)	0.31	0.21
Crystal size (mm)	0.32 × 0.28 × 0.23	0.40 × 0.24 × 0.20

**Data collection**		
Diffractometer	Bruker APEXII CCD diffractometer	Bruker APEXII CCD diffractometer
Absorption correction	Multi-scan (SADABS; Bruker, 2007)	Multi-scan (SADABS; Bruker, 2007)
No. of measured, independent, and observed [*I* > 2σ(*I*)] reflections	15 333, 4040, 2547	4351, 4351, 2397
*R*_int_	0.0404	0.0454
Theta range for data collection (°)	2.374 to 27.000	
Index ranges	−9 ≤ *h* ≤ 9, −10 ≤ *k* ≤ 10, −14 ≤ *l* ≤ 14	−5 ≤ *h* ≤ 5, −16 ≤ *k* ≤ 16, −1 ≤ *l* ≤ 22
(Sin *θ*/*λ*)_max_ (Å^−1^)	0.639	0.649

**Data refinement**		
*R*[*F*^2^ > 2*σ*(*F*^2^)], *wR*(*F*^2^), *S*	0.044, 0.109, 1.02	0.076, 0.236, 1.14
No. of reflections	4040	4351
No. of parameters	259	264
No. of restraints		
H-atom treatment	H atoms treated by a mixture of independent and constrained refinement	H-atom parameters constrained
Δ*ρ*_max_, Δ*ρ*_min_ (e Å^−3^)	0.26, −0.27	0.32, −0.28

Important bond lengths (Å) and bond angles (°) for DSPIN and ACPIN, X-ray//DFT

**Table:** 

Selected bond lengths (Å) in DSPIN		Selected bond lengths (Å) in ACPIN	
S1–O2	1.4315 (17)//1.470	Cl1–C21	1.740 (4)//1.764
S1–O3	1.4344 (17)//1.479	O1–C1	1.270 (5)//1.259
S1–N2	1.6056 (18)//1.642	N1–C11	1.320 (5)//1.339
O1–C1	1.269 (3)//1.263	N1–C12	1.405 (5)//1.402
N1–C11	1.325 (3)//1.337	N2–C12	1.323 (5)//1.331
N3–C20	1.370 (3)//1.387	N2–C13	1.343 (5)//1.342
S1–C15	1.762 (2)//1.792	N3–C13	1.339 (5)//1.356
S2–C19	1.719 (3)//1.760	N4–C12	1.315 (5)//1.324
S2–C18	1.724 (2)//1.777	N4–C15	1.353 (5)//1.352
N1–C12	1.402 (3)//1.402	—	—
N3–C18	1.338 (3)//1.358	—	—
N2–C18	1.325 (3)//1.312	—	—

**Table:** 

Selected bond angles (°) in DSPIN		Selected bond angles (°) in ACPIN	
O2–S1–O3	118.78 (11)//117.89	N4–C12–N1	118.6 (3)//118.0
O2–S1–N2	104.17 (10)//107.24	N2–C12–N1	112.8 (3)//114.1
O3–S1–N2	111.73 (10)//111.72	N3–C13–N2	115.6 (3)//116.5
N2–C18–S2	130.88 (18)//131.67	C11–N1–C12	126.4 (3)//125.1
N3–C18–S2	109.21 (18)//108.18	N4–C12–N2	128.6 (3)//127.8
O2–S1–C15	108.12 (11)//107.52	—	—
O3–S1–C15	107.28 (10)//107.81	—	—
N2–S1–C15	106.06 (10)//103.68	—	—
N2–C18–N3	119.9 (2)//120.15	—	—

**Table:** Hydrogen-bond geometry (Å, °) in DSPIN and ACPIN, X-ray//DFT

DSPIN	D–H···A	D–H	H⋯A	D⋯A	∠(D–H⋯A)°
	N1–H1⋯O1	0.98 (2)//1.03	1.72 (2)//1.74	2.530 (3)//2.594	138 (2)//137
	N3–H3*A*⋯S1^a^	0.89 (2)//1.01	2.89 (3)	3.746 (2)	163 (2)
	N3–H3*A*⋯O2^a^	0.89 (2)//1.01	2.50 (2)	3.185 (3)	134 (2)
	N3–H3*A*⋯N2^a^	0.89 (2)//1.01	2.07 (2)	2.922 (3)	161 (2)
	C11–H11⋯O3^b^	0.93//1.08	2.64	3.521 (3)	157
	C13–H13⋯O3^b^	0.93//1.08	2.58	3.463 (3)	159
ACPIN	D–H···A	D–H	H⋯A	D⋯A	∠(D–H⋯A)°
	N1–H1⋯O1	0.86//1.03	1.86//1.81	2.557 (4)//2.613	137//132
	N3–H3*A*⋯N2^c^	0.86//1.01	2.19	3.037 (5)	171
	N3–H3*B*⋯O1^c^	0.86//1.01	2.38	3.003 (5)	129

a Symmetry codes: −*x* + 1, −*y* + 1, −*z* + 1.

b −*x* + 1, *y* + 1/2, −*z* + 1/2.

c −*x* + 3, −*y* + 1, −*z*.

In the zwitterion crystal structure named as (*E*)-1-(((4-amino-5-(4-chlorophenyl)-6-ethylpyrimidin-2-yl)iminio)methyl)naphthalen-2-olate, ACPIN (Fig. 1b and Table 1), the 1-methylnaphthalen-2-olate group A (C1–C11/O1), pyrimidine-2,4-diamine ring B (C12–C15/N1–N4), and chlorophenyl ring C (C18–C23/Cl1) are planar with r.m.s. deviations of 0.0264, 0.0303, and 0.0071 Å, respectively. The central ring is oriented at the dihedral angles of 8.85(2)° and 62.4(1)° with respect to the groups A and C, respectively. The atoms of the ethyl group (C16/C17) are at the distances of −0.0253(4) and 1.2138(8) Å, respectively, from the plane of the ring B. The molecular configuration is stabilized by intramolecular N–H⋯O bonding to form S(6) loop. The molecules of the compound ACPIN are also interlinked in the form of dimers through N–H⋯N bonding to complete *R*_2_^2^(8) loop, where donor NH is from the amino group and acceptor N-atom is from the pyrimidine ring (Fig. 2b and Table 3). The other H-atom of the amino group acts as H-bond donor for the O-atom and completes two bifurcated *R*_3_^2^(8) loops. No C–H⋯O bonding is found in the crystal packing. The ethyl group and one N-atom of the pyrimidine do not participate in the H-bonding. The crystal packing is further stabilized by off-set π⋯π stacking interactions between aromatic rings of the molecules that are related by inversion symmetry (Fig. 3b). The inter-centroid separation is 3.66 Å and ring off-set distance is 1.212 Å. The Cambridge structural database search provides one hint only with reference code SIYBUA. The crystal structure with the reference code SIYBUA was submitted to CCDC as CSD Communication (private communication) in 2019.^49^ It was crystallized in the monoclinic system with the space group P2_1_/*n*.

### 3.2. Hirshfeld surface analysis

**Fig. 4:**
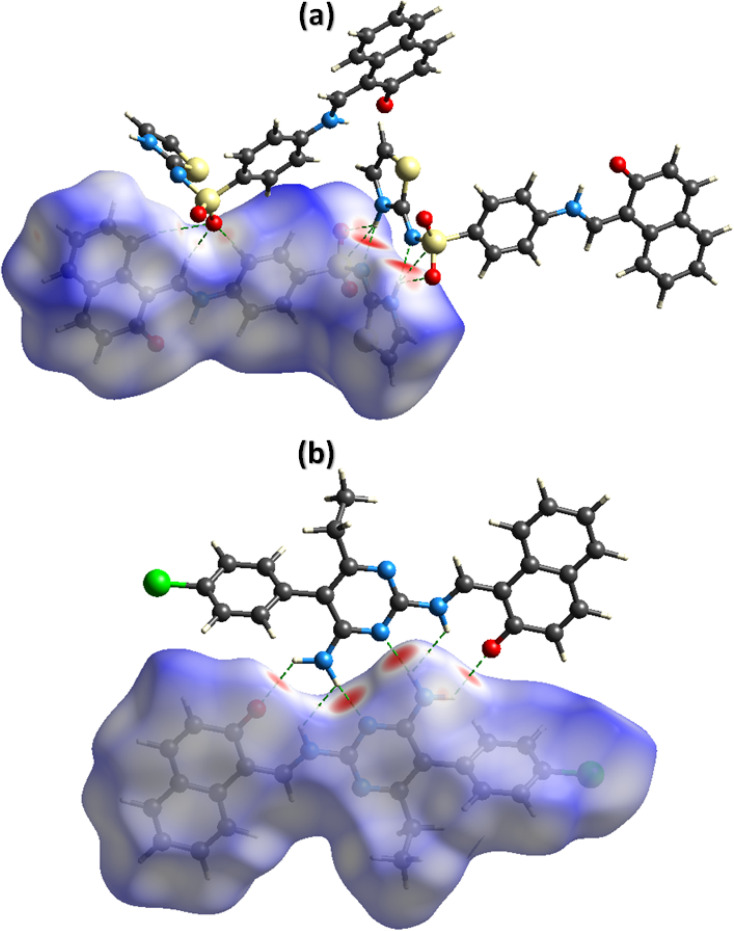
Hirshfeld surfaces plotted over *d*_norm_ for DSPIN in the range −0.5270 to 1.5020 a. u. (a) and ACPIN in the range −0.4528 to 1.8067 a. u. (b).

The noncovalent interactions represent the one of the most interesting topics for the researchers working in the field of supramolecular chemistry and drug design as the properties of the single crystals are defined by these interactions. Hirshfeld surface analysis was used for the inspection of the noncovalent interactions employing Crystal Explorer version 21.5.^50^ Hirshfeld surface is acquired by diving the space in the crystal into regions where promolecular electron density dominates over the procrystal electron density. Hirshfeld surface plotted over *d*_norm_ consists of three colours, red, white, and blue, to separate long-distance contacts from the other types of contacts.^51,52^ Red, white, and blue colours indicate the contacts with distance less than, equal to, and greater than sum of van der Waal radii. For DSPIN, the red spots on the surface near NH of the ring, O-atoms of the sulfonyl group, and N-atom directly bonded with the sulfonyl group indicate that these atoms form short contact with the neighbouring molecules (Fig. 4a). The NH group that links naphthalen-2-olate and phenyl ring is not involved in any intermolecular H-bonding. For ACPIN, red spots on the surface near O-atom of the naphthalen-2-olate, N-atoms of the pyrimidine ring, and N-atoms of the amino radical indicate that these atoms are engaged in H-bonding (Fig. 4b).

**Fig. 5:**
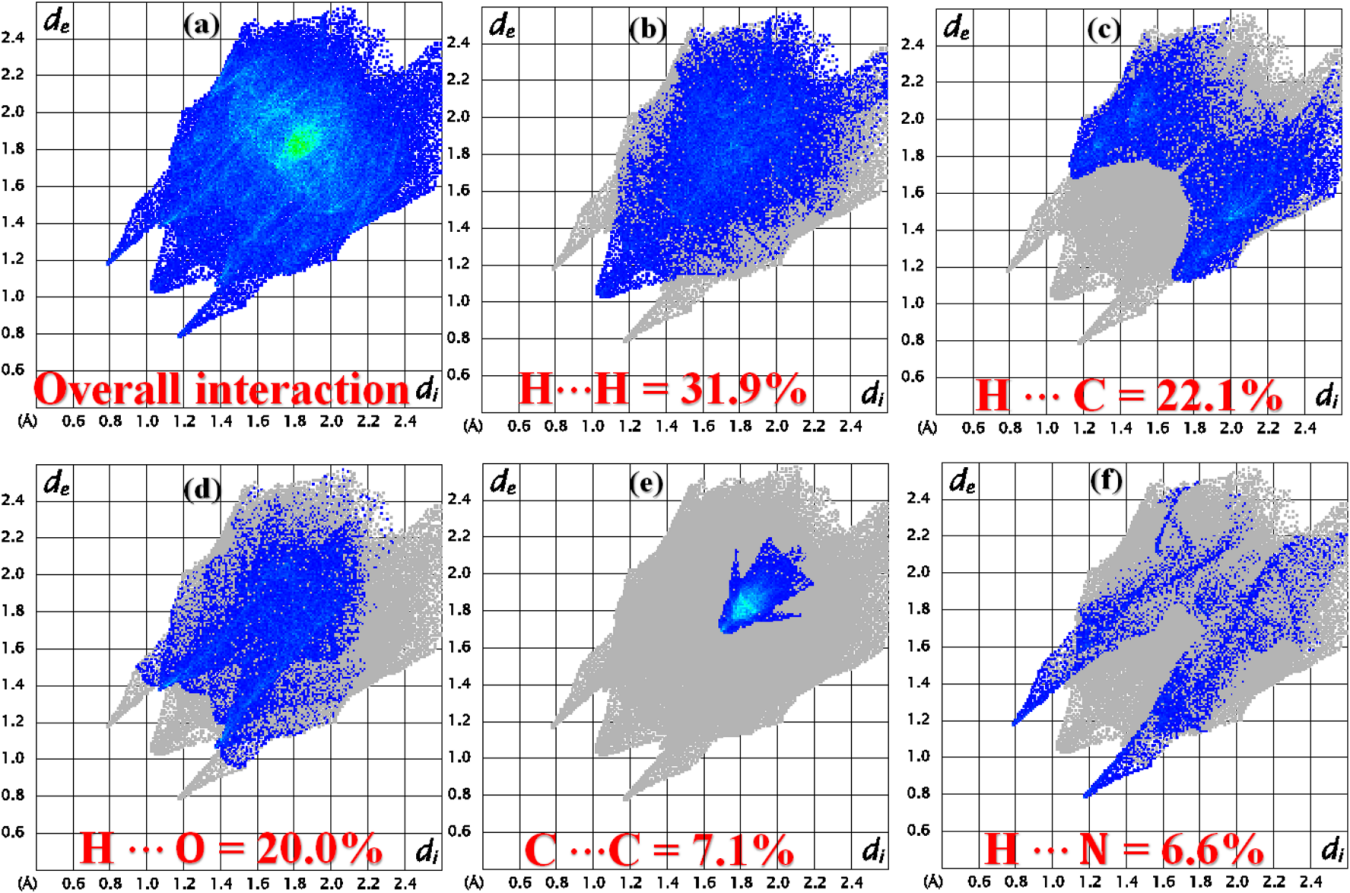
2D fingerprint plots for (a) overall interactions, (b–f) important individual interatomic contacts of DSPIN.

**Fig. 6:**
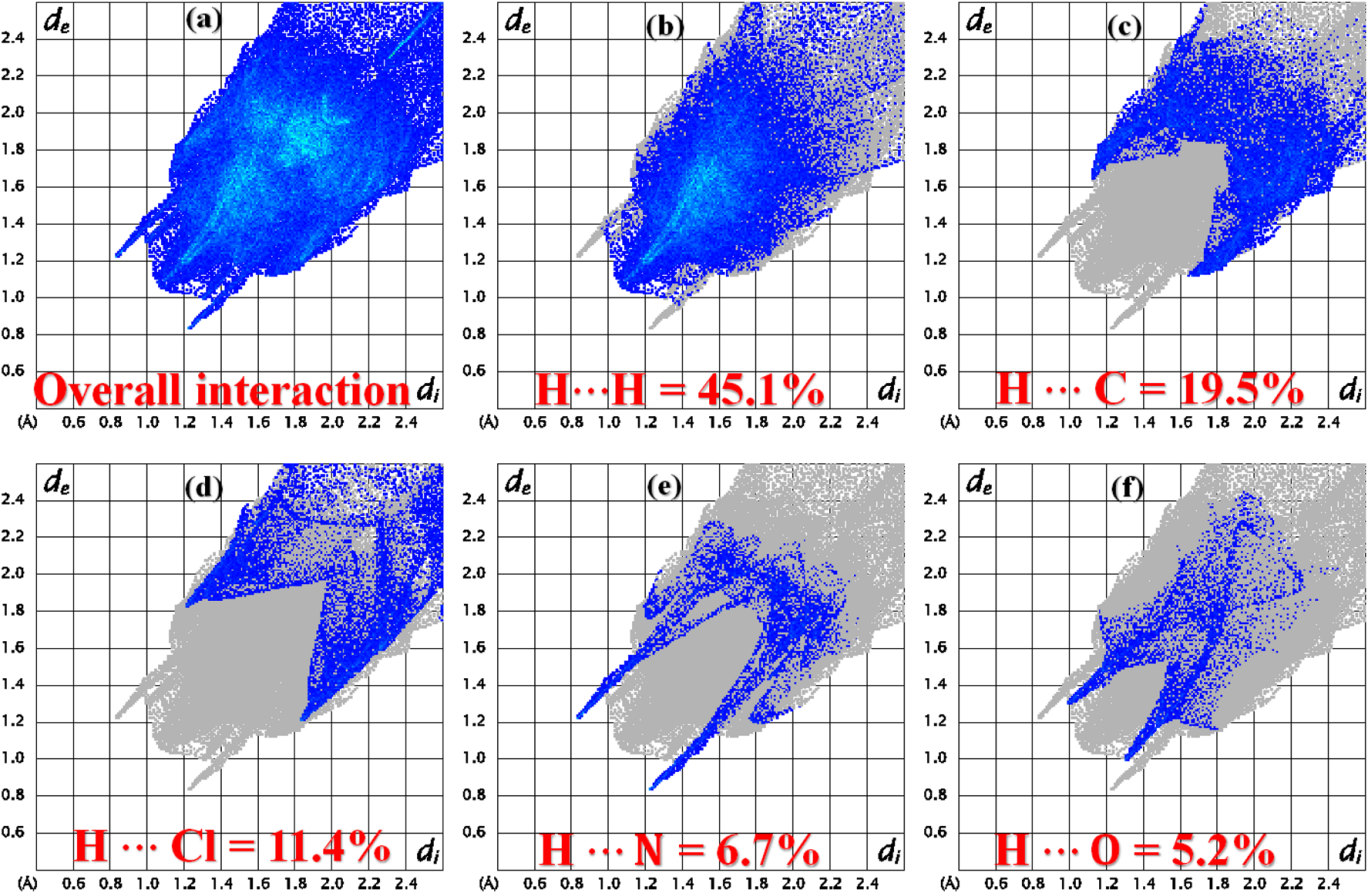
2D fingerprint plots for (a) overall interactions, (b–f) important individual interatomic contacts of ACPIN.

The role of the interatomic contacts in stabilizing the supramolecular assembly can be explored by 2D fingerprint plots because they provide the quantitative description which is usually remained hidden in the classic way of describing supramolecular assembly.^53,54^ The plot for overall interactions of DSPIN (Fig. 5a) is different from the corresponding 2D plot in ACPIN (Fig. 6a) showing that crystal packing of DSPIN is different from the crystal packing of ACPIN. Each individual contact is determined by including the reciprocal contacts. For DSPIN, the important interatomic contacts are H⋯H, H⋯C, H⋯O, C⋯C, and H⋯N with percentage contributions of 31.9%, 22.1%, 20%, 7.1%, and 6.6%, respectively. For ACPIN, the major contributors in crystal packing are H⋯H, H⋯C, H⋯Cl, H⋯N, and H⋯O with respective percentage contributions of 45.1%, 19.5%, 11.4%, 6.7%, and 5.2%, respectively. All the contacts have different probabilities to form crystal packing interactions. The probability of a contact to form crystal packing interactions is computed by finding enrichment ratio.^55,56^ Enrichment ratio is greater than 1 for the pair of chemical species that are favourable to form contacts and less than 0 for not favourable, respectively. For both compounds, C⋯C is the most favourable contact with the enrichment ratio 1.69 for DSPIN and 2.02 for ACPIN. H⋯H contact is not favourable in DSPIN (Table S1†) but is slightly favourable in ACPIN with the enrichment ratio slightly larger than one (Table S2†). H⋯N and H⋯S contacts are favourable for DSPIN whereas O⋯C, H⋯Cl, and H⋯N contacts are favourable for ACPIN.

**Fig. 7:**
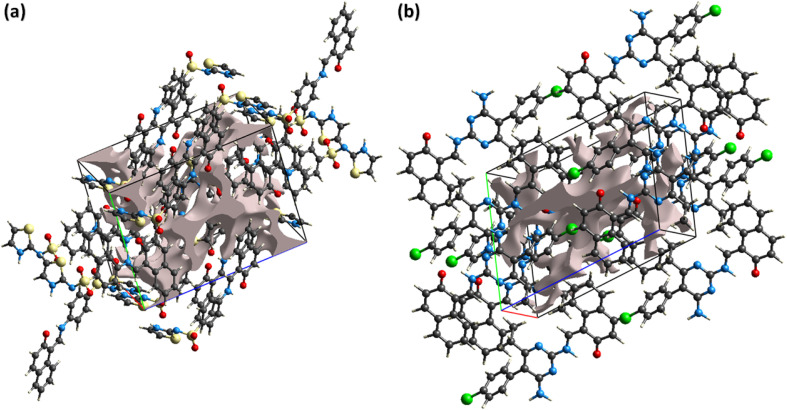
Graphical representation of voids in (a) DSPIN, (b) ACPIN.

The reaction of the crystal to applied stress can be investigated by calculating voids in the crystal. Crystals containing large cavities have poor response to applied stress, *i.e.*, such crystals can be broken by applying insignificant stress. For checking mechanical response of DSPIN and ACPIN, voids were calculated by adding the electron density of the atoms that are assumed to have spherical symmetry.^57,58^Fig. 7 shows the graphical view of voids in DSPIN and ACPIN. The calculated volumes of voids in DSPIN and ACPIN are 131.95 and 254.65 Å^3^, respectively. The space used by voids in supramolecular assembly of DSPIN and ACPIN is 13.7% and 13.3%, respectively. The packing index for DSPIN and ACPIN is 86.3% and 86.7%, respectively. In both compounds, the voids occupy a small amount of space, so the crystals have no large cavities and are expected to show good mechanical response.

**Fig. 8:**
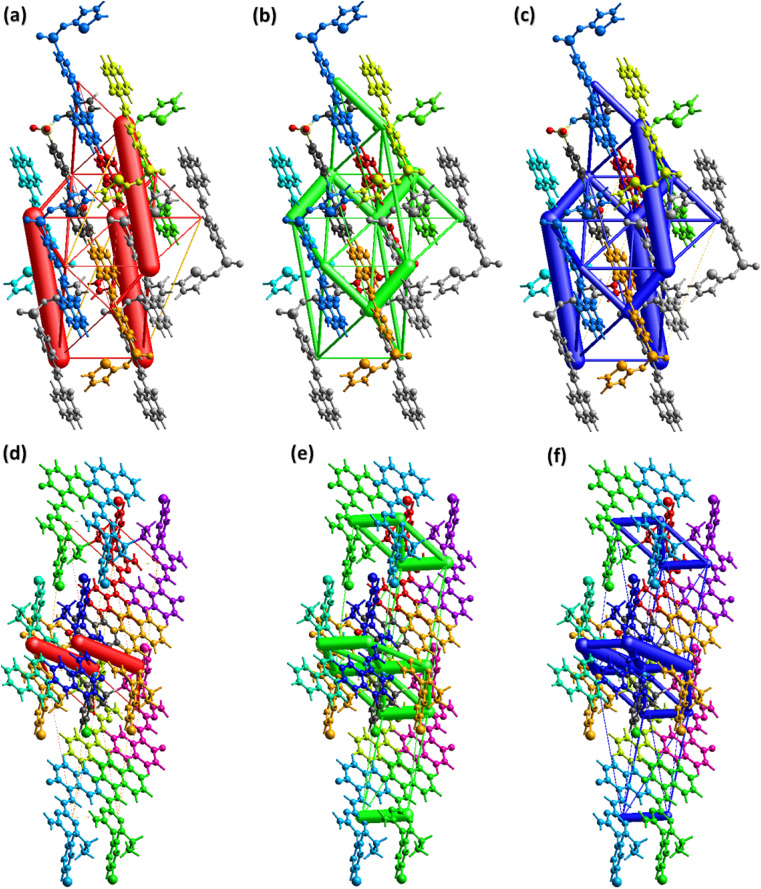
Energy frameworks for coulomb, dispersion, and total energy in (a–c) DSPIN, (d–f) ACPIN.

To carry out further investigation of the supramolecular assembly or crystal packing of DSPIN and ACPIN, interaction energies among the molecular pairs were calculated using the B3LYP/6-31G(d,p) electron density model as employed in TONTO built-in in Crystal Explorer version 21.5. A cluster of 3.8 Å around the molecule present in the asymmetric unit was included in interaction energy calculations. Total interaction energy is the sum of Coulomb electrostatic, dispersion, polarization, and repulsion energies.^59,60^ For organic crystals, Coulomb electrostatic and dispersion energies have dominant role in crystal packing as compared to other kinds of energies. The results of the interaction energy calculations are given in Tables S3 and S4† for DSPIN and ACPIN, respectively. The Coulomb energy is the largest for the molecular pair with intermolecular distances of 11.78 Å and 7.26 Å in DSPIN and ACPIN, respectively. The dispersion energy is the greatest for the molecular pair with intermolecular distances of 6.32 Å and 4.54 Å in DSPIN and ACPIN, respectively. In DSPIN, the Coulomb energy is repulsive for one pair with the intermolecular distance of 13.10 Å whereas in ACPIN the Coulomb energy is repulsive for two pairs with intermolecular distances of 4.54 and 17.67 Å. The pair with the largest total attractive energy contribution in the supramolecular assembly of DSPIN and ACPIN has the intermolecular distances of 11.78 and 7.26 Å, respectively. Fig. 8 shows the energy frameworks for Coulomb electrostatic, dispersion, and total energies for DSPIN and ACPIN. The molecular centers are joined by a cylinder whose width is directly proportional to the strength of the corresponding energy. Although the width of the cylinder for three molecular pairs is greater for the Coulomb energy framework of DSPIN but overall, the contribution of the dispersion energy in stabilization of the crystal packing is larger than the contribution of the Coulomb electrostatic energy. For both compounds, the dispersion energy is the dominant contribution in the crystal packing as compared to other kinds of energies.

### 3.3. DFT study results

#### 3.3.1. Structural features

**Fig. 9:**
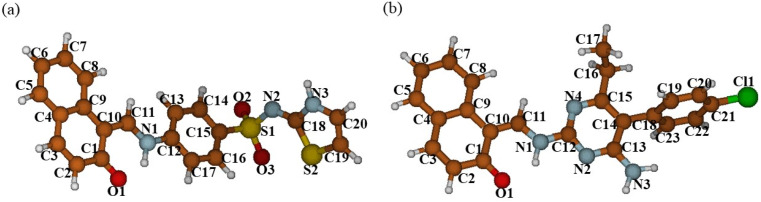
Structures of DSPIN (a) and ACPIN (b) optimized with the B3LYP/6-311+G(d,p) approach employing the implicit solvent effects from ethanol. Colour coding: brown for C, light grey for H, blue for N, red for O, dark yellow for S, and green for Cl. Numbering scheme corresponds to Fig. 1.

In Fig. 9 the structures of DSPIN and ACPIN optimized using the B3LYP/6-311+G(d,p) approach with the implicit solvent effects from C_2_H_5_OH are shown, and in Table 2 important bond lengths and bond angles for both compounds, both experimental and calculated, are provided. Comparison of the structural parameters in Table 2 shows reasonably good agreement between the X-ray diffraction and DFT results. Furthermore, in Table S5† more selected structural parameters are given, and in Table 3 several calculated structural parameters related with hydrogen bonding are provided along with the experimentally determined structural parameters. Consideration of the results for DSPIN in Table S5† shows relatively short H11–H13 interatomic distance, 2.07 Å, and even shorter interatomic distance H11–N1, 2.055 Å, along with noticeably longer interatomic distance H13–N1, 2.744 Å; another interatomic distance, N2–H3, is also relatively long, 2.534 Å, however, all three interatomic distances N–H suggest possible formation of intramolecular hydrogen bonding (see below discussion for NBO charges). Furthermore, comparison of the experimental and calculated geometrical parameters for the N1–H1⋯O1 hydrogen bond (Table 3) shows quite good agreement between the experimental and DFT results. Next, consideration of the results for ACPIN in Table S5† shows three interatomic distances N–H being within 2.394–2.468 Å, thus also implying possible formation of intramolecular hydrogen bonding (see below discussion for NBO charges). Moreover, comparison of the experimental and calculated structural parameters for the N1–H1⋯O1 hydrogen bond (see Table 3) again shows quite good agreement between the experimental and DFT results.

Consideration of the selected dihedral angles calculated for both compounds (Table S5†) shows the molecule of DSPIN being almost flat, the only dihedral angle noticeably different from 0 or 180 degrees is the ∠(H13–C13–C11–H1), *ca.* 13 degrees. The situation is different for the molecule of ACPIN, where the chlorophenyl group is rotated *vs.* the rest of the molecule, as supported by the calculated value of the ∠(C15–C14–C18–C19), *ca.* 76 degrees. It should be noticed that the differences observed between the experimentally determined and DFT optimized structures could be explained by the absence of the crystal structure restrictions in the calculations.

#### 3.3.2. Frontier molecular orbitals (FMOs) analysis

**Fig. 10:**
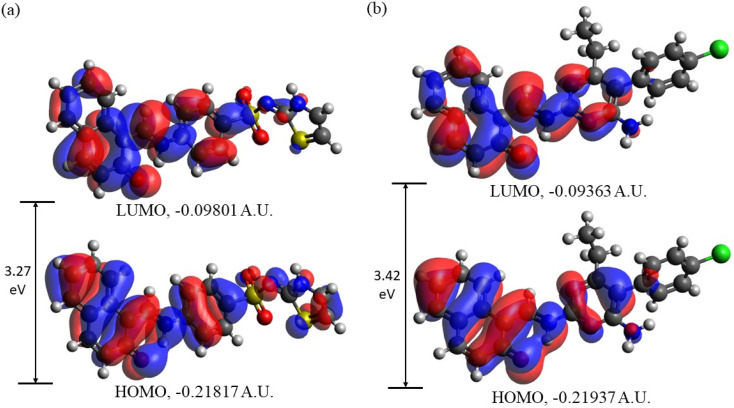
Frontier molecular orbitals of DSPIN (a) and ACPIN (b) computed with the B3LYP/6-311+G(d,p) approach employing the implicit solvent effects from ethanol.

In Fig. 10 the HOMOs and LUMOs of DSPIN and ACPIN computed using the B3LYP/6-311+G(d,p) approach with the implicit solvent effects from ethanol are shown, and Table 4 provides the energies of the selected MOs (HOMO−2–LUMO+2) of DSPIN and ACPIN (eV), the corresponding HOMO/LUMO gaps (eV), calculated at the B3LYP/6-311+G(d,p) level with the implicit effects from ethanol, along with the TDDFT gaps (eV). Analysis of the MOs plots and the results from Table 4 shows the following. (i) The DSPIN HOMO is contributed by essentially the whole molecule, with the dominating contributions from the 1-methylnaphthalen-2-olate and 4-aminobenzenethiol groups and much smaller contributions from the thiazol-2(3*H*)-imine group. The DSPIN LUMO is contributed mostly by contributions from the 1-methyl-naphthalen-2-olate and 4-aminobenzenethiol groups. Both HOMO and LUMO of ACPIN are contributed by the 1-methylnaphthalen-2-olate group and pyrimidine-2,4-diamine ring. Thus, in the electron transfer processes between HOMO and LUMO, in the case of DSPIN essentially the whole molecule is involved, whereas in the case of ACPIN mostly 1-methylnaphthalen-2-olate and 4-aminobenzenethiol groups of the molecule are involved. (ii) The DSPIN HOMO/LUMO gap is by 0.15 eV smaller than the ACPIN HOMO/LUMO gap due to some destabilization of the DSPIN HOMO and some stabilization of its LUMO. (iii) The HOMO−1/LUMO+1 and HOMO−2/LUMO+2 gaps for the compound DSPIN are also smaller compared to the compound ACPIN, by 0.38 and 0.07 eV, respectively. This implies that in the case of DSPIN the HOMO−1 and HOMO-2 are more likely to participate in redox reactions compared to ACPIN. The LUMO+1/LUMO+2 of both compounds are noticeably higher than their LUMOs, by 1.24/1.47 and 1.32/1.34 eV, respectively, and are much less likely to participate in redox reactions. (iv) The TDDFT gaps are, as expected, smaller than the HOMO/LUMO gaps, the TD-ωB97XD/6-311+G(d,p) gaps being larger than the TD-B3LYP/6-311+G(d,p) gaps, by 0.34 and 0.32 eV for DSPIN and ACPIN, respectively.

**Table:** Energies of the selected MOs (HOMO−2–LUMO+2) of DSPIN and ACPIN (eV), the corresponding HOMO/LUMO gaps (eV), calculated at the B3LYP/6-311+G(d,p) level with the implicit effects from ethanol, along with the TDDFT gaps (eV)

MOs	Energy, eV	Δ*E* (HOMO/LUMO), eV	Δ*E* (TDDFT-1)^a^, eV	Δ*E* (TDDFT-2)^b^, eV
**DSPIN**				
LUMO	−2.67	3.27	2.87	3.21
HOMO	−5.94			
LUMO+1	−1.43	5.01		
HOMO−1	−6.44			
LUMO+2	−1.20	5.50		
HOMO−2	−6.70			

**ACPIN**				
LUMO	−2.55	3.42	3.02	3.34
HOMO	−5.97			
LUMO+1	−1.23	5.39		
HOMO−1	−6.62			
LUMO+2	−1.21	5.57		
HOMO−2	−6.78			

a TD-B3LYP/6-311+G(d,p) approach.

b TD-ωB97XD/6-311+G(d,p) approach.

#### 3.3.3. Natural population analysis (NPA)

In Table 5 the NPA charges on the selected atoms of both compounds are provided. The values of these charges imply formation of intramolecular hydrogen bonds, N1–H1⋯O1 and N3–H3⋯N2 in DSPIN and N1–H1⋯O1, N1–H1⋯N2, N3–H3⋯N2, and C11–H11⋯N4 in ACPIN, thus supporting the results of the X-ray diffraction study and Hirshfeld analysis (see above). Furthermore, these charges suggest formation of intermolecular hydrogen bonding and dipole–dipole and dispersion interactions.

**Table:** NPA charges, *e*, on the selected atoms of the compounds DSPIN and ACPIN, computed at the B3LYP/6-311+G(d,p) level with the implicit effects from ethanol

Atom	Charge, *e*
**DSPIN**	
O1	−0.714
N1	−0.526
H1(N1)	0.450
S1	2.206
O2	−0.954
O3	−0.961
S2	0.409
N2	−0.858
N3	−0.548
H3(N3)	0.441

**ACPIN**	
Cl1	−0.012
O1	−0.711
N1	−0.547
H1(N1)	0.456
N2	−0.572
N4	−0.586
N3	−0.761
H3(N3)	0.411
H11(C11)	0.226

#### 3.3.4. Natural bonding orbital (NBO) analysis

The NBO analysis results provide us with knowledge of orbital interactions of different types, both intra- and intermolecular.^39^ The NBO analysis is performed by consideration of all possible interactions among the filled, or donor, Lewis-type NBOs and empty, or acceptor, non-Lewis NBOs. Their energetic contributions in interactions are evaluated using the 2nd-order perturbation theory. These interactions result in the decrease of the localized NBOs occupancy in the idealized Lewis structure and corresponding increase of the occupancy of the empty non-Lewis orbitals. As a consequence, they are referred to as ‘delocalization’ corrections to the 0^th^-order natural Lewis's structure. The stronger donor–acceptor interactions are characterized by higher stabilization energies. The 2nd-order stabilization energy *E*^(2)^ is computed according to eqn (7):7
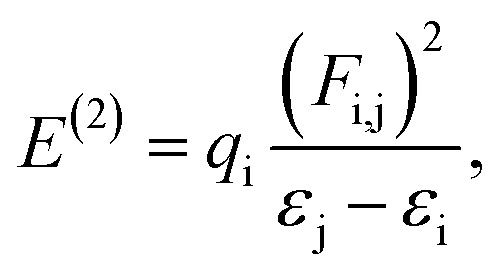
where *ε*_i_ and *ε*_j_ are off-diagonal and *F*_i. j_ is the diagonal elements of the NBO Fock matrix, *q*_i_ is the donor orbital possession, and *E*^(2)^ is the energy of stabilization.^39^

Second-order perturbation theory analysis results for both compounds, calculated using the B3LYP/6-311+G(d,p) approach with the implicit solvent effects from ethanol, are given in Table S6.† Analysis of these results shows the following. (i) There is a very large number of various donor–acceptor interactions in both compounds with stabilization energies in very broad range, from *ca.* 10 kcal mol^−1^ up to *ca.* 4100 kcal mol^−1^. (ii) In both compounds, numerous “remote” donor–acceptor interactions between different moieties of the molecule with significant stabilization energies can be noticed, *e.g.*: in DSPIN, σ(S2–C19)→π*(C13–C14), σ(N1–C11)→σ*(C16–H16), σ(N1–C12)→σ*(C16–H16), σ(N3–C18)→σ*(C16–H16), σ(N3–C20)→π*(C13–C14), σ(N3–C20)→σ*(C16–H16), σ(C1–C2)→π*(C13–C14), π(C7–C8)→σ*(C16–C17), σ(C19-20)→π*(C13–C14), σ(C20–H20)→π*(O1–C1), σ(C20–H20)→σ*(C8–H8), σ(C20–H20)→σ*(C16–C17), with the stabilization energies 488.92, 721.29, 3398.88, 1845.82, 969.38, 2881.41, 652.58, 311.99, 1909.55, 514.97, 483.59, and 1183.19 kcal mol^−1^, respectively; in ACPIN, σ(N1–C11)→σ*(C16–H16A), σ(N1–C11)→π*(C20–C21), σ(N1–C11)→σ*(C22–C23), σ(N1–C12)→σ*(C8–H8), σ(N1–C12)→σ*(C16–H16A), σ(N1–C12)→σ*(C16–H16B), σ(N1–C12)→σ*(C20–C21), σ(N1–C12)→σ*(C22–C23), σ(C18–C23)→σ*(C8–H8), σ(C18–C23)→σ*(C16–H16B), σ(C23–H23)→σ*(C4–C5), σ(C23–H23)→σ*(C10–C11), with the stabilization energies 213.17, 4137.13, 318.92, 404.57, 486.04, 3645.75, 1853.10, 854.05, 581.82, 604.48, 1349.18, and 654.23 kcal mol^−1^, respectively. (iii) Also, in both compounds the donor–acceptor interactions within the same moiety of the molecule with noticeable stabilization energies can be seen, *e.g.*: in DSPIN, σ(N3–C20)→σ*(N3–C18), σ(N3–C20)→σ*(C19–C20), π(C7–C8)→LP*(C10), π(C7–C8)→π*(O1–C1), π(C7–C8)→σ*(C8–H8), σ(C12–C17)→π*(C13–C14), with the stabilization energies 151.39, 207.91, 130.61, 131.51, 170.45, and 333.15 kcal mol^−1^, respectively; in ACPIN, π(C2–C3)→LP*(C1), π(C10–C11)→LP*(C1), σ(C18–C23)→σ*(C22–C23), σ(C22–H22)→π*(C18–C19), σ(C22–C23)→σ*(C22–C23), LP(O1)→LP*(C1), with the stabilization energies 169.24, 233.38, 389.83, 225.24, 832.74, and 1242.28 kcal mol^−1^, respectively.

Thus, as can be seen, molecules of both compounds have numerous stabilizing donor–acceptor interactions with noticeable stabilization energies, which would imply high stability of the compounds.

#### 3.3.5. Global reactivity parameters (GRPs) analysis

The global reactivity parameters, ionization potential (IP), electron affinity (EA), global softness (*σ*), global electrophilicity (*ω*), global hardness (*η*), global electronegativity (*X*), and chemical potential (*μ*), were computed using the FMOs energies (Table 2) according to eqn (1)–(6) (see Computational details),^40–42^ and the computed values in eV are presented in Table 6.

**Table:** The calculated GRPs for the compounds DSPIN and ACPIN (eV), computed at the B3LYP/6-311+G(d,p) level with the implicit effects from ethanol

IP	EA	Gap	*X*	*η*	*μ*	*σ*	*ω*
**DSPIN**							
5.94	2.67	3.27	4.305	1.635	−4.305	0.306	5.668

**ACPIN**							
5.97	2.55	3.42	4.260	1.710	−4.260	0.292	5.306

Analysis of the calculated GRPs shows the following. (i) Both compounds have significant values of IPs, 5.94 and 5.97 eV, and noticeable values of EAs, 2.67 and 2.55 eV. This implies that both compounds should be quite stable in oxidation processes, that is, would be poor electron donors, but quite good electron acceptors. (ii) The global hardness *η* values for both compounds, 1.635 and 1.710 eV, should be considered as quite noticeable, whereas their global softness *σ* values are quite small, 0.306 and 0.292 eV. This implies that both compounds should have quite low reactivity and be quite stable thermodynamically, DSPIN being somewhat more reactive than ACPIN. This is also supported by quite noticeable HOMO/LUMO gap values for both compounds, DSPIN having small HOMO/LUMO gap than ACPIN. The noticeable stability of both compounds is further supported by significant values of their chemical potentials, −4.305 and −4.260 eV. (iii) The global electronegativity *X* values for both compounds are high, 4.305 and 4.260 eV, as well as their global electrophilicity *ω* values, 5.668 and 5.306 eV. This implies that both compounds should be good electron acceptors and poor electron donors in redox reactions, which is in line with their IP and EA values.

Thus, from the GRP analysis, it follows that both compounds should be relatively nonreactive and thermodynamically stable. Moreover, they should act as should be good electron acceptors and poor electron donors in redox reactions.

#### 3.3.6. Molecular electrostatic potential (MEP) mapping

**Fig. 11:**
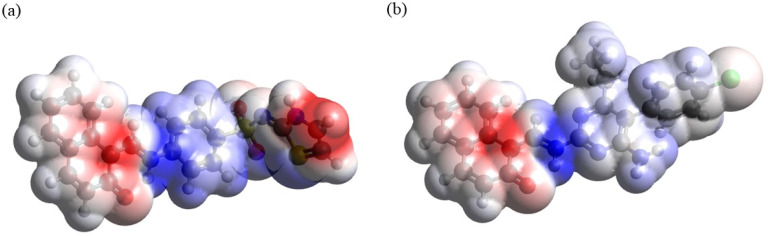
MEP plots of DSPIN (a) and ACPIN (b) computed with the B3LYP/6-311+G(d,p) approach employing the implicit solvent effects from ethanol.

Analysis of the MEP plots presented in Fig. 11 shows the following. (i) In both compounds, the accumulation of negative electrostatic potential (as indicated by red color) can be seen in the naphthalenone moiety. (ii) Also, in both compounds noticeable accumulation of positive electrostatic potential (as indicated by blue color) can be seen around the atoms C11–N1, and smaller accumulation of positive electrostatic potential can be seen at the 4-aminobenzenethiol moiety in DSPIN and at the pyrimidine-2,4-diamine moiety and phenyl ring in ACPIN. (iii) In DSPIN noticeable accumulation of negative electrostatic potential can be observed at the thiazol-2(3*H*)-imine, whereas in ACPIN only weak accumulation of negative electrostatic potential can be seen on the chlorine atom.

Thus, both compounds have pronounced areas of accumulation of both positive and negative electrostatic potential, which implies noticeable intermolecular interactions in crystals of these compounds, in line with the Hirshfeld analysis results.

## 4. Conclusions

Two new organic zwitterionic compounds DSPIN and ACPIN have been prepared *via* the condensation reaction. The structures of both compounds having crystalline nature were investigated and confirmed *via* SC-XRD studies which showed that the molecules were mainly connected in the form of dimers through N–H⋯N bonding in both compounds. Further stabilization of the supramolecular assembly was due to noncovalent interactions (N-H⋯S, N–H⋯O, C–H⋯O) in DSPIN and N–H⋯O interaction in ACPIN. Hirshfeld surface analysis was carried out to further investigate the noncovalent interactions showing that H⋯H contacts were the most significant contributor in the crystal packing of both compounds with percentage contributions of 31.9% in DSPIN and 45.1% in ACPIN. Void analysis inferred that both compounds would have good mechanical response. Interaction energy calculations showed that the dispersion energy was the prominent contributor for the stabilization of the supramolecular assembly in both compounds. DFT calculated geometries showed reasonably good agreement with the experimentally determined structural parameters. MOs analysis showed that in the electron transfer processes between HOMO and LUMO, in the case of DSPIN essentially the whole molecule would be involved, whereas in the case of ACPIN mostly 1-methylnaphthalen-2-olate and 4-aminobenzenethiol groups of the molecule would be involved. The DSPIN HOMO/LUMO gap was found to be by 0.15 eV smaller than the ACPIN HOMO/LUMO gap due to some destabilization of the DSPIN HOMO and some stabilization of its LUMO. In the case of DSPIN the HOMO−1 and HOMO−2 are more likely to participate in redox reactions compared to ACPIN. The LUMO+1 and LUMO+2 of both compounds are much less likely to participate in redox reactions. The results of the NPA analysis imply formation of intramolecular hydrogen bonds, thus supporting the results of the X-ray diffraction study and Hirshfeld analysis and suggest formation of intermolecular hydrogen bonding and dipole–dipole and dispersion interactions. The NBO analysis results showed that molecules of both compounds have numerous stabilizing donor–acceptor interactions with noticeable stabilization energies, which would imply high stability of the compounds. From the GRP analysis, it followed that both compounds should be relatively nonreactive and thermodynamically stable. Moreover, they should act as good electron acceptors and poor electron donors in redox reactions. The MEP analysis showed that both compounds have pronounced areas of accumulation of both positive and negative electrostatic potential, which implies noticeable intermolecular interactions in crystals of these compounds, in line with the Hirshfeld analysis results.

## Conflicts of interest

There are no conflicts to declare.

## Supplementary Material

RA-014-D3RA08727A-s001

RA-014-D3RA08727A-s002
