# Supplementary material for: A synthetic approach towards drug modification: 2-hydroxy-1-naphthaldehyde based imine-zwitterion preparation, single-crystal study, Hirshfeld surface analysis, and computational investigation
Source: RSC Adv. 2024 Feb 21;14(10):6476–93. doi: 10.1039/d3ra08727a (PMC10879849; doi:10.1039/d3ra08727a)
Supplement: RA-014-D3RA08727A-s001 [file RA-014-D3RA08727A-s001.pdf]

Electronic Supplementary Material (ESI) for RSC Advances.
This journal is © The Royal Society of Chemistry 2024

Supplementary Information File

A synthetic approach towards drugs modification: 2-hydroxy-1-naphthaldehyde based imine-zwitterion preparation, single-crystal study, Hirshfeld surface analysis, and computational investigation

Abida Naseem Malik,[a] Akbar Ali,*[b] Muhammad Ashfaq,*[a] Muhammad Nawaz Tahir,[a] Mohammad Mahtab Alam,[c] Mohamed S. Mostafa,[d] Aleksey Kuznetsov*[e]

[a] Department of Physics, University of Sargodha, Sargodha 40100, Pakistan

[b] Department of Chemistry, Government College University Faisalabad, 38000-Faisalabad Pakistan

[c] Department of Basic Medical Sciences, College of Applied Medical Science, King Khalid University, Abha 61421, Saudi Arabia.

[d] Department of Chemistry, Faculty of Science, Jazan University, P.O. Box 114, Jazan 45142, Saudi Arabia

[e] Departamento de Química, Campus Santiago Vitacura, Universidad Tecnica Federico Santa María, Av. Santa María 6400 Vitacura, 7660251, Chile

Contents:

1. Table S1: Enrichment ratio for the pair of chemical species in DSPIN. Enrichment ratio is not calculated for the pairs which random contact less than 0.99.

2. Table S2: Enrichment ratio for the pair of chemical species in ACPIN. Enrichment ratio is not calculated for the pairs which random contact less than 0.99.

3. Table S3: Interaction energies between molecular pairs in DSPIN.

4. Table S4: Interaction energies between molecular pairs in ACPIN.

5. checkCIF/PLATON report of DSPIN.

6. checkCIF/PLATON report of ACPIN.

7. Table S5: Selected structural parameters for the compounds DSPIN and ACPIN, calculated using the B3LYP/6-311+G(d,p) approach with the implicit solvent effects from ethanol.

8. Table S6. Second-order perturbation theory analysis results for the compounds DSPIN and ACPIN, calculated using the B3LYP/6-311+G(d,p) approach with the implicit solvent effects from ethanol.

Table S1: Enrichment ratio for the pair of chemical species in **DSPIN**. Enrichment ratio is not calculated for the pairs which random contact less than 0.99.

	Atom	H	C	N	O	S
**Contact %**	**H**	31.9	22.1	6.6	20	5.6
	**C**	22.1	7.1	1.2	3.5	0
	**N**	6.6	1.2	0	0.2	0
	**O**	20	3.5	0.2	0	1.8
	**S**	5.6	0	0	1.8	0
**Surface%**		59.05	20.5	4	12.75	3.7
**Random contacts %**	**Atom**	**H**	**C**	**N**	**O**	**S**
	**H**	34.87				
	**C**	24.21	4.20			
	**N**	4.72	1.64	0.16		
	**O**	15.06	5.23	1.02	1.63	
	**S**	4.37	1.52	0.30	0.94	0.14
**Enrichment ratio**	**Atom**	**H**	**C**	**N**	**O**	**S**
	**H**	0.91				
	**C**	0.91	1.69			
	**N**	1.40	0.73			
	**O**	1.33	0.67	0.20	0.00	
	**S**	1.28	0.00			

Table S2: Enrichment ratio for the pair of chemical species in **ACPIN**. Enrichment ratio is not calculated for the pairs which random contact less than 0.99.

	**Atom**	**H**	**C**	**N**	**O**	**Cl**
**Contact %**	**H**	45.1	19.5	6.7	5.2	11.4
	**C**	19.5	6.4	1.3	1.7	0.3
	**N**	6.7	1.3	0	0.3	0.1
	**O**	5.2	1.7	0.3	0.4	0.1
	**Cl**	11.4	0.3	0.1	0.1	1.5
**Surface%**		66.5	17.8	4.2	4.05	7.45
**Random contacts %**	**Atom**	**H**	**C**	**N**	**O**	**Cl**
	**H**	44.22				
	**C**	23.67	3.17			
	**N**	5.59	1.50	0.18		
	**O**	5.39	1.44	0.34	0.16	
	**Cl**	9.91	2.65	0.63	0.60	0.56
**Enrichment ratio**	**Atom**	**H**	**C**	**N**	**O**	**Cl**
	**H**	1.02				
	**C**	0.82	2.02			
	**N**	1.20	0.87			
	**O**	0.97	1.18			
	**Cl**	1.15	0.11			

Table S3: Interaction energies between molecular pairs in **DSPIN**.

Crystal | Atoms | Surface | Energies

Interaction Energies (kJ/mol)
R is the distance between molecular centroids (mean atomic position) in Å.

Total energies, only reported for two benchmarked energy models, are the sum of the four energy components, scaled appropriately (see the scale factor table below)

	N	Symop	R	Electron Density	E_ele	E_pol	E_dis	E_rep	E_tot
	1	-x, -y, -z	7.34	B3LYP/6-31G(d,p)	-8.1	-3.6	-66.8	35.7	-47.3
	2	-x, y+1/2, -z+1/2	16.26	B3LYP/6-31G(d,p)	-3.0	-0.5	-9.4	0.0	-11.7
	2	-x, y+1/2, -z+1/2	9.29	B3LYP/6-31G(d,p)	-6.6	-3.2	-17.4	9.8	-18.5
	1	x, y, z	7.84	B3LYP/6-31G(d,p)	-8.2	-3.5	-8.9	2.4	-17.5
	0	-x, -y, -z	13.10	B3LYP/6-31G(d,p)	7.4	-1.7	-5.1	0.0	2.1
	1	-x, -y, -z	6.32	B3LYP/6-31G(d,p)	-19.9	-7.5	-99.0	56.8	-77.7
	2	x, -y+1/2, z+1/2	10.73	B3LYP/6-31G(d,p)	-15.8	-5.0	-16.5	23.9	-20.1
	0	-x, y+1/2, -z+1/2	7.93	B3LYP/6-31G(d,p)	-21.3	-8.9	-33.4	29.0	-40.2
	2	x, -y+1/2, z+1/2	11.34	B3LYP/6-31G(d,p)	-2.5	-1.5	-20.1	11.2	-14.3
	0	-x, -y, -z	11.78	B3LYP/6-31G(d,p)	-121.0	-29.5	-30.7	99.1	-115.3

Scale factors for benchmarked energy models
See Mackenzie et al. IUCrJ (2017)

Energy Model	k_ele	k_pol	k_disp	k_rep
CE-HF ... HF/3-21G electron densities	1.019	0.651	0.901	0.811
CE-B3LYP ... B3LYP/6-31G(d,p) electron densities	1.057	0.740	0.871	0.618

Table S4: Interaction energies between molecular pairs in **ACPIN**.

Interaction Energies (kJ/mol)
R is the distance between molecular centroids (mean atomic position) in Å.

Total energies, only reported for two benchmarked energy models, are the sum of the four energy components, scaled appropriately (see the scale factor table below)

	N	Symop	R	Electron Density	E_ele	E_pol	E_dis	E_rep	E_tot
	1	-x, -y, -z	18.35	B3LYP/6-31G(d,p)	-0.7	-0.3	-11.3	0.0	-10.8
	2	x, y, z	4.54	B3LYP/6-31G(d,p)	1.2	-4.4	-91.7	43.7	-54.9
	1	-x, -y, -z	6.34	B3LYP/6-31G(d,p)	-11.4	-2.4	-52.1	28.7	-41.5
	2	x, y, z	18.31	B3LYP/6-31G(d,p)	-1.0	-0.1	-4.5	0.0	-5.0
	1	-x, -y, -z	7.26	B3LYP/6-31G(d,p)	-91.3	-21.3	-41.7	94.2	-90.4
	2	x, y, z	17.67	B3LYP/6-31G(d,p)	2.6	-0.2	-7.6	0.0	-4.0
	1	-x, -y, -z	6.25	B3LYP/6-31G(d,p)	-19.3	-7.1	-47.7	20.9	-54.3
	1	-x, -y, -z	18.64	B3LYP/6-31G(d,p)	0.0	-0.1	-7.9	0.0	-7.0
	1	-x, -y, -z	7.29	B3LYP/6-31G(d,p)	-6.7	-0.9	-47.7	29.1	-31.3

Scale factors for benchmarked energy models
See Mackenzie et al. IUCrJ (2017)

Energy Model	k_ele	k_pol	k_disp	k_rep
CE-HF ... HF/3-21G electron densities	1.019	0.651	0.901	0.811
CE-B3LYP ... B3LYP/6-31G(d,p) electron densities	1.057	0.740	0.871	0.618

checkCIF/PLATON report of DSPIN

Structure factors have been supplied for datablock(s) napsuly_0m

THIS REPORT IS FOR GUIDANCE ONLY. IF USED AS PART OF A REVIEW PROCEDURE FOR PUBLICATION, IT SHOULD NOT REPLACE THE EXPERTISE OF AN EXPERIENCED CRYSTALLOGRAPHIC REFEREE.

No syntax errors found.   CIF dictionary   Interpreting this report

Datablock: DSPIN.

Bond precision: C-C = 0.0034 A   Wavelength=0.71073

Cell:	a=7.8389(8)	b=13.8151(16)	c=17.198(2)
	alpha=90	beta=94.032(4)	gamma=90
Temperature:	296 K		

	Calculated	Reported
Volume	1857.9(4)	1857.9(4)
Space group	P 21/c	P 21/c
Hall group	-P 2ybc	-P 2ybc
Moiety formula	C20 H15 N3 O3 S2	C20 H15 N3 O3 S2
Sum formula	C20 H15 N3 O3 S2	C20 H15 N3 O3 S2
Mr	409.47	409.47
Dx,g cm-3	1.464	1.464
Z	4	4
Mu (mm-1)	0.314	0.314
F000	848.0	848.0
F000’	849.32	
h,k,lmax	10,17,21	9,17,21
Nref	4049	4040
Tmin,Tmax	0.904,0.930	0.869,0.894
Tmin’	0.904	

Correction method= # Reported T Limits: Tmin=0.869 Tmax=0.894

AbsCorr = MULTI-SCAN

Data completeness= 0.998 Theta(max)= 26.999

R(reflections)= 0.0437(2548) wR2(reflections)= 0.1087(4040)

S = 1.018 Npar= 259

The following ALERTS were generated. Each ALERT has the format

test-name_ALERT_alert-type_alert-level.

Click on the hyperlinks for more details of the test.

Alert level C

PLAT230_ALERT_2_C Hirshfeld Test Diff for S1 --O2 . 6.6 s.u.

PLAT334_ALERT_2_C Small <C-C> Benzene Dist. C12 -C17 . 1.37 Ang.

PLAT905_ALERT_3_C Negative K value in the Analysis of Variance ... -1.284 ReportPLAT911_ALERT_3_C Missing FCF Refl Between Thmin & STh/L= 0.600 2 Report

---

Alert level G

PLAT333_ALERT_2_G Large Aver C6-Ring C-C Dist C1 -C10 . 1.42 Ang.

PLAT432_ALERT_2_G Short Inter X...Y Contact O1 ..C19 . 3.00 Ang.

x,1/2-y,-1/2+z = 4_565 Check

PLAT480_ALERT_4_G Long H...A H-Bond Reported H3A ..S1 . 2.89 Ang.

PLAT480_ALERT_4_G Long H...A H-Bond Reported H11 ..O3 . 2.64 Ang.

PLAT910_ALERT_3_G Missing # of FCF Reflection(s) Below Theta(Min). 1 Note

PLAT912_ALERT_4_G Missing # of FCF Reflections Above STh/L= 0.600 6 Note

PLAT933_ALERT_2_G Number of HKL-OMIT Records in Embedded .res File 1 Note

PLAT941_ALERT_3_G Average HKL Measurement Multiplicity .... 3.8 Low

PLAT967_ALERT_5_G Note: Two-Theta Cutoff Value in Embedded .res .. 54.0 Degree

PLAT978_ALERT_2_G Number C-C Bonds with Positive Residual Density. 1 Info

---

0 **ALERT level A** = Most likely a serious problem - resolve or explain

0 **ALERT level B** = A potentially serious problem, consider carefully

4 **ALERT level C** = Check. Ensure it is not caused by an omission or oversight

10 **ALERT level G** = General information/check it is not something unexpected

0 ALERT type 1 CIF construction/syntax error, inconsistent or missing data

```
6 ALERT type 2 Indicator that the structure model may be wrong or deficient
4 ALERT type 3 Indicator that the structure quality may be low
3 ALERT type 4 Improvement, methodology, query or suggestion
1 ALERT type 5 Informative message, check
```

It is advisable to attempt to resolve as many as possible of the alerts in all categories. Often the minor alerts point to easily fixed oversights, errors and omissions in your CIF or refinement strategy, so attention to these fine details can be worthwhile. In order to resolve some of the more serious problems it may be necessary to carry out additional measurements or structure refinements. However, the purpose of your study may justify the reported deviations and the more serious of these should normally be commented upon in the discussion or experimental section of a paper or in the "special_details" fields of the CIF. checkCIF was carefully designed to identify outliers and unusual parameters, but every test has its limitations and alerts that are not important in a particular case may appear. Conversely, the absence of alerts does not guarantee there are no aspects of the results needing attention. It is up to the individual to critically assess their own results and, if necessary, seek expert advice.

Publication of your CIF in IUCr journals

A basic structural check has been run on your CIF. These basic checks will be run on all CIFs submitted for publication in IUCr journals (*Acta Crystallographica*, *Journal of Applied Crystallography*, *Journal of Synchrotron Radiation*); however, if you intend to submit to *Acta Crystallographica Section C* or *E* or *IUCrData*, you should make sure that full publication checks are run on the final version of your CIF prior to submission.

Publication of your CIF in other journals

Please refer to the *Notes for Authors* of the relevant journal for any special instructions relating to CIF submission.

PLATON version of 10/05/2023; check.def file version of 10/05/2023

Datablock napsuly_0m - ellipsoid plot

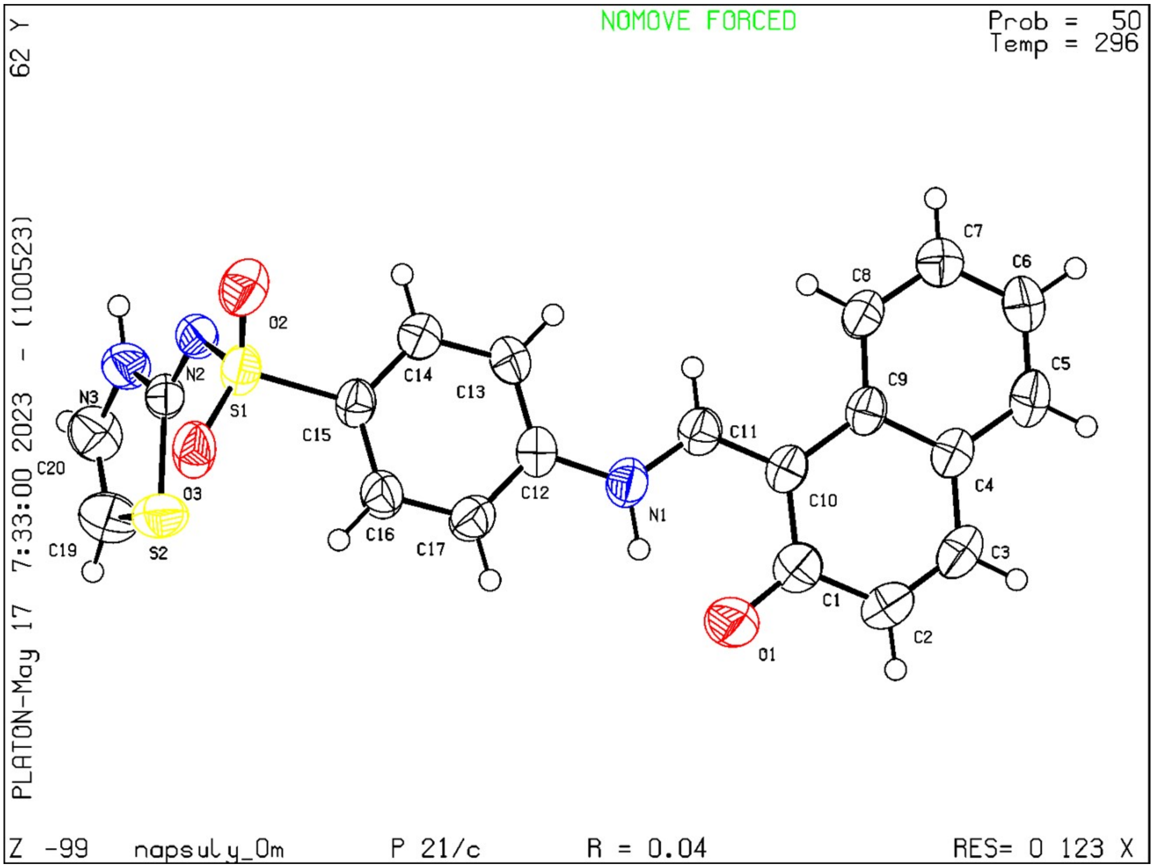

checkCIF/PLATON report of ACPIN.

Structure factors have been supplied for datablock(s) pymtvan_twnew

THIS REPORT IS FOR GUIDANCE ONLY. IF USED AS PART OF A REVIEW PROCEDURE FOR PUBLICATION, IT SHOULD NOT REPLACE THE EXPERTISE OF AN EXPERIENCED CRYSTALLOGRAPHIC REFEREE.

No syntax errors found. CIF dictionary Interpreting this report

Datablock: ACPIN

Bond precision:	C-C = 0.0057 A	Wavelength=0.71073	
Cell:	a=4.5436(6)	b=12.5255(16)	c=17.673(2)
	alpha=88.308(8)	beta=89.195(8)	gamma=82.611(9)
Temperature:	296 K		

	Calculated	Reported
Volume	997.0(2)	996.9(2)
Space group	P -1	P -1
Hall group	-P 1	-P 1
Moiety formula	C23 H19 Cl N4 O	C23 H19 Cl N4 O
Sum formula	C23 H19 Cl N4 O	C23 H19 Cl N4 O
Mr	402.87	402.87
Dx,g cm-3	1.342	1.324
Z	2	2
Mu (mm-1)	0.214	0.214
F000	420.0	420.0
F000'	420.44	
h,k,lmax	5,16,22	5,16,22
Nref	4543	4351
Tmin,Tmax	0.940,0.958	0.869,0.894
Tmin'	0.914	

Correction method= # Reported T Limits: Tmin=0.869 Tmax=0.894

AbsCorr = MULTI-SCAN

Data completeness= 0.958 Theta(max)= 27.448

R(reflections)= 0.0763(2397) wR2(reflections)= 0.2358(4351)

S = 1.139 Npar= 264

The following ALERTS were generated. Each ALERT has the format

test-name_ALERT_alert-type_alert-level.

Click on the hyperlinks for more details of the test.

Alert level C

DENSD01_ALERT_1_C The ratio of the submitted crystal density and that calculated from the formula is outside the range 0.99 <> 1.01Crystal density given = 1.324

Calculated crystal density = 1.342

PLAT029_ALERT_3_C _diffrn_measured_fraction_theta_full value Low . 0.966 Why?

PLAT046_ALERT_1_C Reported Z, MW and D(calc) are Inconsistent .... 1.342 Check

PLAT242_ALERT_2_C Low 'MainMol' Ueq as Compared to Neighbors of C21 Check

PLAT340_ALERT_3_C Low Bond Precision on C-C Bonds .... 0.00574 Ang.

PLAT906_ALERT_3_C Large K Value in the Analysis of Variance ...... 29.015 Check

PLAT906_ALERT_3_C Large K Value in the Analysis of Variance ...... 2.364 Check

PLAT911_ALERT_3_C Missing FCF Refl Between Thmin & STh/L= 0.600 121 Report

Alert level G

PLAT007_ALERT_5_G Number of Unrefined Donor-H Atoms .... 3 Report

PLAT333_ALERT_2_G Large Aver C6-Ring C-C Dist C1 -C10 . 1.43 Ang.

PLAT870_ALERT_4_G ALERTS Related to Twinning Effects Suppressed .. ! Info

PLAT910_ALERT_3_G Missing # of FCF Reflection(s) Below Theta(Min). 1 Note

PLAT912_ALERT_4_G Missing # of FCF Reflections Above STh/L= 0.600 70 Note

PLAT931_ALERT_5_G CIFcalcFCF Twin Law (0 0 1) Est.d BASF 0.18 Check

PLAT941_ALERT_3_G Average HKL Measurement Multiplicity .... 1.0 Low

0 **ALERT level A** = Most likely a serious problem - resolve or explain

0 **ALERT level B** = A potentially serious problem, consider carefully

8 **ALERT level C** = Check. Ensure it is not caused by an omission or oversight

7 **ALERT level G** = General information/check it is not something unexpected

```
2 ALERT type 1 CIF construction/syntax error, inconsistent or missing data
2 ALERT type 2 Indicator that the structure model may be wrong or deficient
7 ALERT type 3 Indicator that the structure quality may be low
2 ALERT type 4 Improvement, methodology, query or suggestion
2 ALERT type 5 Informative message, check
```

It is advisable to attempt to resolve as many as possible of the alerts in all categories. Often the minor alerts point to easily fixed oversights, errors and omissions in your CIF or refinement strategy, so attention to these fine details can be worthwhile. In order to resolve some of the more serious problems it may be necessary to carry out additional measurements or structure refinements. However, the purpose of your study may justify the reported deviations and the more serious of these should normally be commented upon in the discussion or experimental section of a paper or in the "special_details" fields of the CIF. checkCIF was carefully designed to identify outliers and unusual parameters, but every test has its limitations and alerts that are not important in a particular case may appear. Conversely, the absence of alerts does not guarantee there are no aspects of the results needing attention. It is up to the individual to critically assess their own results and, if necessary, seek expert advice.

Publication of your CIF in IUCr journals

A basic structural check has been run on your CIF. These basic checks will be run on all CIFs submitted for publication in IUCr journals (*Acta Crystallographica*, *Journal of Applied Crystallography*, *Journal of Synchrotron Radiation*); however, if you intend to submit to *Acta Crystallographica Section C* or *E* or *IUCrData*, you should make sure that full publication checks are run on the final version of your CIF prior to submission.

Publication of your CIF in other journals

Please refer to the *Notes for Authors* of the relevant journal for any special instructions relating to CIF submission.

PLATON version of 10/05/2023; check.def file version of 10/05/2023

Datablock pymtvan_twnew - ellipsoid plot

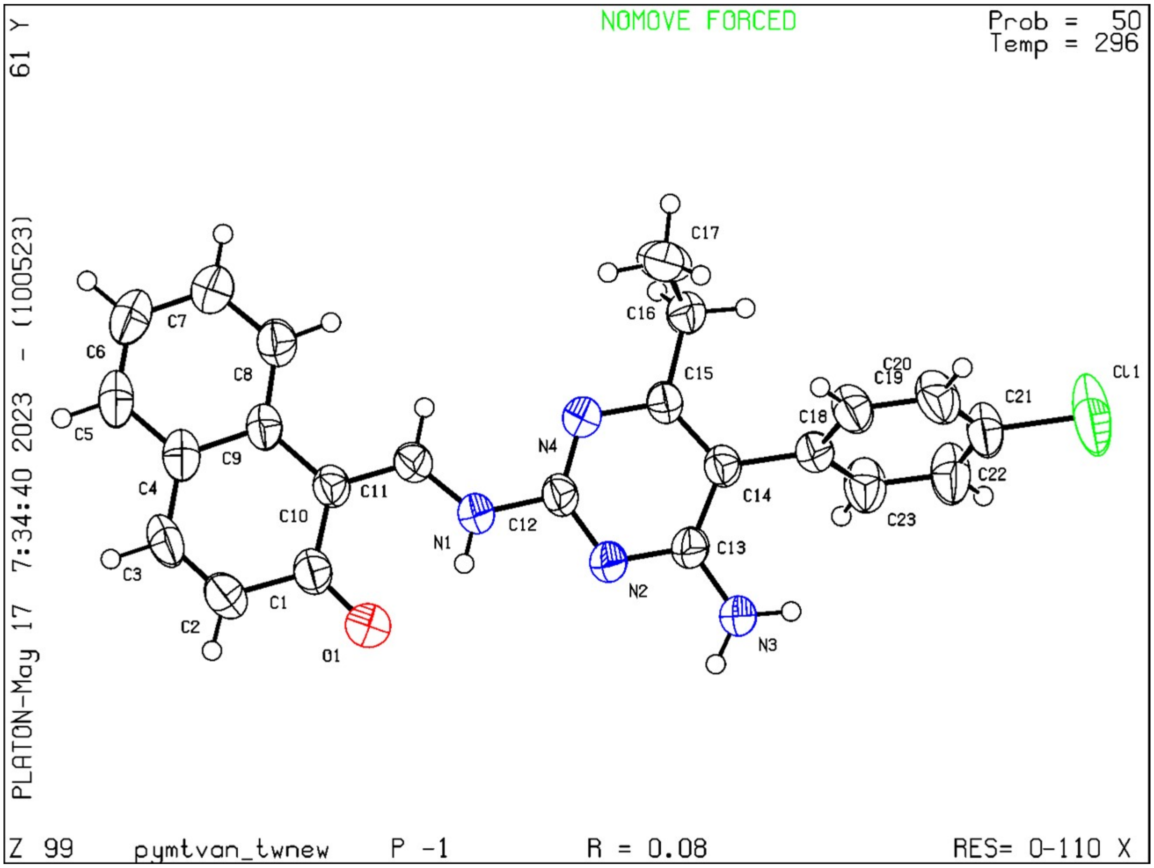

CIF of DSPIN without hkl data

data_global

#===

PROCESSING SUMMARY (IUCr Office Use only)

_publ_contact_author

;

Muhammad Nawaz Tahir

University of Sargodha

Department of Physics

Sargodha

Pakistan

;

_publ_contact_author_phone '0092 48 92 30 914'
_publ_contact_author_fax '0092 48 32 22 121'
_publ_contact_author_email 'dmntahir_uos@yahoo.com'
_publ_requested_journal ''
_journal_date_recd_electronic ?

_journal_date_to_coeditor ?
_journal_date_from_coeditor ?
_journal_date_accepted ?

_journal_date_printers_first ?
_journal_date_printers_final ?
_journal_date_proofs_out ?
_journal_date_proofs_in ?

_journal_coeditor_name ?
_journal_coeditor_code ?
_journal_paper_category ?
_journal_coeditor_notes
;
;

_journal_techeditor_code ?
_iucr_compatibility_tag ?
_journal_techeditor_notes
;
;

_journal_coden_ASTM ?
_journal_name_full ?
_journal_year ?
_journal_volume ?
_journal_issue ?

_journal_page_first ?

_journal_page_last ?

_journal_suppl_publ_number ?

_journal_suppl_publ_pages ?

#==

loop_

_publ_author_name

_publ_author_address

'Muhammad Ashfaq'

;

Department of Physics

University of Sargodha

Sargodha

Pakistan

orcid id: 0000-0001-6663-8777

;

'Muhammad Nawaz Tahir'

;

Department of Physics

University of Sargodha

Sargodha

Pakistan

orcid id: 0000-0002-6815-9806

;

'Akbar Ali'

;

Department of Chemistry,

Government College University

Faisalabad,

Pakistan.

;

'Abida Naseem Malik'
;
Department of Physics
University of Sargodha
Sargodha
Pakistan
;
'Aleksey Kuznetsov'
;
Departamento de Quimica,
Campus Santiago Vitacura,
Universidad Tecnica Federico Santa Maria,
Av. Santa Maria 6400 Vitacura,
Postal code: 7660251,
Chile
;

_audit_creation_date ?

_audit_creation_method ?

#==

TEXT

_publ_section_title

;

1-((Z)-((4-((E)-N-(thiazol-2(3H)-ylidene)sulfamoyl)phenyl)iminio)methyl

naphthalen-2-olate

;

_publ_section_abstract

;

;

_publ_section_comment

;

;

_publ_section_exptl_refinement

;

;

_publ_section_exptl_prep

;

;

_publ_section_related_literature

;

;

_publ_section_references

;

Bruker (2007). <i>SADABS</i>. Bruker AXS Inc., Madison, Wisconsin, USA.

Bruker (2007). <i>APEX2</i> and <i>SAINT</i>. Bruker AXS Inc.,
Madison, Wisconsin, USA.

Farrugia, L. J. (1997). <i>J. Appl. Cryst.</i> 30, 565.

Farrugia, L. J. (2012). <i>J. Appl. Cryst.</i> 45, 849--854.

Sheldrick, G. M. (2008). <i>Acta Cryst.</i> A64, 112--122.

Sheldrick, G. M. (2015). <i>Acta Cryst.</i> C71, 3--8.

Spek, A. L. (2009). <i>Acta Cryst.</i> D65, 148--155.

;

_publ_section_figure_captions

;

;

_publ_section_acknowledgements

```
;
;

data_napsuly_0m

_audit_creation_method            'SHELXL-2019/2'
_shelx_SHELXL_version_number      '2019/2'
_chemical_name_common     ?
_chemical_absolute_configuration   '.'
_chemical_name_systematic
;
1-((Z)-((4-((E)-N-(thiazol-2(3H)-ylidene)sulfamoyl)phenyl)iminio)methyl
naphthalen-2-olate
;
_chemical_formula_moiety    'C20 H15 N3 O3 S2'
_chemical_formula_sum       'C20 H15 N3 O3 S2'
_chemical_formula_iupac     'C20 H15 N3 O3 S2'
_chemical_formula_weight             409.47

loop_
 _atom_type_symbol
 _atom_type_description
 _atom_type_scat_dispersion_real
 _atom_type_scat_dispersion_imag
 _atom_type_scat_source
 'C'  'C'   0.0033   0.0016
 'International Tables Vol C Tables 4.2.6.8 and 6.1.1.4'
 'H'  'H'   0.0000   0.0000
 'International Tables Vol C Tables 4.2.6.8 and 6.1.1.4'
 'N'  'N'   0.0061   0.0033
 'International Tables Vol C Tables 4.2.6.8 and 6.1.1.4'
 'O'  'O'   0.0106   0.0060
 'International Tables Vol C Tables 4.2.6.8 and 6.1.1.4'
```

'S' 'S' 0.1246 0.1234
'International Tables Vol C Tables 4.2.6.8 and 6.1.1.4'

_space_group_crystal_system monoclinic
_space_group_IT_number 14
_space_group_name_H-M_alt 'P 21/c'
_space_group_name_Hall '-P 2ybc'

_shelx_space_group_comment
;
The symmetry employed for this shelxl refinement is uniquely defined
by the following loop, which should always be used as a source of
symmetry information in preference to the above space-group names.
They are only intended as comments.
;

loop_
_space_group_symop_operation_xyz
'x, y, z'
'-x, y+1/2, -z+1/2'
'-x, -y, -z'
'x, -y-1/2, z-1/2'

_cell_length_a 7.8389(8)
_cell_length_b 13.8151(16)
_cell_length_c 17.198(2)
_cell_angle_alpha 90
_cell_angle_beta 94.032(4)
_cell_angle_gamma 90
_cell_volume 1857.9(4)
_cell_formula_units_Z 4
_cell_measurement_reflns_used 2548
_cell_measurement_theta_min 2.374

_cell_measurement_theta_max 27.000

_cell_measurement_temperature 296(2)

_exptl_crystal_description prism

_exptl_crystal_colour 'red'

_exptl_crystal_size_max 0.32

_exptl_crystal_size_mid 0.28

_exptl_crystal_size_min 0.23

_exptl_crystal_density_diffrn 1.464

_exptl_crystal_density_meas ?

_exptl_crystal_density_method 'not measured'

_exptl_crystal_F_000 848

_exptl_absorpt_coefficient_mu 0.314

_exptl_absorpt_correction_type multi-scan

_exptl_absorpt_process_details '(SADABS; Bruker, 2007)'

_exptl_absorpt_correction_T_min 0.869

_exptl_absorpt_correction_T_max 0.894

_exptl_special_details

;

;

_diffrn_ambient_temperature 296(2)

_diffrn_radiation_type MoK\a

_diffrn_radiation_wavelength 0.71073

_diffrn_radiation_source 'fine-focus sealed tube'

_diffrn_radiation_monochromator graphite

_diffrn_measurement_device_type 'Bruker Kappa APEXII CCD'

_diffrn_measurement_method \w

_diffrn_detector_area_resol_mean 7.828

_diffrn_reflns_number 15333

_diffrn_reflns_av_unetI/netI 0.0473

_diffrn_reflns_av_R_equivalents 0.0404

_diffrn_reflns_limit_h_min -9

_diffrn_reflns_limit_h_max 9

_diffrn_reflns_limit_k_min -17

_diffrn_reflns_limit_k_max 15

_diffrn_reflns_limit_l_min -21

_diffrn_reflns_limit_l_max 21

_diffrn_reflns_theta_min 2.374

_diffrn_reflns_theta_max 26.999

_diffrn_reflns_theta_full 25.242

_diffrn_measured_fraction_theta_max 0.998

_diffrn_measured_fraction_theta_full 0.999

_diffrn_reflns_Laue_measured_fraction_max 0.998

_diffrn_reflns_Laue_measured_fraction_full 0.999

_diffrn_reflns_point_group_measured_fraction_max 0.998

_diffrn_reflns_point_group_measured_fraction_full 0.999

_diffrn_standards_number 0

_diffrn_standards_interval_count ?

_diffrn_standards_interval_time ?

_diffrn_standards_decay_% ?

_refine_special_details

;

Refinement of <i>F</i>^2^ against ALL reflections. The weighted <i>R</i>-factor

<i>wR</i> and goodness of fit <i>S</i> are based on <i>F</i>^2^, conventional

<i>R</i>-factors <i>R</i> are based on <i>F</i>, with <i>F</i> set to zero for

negative <i>F</i>^2^. The threshold expression of <i>F</i>^2^ >

\s(<i>F</i>^2^) is used only for calculating <i>R</i>-factors(gt) <i>etc</i>.

and is not relevant to the choice of reflections for refinement.

<i>R</i>-factors based on <i>F</i>^2^ are statistically about twice as large

as those based on <i>F</i>, and <i>R</i>- factors based on ALL data will be

even larger.

;

_reflns_number_total 4040

_reflns_number_gt 2548

_reflns_threshold_expression I>2\s(I)

_refine_ls_structure_factor_coef Fsqd

_refine_ls_matrix_type full

_refine_ls_R_factor_all 0.0852

_refine_ls_R_factor_gt 0.0437

_refine_ls_wR_factor_ref 0.1087

_refine_ls_wR_factor_gt 0.0916

_refine_ls_goodness_of_fit_ref 1.018

_refine_ls_restrained_S_all 1.018

_refine_ls_number_reflns 4040

_refine_ls_number_parameters 259

_refine_ls_number_restraints 0

_refine_ls_extinction_method none

_refine_ls_extinction_coef .

_refine_ls_hydrogen_treatment mixed

_refine_ls_weighting_scheme calc

_refine_ls_weighting_details

'w=1/[\s^2^(Fo^2^)+(0.0429P)^2^+0.3132P] where P=(Fo^2^+2Fc^2^)/3'

_atom_sites_solution_hydrogens geom

_atom_sites_solution_primary direct

_atom_sites_solution_secondary difmap

_refine_ls_shift/su_max 0.000

_refine_ls_shift/su_mean 0.000

_computing_data_collection 'APEX2 (Bruker, 2007)'

_computing_cell_refinement 'SAINT (Bruker, 2007)'

_computing_data_reduction 'SAINT (Bruker, 2007)'

_computing_structure_solution 'SHELXT2014 (Sheldrick, 2008)'

_computing_structure_refinement 'SHELXL-2019/2 (Sheldrick, 2015)'

_computing_molecular_graphics

'ORTEP-3 for Windows (Farrugia, 2012) and PLATON (Spek, 2009)'

_computing_publication_material

;

WinGX (Farrugia, 2012) and PLATON (Spek, 2009)

;

loop_
 _atom_site_label
 _atom_site_type_symbol
 _atom_site_fract_x
 _atom_site_fract_y
 _atom_site_fract_z
 _atom_site_U_iso_or_equiv
 _atom_site_adp_type
 _atom_site_occupancy
 _atom_site_site_symmetry_order
 _atom_site_calc_flag
 _atom_site_refinement_flags_posn
 _atom_site_refinement_flags_adp
 _atom_site_refinement_flags_occupancy
 _atom_site_disorder_assembly
 _atom_site_disorder_group
S1 S 0.62806(8) 0.37379(5) 0.35983(3) 0.04756(19) Uani 1 1 d . . . . .
S2 S 0.27019(9) 0.27664(5) 0.41242(4) 0.0608(2) Uani 1 1 d . . . . .
O1 O 0.1217(2) 0.41542(13) -0.06501(11) 0.0680(5) Uani 1 1 d . . . . .
O2 O 0.7889(2) 0.42300(13) 0.36955(9) 0.0608(5) Uani 1 1 d . . . . .
O3 O 0.6259(2) 0.27012(12) 0.36427(9) 0.0600(5) Uani 1 1 d . . . . .
N1 N 0.3001(2) 0.48144(15) 0.05142(11) 0.0486(5) Uani 1 1 d . . . . .
H1 H 0.238(3) 0.4297(17) 0.0229(14) 0.058 Uiso 1 1 d . U . . .
N2 N 0.5153(2) 0.42218(13) 0.42388(10) 0.0448(5) Uani 1 1 d . . . . .
N3 N 0.2837(3) 0.42242(15) 0.49937(12) 0.0508(5) Uani 1 1 d . . . . .
H3A H 0.322(3) 0.4750(18) 0.5249(14) 0.061 Uiso 1 1 d . U . . .
C1 C 0.1231(3) 0.49923(19) -0.09520(14) 0.0509(6) Uani 1 1 d . . . . .
C2 C 0.0363(3) 0.5141(2) -0.17026(14) 0.0572(7) Uani 1 1 d . . . . .
H2 H -0.019101 0.462165 -0.195502 0.069 Uiso 1 1 calc R U . . .
C3 C 0.0327(3) 0.6003(2) -0.20484(14) 0.0566(7) Uani 1 1 d . . . . .

H3 H -0.025917 0.606851 -0.253469 0.068 Uiso 1 1 calc R U . . .

C4 C 0.1162(3) 0.68335(18) -0.16976(13) 0.0479(6) Uani 1 1 d . . . . .

C5 C 0.1148(3) 0.7723(2) -0.20827(14) 0.0652(8) Uani 1 1 d . . . . .

H5 H 0.055085 0.778511 -0.256678 0.078 Uiso 1 1 calc R U . . .

C6 C 0.1997(4) 0.8502(2) -0.17602(15) 0.0700(8) Uani 1 1 d . . . . .

H6 H 0.197231 0.909269 -0.201978 0.084 Uiso 1 1 calc R U . . .

C7 C 0.2892(3) 0.8406(2) -0.10451(14) 0.0630(7) Uani 1 1 d . . . . .

H7 H 0.348124 0.893463 -0.082612 0.076 Uiso 1 1 calc R U . . .

C8 C 0.2926(3) 0.75469(18) -0.06548(13) 0.0528(6) Uani 1 1 d . . . . .

H8 H 0.353845 0.750163 -0.017294 0.063 Uiso 1 1 calc R U . . .

C9 C 0.2064(3) 0.67346(17) -0.09614(12) 0.0424(5) Uani 1 1 d . . . . .

C10 C 0.2088(3) 0.57993(17) -0.05741(12) 0.0422(5) Uani 1 1 d . . . . .

C11 C 0.2919(3) 0.56653(18) 0.01579(12) 0.0460(6) Uani 1 1 d . . . . .

H11 H 0.343968 0.619554 0.040893 0.055 Uiso 1 1 calc R U . . .

C12 C 0.3795(3) 0.46035(17) 0.12503(12) 0.0433(5) Uani 1 1 d . . . . .

C13 C 0.4622(3) 0.52816(18) 0.17266(13) 0.0536(6) Uani 1 1 d . . . . .

H13 H 0.467179 0.592349 0.156691 0.064 Uiso 1 1 calc R U . . .

C14 C 0.5374(3) 0.50080(18) 0.24386(13) 0.0542(6) Uani 1 1 d . . . . .

H14 H 0.594324 0.546593 0.275698 0.065 Uiso 1 1 calc R U . . .

C15 C 0.5292(3) 0.40645(17) 0.26843(12) 0.0422(5) Uani 1 1 d . . . . .

C16 C 0.4473(3) 0.33821(17) 0.22109(13) 0.0494(6) Uani 1 1 d . . . . .

H16 H 0.442650 0.274010 0.237083 0.059 Uiso 1 1 calc R U . . .

C17 C 0.3723(3) 0.36588(18) 0.14980(13) 0.0501(6) Uani 1 1 d . . . . .

H17 H 0.315809 0.319999 0.117875 0.060 Uiso 1 1 calc R U . . .

C18 C 0.3718(3) 0.38159(16) 0.44415(12) 0.0431(5) Uani 1 1 d . . . . .

C19 C 0.1106(3) 0.2962(2) 0.47421(17) 0.0673(8) Uani 1 1 d . . . . .

H19 H 0.016556 0.255785 0.477583 0.081 Uiso 1 1 calc R U . . .

C20 C 0.1373(3) 0.3750(2) 0.51617(16) 0.0615(7) Uani 1 1 d . . . . .

H20 H 0.064756 0.396229 0.553157 0.074 Uiso 1 1 calc R U . . .

loop_

_atom_site_aniso_label

_atom_site_aniso_U_11

_atom_site_aniso_U_22

_atom_site_aniso_U_33

_atom_site_aniso_U_23

_atom_site_aniso_U_13

_atom_site_aniso_U_12

S1 0.0609(4) 0.0475(4) 0.0340(3) 0.0018(3) 0.0011(3) 0.0097(3)

S2 0.0707(5) 0.0502(4) 0.0613(4) -0.0118(3) 0.0032(3) -0.0108(3)

O1 0.0838(13) 0.0467(12) 0.0711(12) -0.0001(9) -0.0100(10) 0.0007(10)

O2 0.0509(10) 0.0817(13) 0.0494(10) 0.0020(9) 0.0002(8) 0.0001(9)

O3 0.0929(13) 0.0448(11) 0.0420(9) 0.0018(8) 0.0019(8) 0.0207(9)

N1 0.0544(12) 0.0511(14) 0.0393(10) 0.0037(9) -0.0027(9) 0.0033(10)

N2 0.0608(12) 0.0377(11) 0.0364(10) -0.0015(8) 0.0079(9) -0.0008(10)

N3 0.0611(13) 0.0444(13) 0.0479(12) -0.0040(10) 0.0103(10) -0.0030(11)

C1 0.0504(14) 0.0508(17) 0.0512(14) -0.0031(12) 0.0011(11) 0.0082(12)

C2 0.0574(16) 0.0615(18) 0.0513(15) -0.0113(13) -0.0067(12) -0.0043(13)

C3 0.0542(15) 0.075(2) 0.0393(13) -0.0036(13) -0.0071(11) 0.0000(14)

C4 0.0484(14) 0.0577(17) 0.0369(11) 0.0007(11) -0.0011(10) 0.0016(12)

C5 0.0754(18) 0.074(2) 0.0436(14) 0.0155(14) -0.0134(13) -0.0016(16)

C6 0.091(2) 0.0604(19) 0.0562(16) 0.0208(14) -0.0119(15) -0.0061(16)

C7 0.084(2) 0.0512(17) 0.0523(15) 0.0027(13) -0.0073(14) -0.0059(14)

C8 0.0682(17) 0.0525(16) 0.0361(12) -0.0005(11) -0.0083(11) 0.0005(13)

C9 0.0437(13) 0.0497(15) 0.0340(11) -0.0001(10) 0.0043(9) 0.0066(11)

C10 0.0416(13) 0.0501(15) 0.0347(11) -0.0019(10) 0.0021(9) 0.0055(11)

C11 0.0495(14) 0.0483(16) 0.0401(12) -0.0002(11) 0.0032(10) 0.0033(11)

C12 0.0436(13) 0.0490(15) 0.0377(12) 0.0057(11) 0.0051(10) 0.0073(11)

C13 0.0730(17) 0.0409(15) 0.0457(13) 0.0098(11) -0.0040(12) -0.0028(13)

C14 0.0713(17) 0.0461(16) 0.0439(13) 0.0012(11) -0.0059(12) -0.0030(13)

C15 0.0488(13) 0.0446(15) 0.0336(11) 0.0010(10) 0.0047(10) 0.0079(11)

C16 0.0659(16) 0.0398(14) 0.0422(12) 0.0031(11) 0.0018(11) 0.0056(12)

C17 0.0598(16) 0.0470(16) 0.0424(13) -0.0051(11) -0.0032(11) 0.0016(12)

C18 0.0574(15) 0.0375(13) 0.0340(11) 0.0023(10) 0.0000(10) 0.0030(12)

C19 0.0589(17) 0.0621(19) 0.081(2) -0.0010(16) 0.0080(14) -0.0108(14)

C20 0.0615(18) 0.0620(19) 0.0628(16) 0.0005(14) 0.0160(13) 0.0012(15)

_geom_special_details
;
All esds (except the esd in the dihedral angle between two l.s. planes)
are estimated using the full covariance matrix. The cell esds are taken
into account individually in the estimation of esds in distances, angles
and torsion angles; correlations between esds in cell parameters are only
used when they are defined by crystal symmetry. An approximate (isotropic)
treatment of cell esds is used for estimating esds involving l.s. planes.
;

loop_
_geom_bond_atom_site_label_1
_geom_bond_atom_site_label_2
_geom_bond_distance
_geom_bond_site_symmetry_2
_geom_bond_publ_flag
S1 O2 1.4315(17) . ?
S1 O3 1.4344(17) . ?
S1 N2 1.6056(18) . ?
S1 C15 1.762(2) . ?
S2 C19 1.719(3) . ?
S2 C18 1.724(2) . ?
O1 C1 1.269(3) . ?
N1 C11 1.325(3) . ?
N1 C12 1.402(3) . ?
N1 H1 0.98(2) . ?
N2 C18 1.325(3) . ?
N3 C18 1.338(3) . ?
N3 C20 1.370(3) . ?
N3 H3A 0.89(2) . ?
C1 C2 1.431(3) . ?
C1 C10 1.434(3) . ?

C2 C3 1.331(3) . ?

C2 H2 0.9300 . ?

C3 C4 1.433(3) . ?

C3 H3 0.9300 . ?

C4 C5 1.396(3) . ?

C4 C9 1.413(3) . ?

C5 C6 1.362(4) . ?

C5 H5 0.9300 . ?

C6 C7 1.379(3) . ?

C6 H6 0.9300 . ?

C7 C8 1.362(3) . ?

C7 H7 0.9300 . ?

C8 C9 1.394(3) . ?

C8 H8 0.9300 . ?

C9 C10 1.453(3) . ?

C10 C11 1.388(3) . ?

C11 H11 0.9300 . ?

C12 C17 1.375(3) . ?

C12 C13 1.376(3) . ?

C13 C14 1.374(3) . ?

C13 H13 0.9300 . ?

C14 C15 1.373(3) . ?

C14 H14 0.9300 . ?

C15 C16 1.375(3) . ?

C16 C17 1.376(3) . ?

C16 H16 0.9300 . ?

C17 H17 0.9300 . ?

C19 C20 1.315(3) . ?

C19 H19 0.9300 . ?

C20 H20 0.9300 . ?

loop_

_geom_angle_atom_site_label_1

_geom_angle_atom_site_label_2

_geom_angle_atom_site_label_3

_geom_angle

_geom_angle_site_symmetry_1

_geom_angle_site_symmetry_3

_geom_angle_publ_flag

O2 S1 O3 118.78(11) . . ?

O2 S1 N2 104.17(10) . . ?

O3 S1 N2 111.73(10) . . ?

O2 S1 C15 108.12(11) . . ?

O3 S1 C15 107.28(10) . . ?

N2 S1 C15 106.06(10) . . ?

C19 S2 C18 90.71(13) . . ?

C11 N1 C12 127.3(2) . . ?

C11 N1 H1 114.3(14) . . ?

C12 N1 H1 118.3(14) . . ?

C18 N2 S1 121.50(16) . . ?

C18 N3 C20 115.5(2) . . ?

C18 N3 H3A 121.7(16) . . ?

C20 N3 H3A 122.8(16) . . ?

O1 C1 C2 118.8(2) . . ?

O1 C1 C10 122.8(2) . . ?

C2 C1 C10 118.4(2) . . ?

C3 C2 C1 121.7(2) . . ?

C3 C2 H2 119.2 . . ?

C1 C2 H2 119.2 . . ?

C2 C3 C4 122.3(2) . . ?

C2 C3 H3 118.9 . . ?

C4 C3 H3 118.9 . . ?

C5 C4 C9 119.8(2) . . ?

C5 C4 C3 121.2(2) . . ?

C9 C4 C3 119.0(2) . . ?

C6 C5 C4 121.0(2) . . ?

C6 C5 H5 119.5 . . ?

C4 C5 H5 119.5 . . ?

C5 C6 C7 119.4(2) . . ?

C5 C6 H6 120.3 . . ?

C7 C6 H6 120.3 . . ?

C8 C7 C6 121.0(2) . . ?

C8 C7 H7 119.5 . . ?

C6 C7 H7 119.5 . . ?

C7 C8 C9 121.5(2) . . ?

C7 C8 H8 119.3 . . ?

C9 C8 H8 119.3 . . ?

C8 C9 C4 117.4(2) . . ?

C8 C9 C10 123.51(19) . . ?

C4 C9 C10 119.1(2) . . ?

C11 C10 C1 118.8(2) . . ?

C11 C10 C9 121.6(2) . . ?

C1 C10 C9 119.55(19) . . ?

N1 C11 C10 122.8(2) . . ?

N1 C11 H11 118.6 . . ?

C10 C11 H11 118.6 . . ?

C17 C12 C13 119.3(2) . . ?

C17 C12 N1 116.8(2) . . ?

C13 C12 N1 123.8(2) . . ?

C14 C13 C12 119.8(2) . . ?

C14 C13 H13 120.1 . . ?

C12 C13 H13 120.1 . . ?

C15 C14 C13 120.6(2) . . ?

C15 C14 H14 119.7 . . ?

C13 C14 H14 119.7 . . ?

C14 C15 C16 119.9(2) . . ?

C14 C15 S1 119.37(17) . . ?

C16 C15 S1 120.70(18) . . ?

C15 C16 C17 119.3(2) . . ?

C15 C16 H16 120.3 . . ?

C17 C16 H16 120.3 . . ?

C12 C17 C16 121.0(2) . . ?

C12 C17 H17 119.5 . . ?

C16 C17 H17 119.5 . . ?

N2 C18 N3 119.9(2) . . ?

N2 C18 S2 130.88(18) . . ?

N3 C18 S2 109.21(18) . . ?

C20 C19 S2 112.0(2) . . ?

C20 C19 H19 124.0 . . ?

S2 C19 H19 124.0 . . ?

C19 C20 N3 112.5(2) . . ?

C19 C20 H20 123.7 . . ?

N3 C20 H20 123.7 . . ?

loop_

_geom_torsion_atom_site_label_1

_geom_torsion_atom_site_label_2

_geom_torsion_atom_site_label_3

_geom_torsion_atom_site_label_4

_geom_torsion

_geom_torsion_site_symmetry_1

_geom_torsion_site_symmetry_2

_geom_torsion_site_symmetry_3

_geom_torsion_site_symmetry_4

_geom_torsion_publ_flag

O2 S1 N2 C18 -165.13(17) . . . . ?

O3 S1 N2 C18 -35.7(2) . . . . ?

C15 S1 N2 C18 80.88(19) . . . . ?

O1 C1 C2 C3 179.8(2) . . . . ?

C10 C1 C2 C3 -0.4(4) . . . . ?

C1 C2 C3 C4 0.5(4) . . . . ?

C2 C3 C4 C5 178.1(3) . . . . ?

C2 C3 C4 C9 0.5(4) . . . . ?

C9 C4 C5 C6 0.1(4) . . . . ?

C3 C4 C5 C6 -177.5(3) . . . . ?

C4 C5 C6 C7 0.5(4) . . . . ?

C5 C6 C7 C8 -0.6(4) . . . . ?

C6 C7 C8 C9 0.1(4) . . . . ?

C7 C8 C9 C4 0.5(4) . . . . ?

C7 C8 C9 C10 178.8(2) . . . . ?

C5 C4 C9 C8 -0.6(3) . . . . ?

C3 C4 C9 C8 177.1(2) . . . . ?

C5 C4 C9 C10 -179.0(2) . . . . ?

C3 C4 C9 C10 -1.4(3) . . . . ?

O1 C1 C10 C11 -0.9(3) . . . . ?

C2 C1 C10 C11 179.3(2) . . . . ?

O1 C1 C10 C9 179.3(2) . . . . ?

C2 C1 C10 C9 -0.5(3) . . . . ?

C8 C9 C10 C11 3.3(3) . . . . ?

C4 C9 C10 C11 -178.4(2) . . . . ?

C8 C9 C10 C1 -176.9(2) . . . . ?

C4 C9 C10 C1 1.4(3) . . . . ?

C12 N1 C11 C10 -179.7(2) . . . . ?

C1 C10 C11 N1 2.2(3) . . . . ?

C9 C10 C11 N1 -178.0(2) . . . . ?

C11 N1 C12 C17 -179.6(2) . . . . ?

C11 N1 C12 C13 0.6(4) . . . . ?

C17 C12 C13 C14 0.5(4) . . . . ?

N1 C12 C13 C14 -179.7(2) . . . . ?

C12 C13 C14 C15 -0.7(4) . . . . ?

C13 C14 C15 C16 1.0(4) . . . . ?

C13 C14 C15 S1 179.64(19) . . . . ?

O2 S1 C15 C14 -40.8(2) . . . . ?

O3 S1 C15 C14 -169.98(18) . . . . ?

N2 S1 C15 C14 70.5(2) . . . . ?

O2 S1 C15 C16 137.92(19) . . . . ?

O3 S1 C15 C16 8.7(2) . . . . ?

N2 S1 C15 C16 -110.84(19) . . . . ?

C14 C15 C16 C17 -0.9(3) . . . . ?

S1 C15 C16 C17 -179.56(18) . . . . ?

C13 C12 C17 C16 -0.4(3) . . . . ?

N1 C12 C17 C16 179.7(2) . . . . ?

C15 C16 C17 C12 0.6(3) . . . . ?

S1 N2 C18 N3 178.04(16) . . . . ?

S1 N2 C18 S2 -0.2(3) . . . . ?

C20 N3 C18 N2 180.0(2) . . . . ?

C20 N3 C18 S2 -1.4(3) . . . . ?

C19 S2 C18 N2 179.9(2) . . . . ?

C19 S2 C18 N3 1.55(18) . . . . ?

C18 S2 C19 C20 -1.4(2) . . . . ?

S2 C19 C20 N3 0.9(3) . . . . ?

C18 N3 C20 C19 0.3(3) . . . . ?

loop_

_geom_hbond_atom_site_label_D

_geom_hbond_atom_site_label_H

_geom_hbond_atom_site_label_A

_geom_hbond_distance_DH

_geom_hbond_distance_HA

_geom_hbond_distance_DA

_geom_hbond_angle_DHA

_geom_hbond_site_symmetry_A

_geom_hbond_publ_flag

N1 H1 O1 0.98(2) 1.72(2) 2.530(3) 138(2) . yes

N3 H3A S1 0.89(2) 2.89(3) 3.746(2) 163(2) 3_666 yes

N3 H3A O2 0.89(2) 2.50(2) 3.185(3) 134(2) 3_666 yes

N3 H3A N2 0.89(2) 2.07(2) 2.922(3) 161(2) 3_666 yes

C11 H11 O3 0.93 2.64 3.521(3) 157.4 2_655 yes

C13 H13 O3 0.93 2.58 3.463(3) 158.7 2_655 yes

_refine_diff_density_max 0.260

_refine_diff_density_min -0.271

_refine_diff_density_rms 0.043

_shelx_res_file

;

TITL NAPSULY_0m in P2(1)/c RED PRISM

shelx.res

created by SHELXL-2019/2 at 12:42:26 on 18-Feb-2023

CELL 0.71073 7.8389 13.8151 17.1980 90.000 94.032 90.000

ZERR 4.00 0.0008 0.0016 0.0021 0.000 0.004 0.000

LATT 1

SYMM -x, y+1/2, -z+1/2

SFAC C H N O S

UNIT 80 60 12 12 8

OMIT -3 54

L.S. 40

ACTA

BOND $H

FMAP 2

PLAN 10

CONF

HTAB

TEMP 23.000

SIZE 0.32 0.28 0.23

HTAB N1 O1

EQIV $1 -x+1, -y+1, -z+1

HTAB N3 S1_$1

HTAB N3 O2_$1

HTAB N3 N2_$1

```
EQIV $2 -x+1, y+1/2, -z+1/2
HTAB C11 O3_$2
HTAB C13 O3_$2
OMIT 0   1   1
MPLA 6 N2 C18 C19 C20 S2 N3
MPLA 12 C1 C2 C3 C4 C5 C6 C7 C8 C9 C10 C11 O1
MPLA 7 N1 C12 C13 C14 C15 C16 C17
MPLA 3 S1 O2 O3
MPLA 6 N2 C18 C19 C20 S2 N3
MPLA 7 N1 C12 C13 C14 C15 C16 C17
MPLA 10 C12 C13 C14 C15 C16 C17 S1 O2 O3 N2
MPLA 5 C18 C19 C20 S2 N3
WGHT    0.042900    0.313200
FVAR       0.09598
S1    5    0.628057    0.373793    0.359833    11.00000    0.06087    0.04754 =
         0.03397    0.00184    0.00113    0.00970
S2    5    0.270187    0.276639    0.412422    11.00000    0.07069    0.05016 =
         0.06133   -0.01180    0.00315   -0.01084
O1    4    0.121683    0.415423   -0.065008    11.00000    0.08385    0.04670 =
         0.07113   -0.00012   -0.00997    0.00069
O2    4    0.788859    0.422998    0.369546    11.00000    0.05088    0.08175 =
         0.04944    0.00204    0.00020    0.00012
O3    4    0.625926    0.270120    0.364266    11.00000    0.09287    0.04482 =
         0.04200    0.00183    0.00191    0.02066
N1    3    0.300087    0.481441    0.051420    11.00000    0.05439    0.05107 =
         0.03935    0.00374   -0.00274    0.00327
H1    2    0.238428    0.429667    0.022870    11.00000   -1.20000
N2    3    0.515331    0.422184    0.423883    11.00000    0.06085    0.03769 =
         0.03636   -0.00150    0.00791   -0.00083
N3    3    0.283740    0.422420    0.499368    11.00000    0.06113    0.04441 =
         0.04787   -0.00396    0.01028   -0.00303
H3A   2    0.321545    0.474981    0.524945    11.00000   -1.20000
C1    1    0.123148    0.499229   -0.095202    11.00000    0.05042    0.05084 =
```

```
      0.05121   -0.00314   0.00113   0.00818
C2   1   0.036279   0.514085   -0.170260   11.00000   0.05739   0.06149 =
      0.05134   -0.01130   -0.00670   -0.00432
AFIX  43
H2   2   -0.019101   0.462165   -0.195502   11.00000   -1.20000
AFIX   0
C3   1   0.032738   0.600287   -0.204843   11.00000   0.05419   0.07498 =
      0.03926   -0.00359   -0.00714   -0.00005
AFIX  43
H3   2   -0.025917   0.606851   -0.253469   11.00000   -1.20000
AFIX   0
C4   1   0.116162   0.683350   -0.169763   11.00000   0.04839   0.05775 =
      0.03686   0.00069   -0.00107   0.00162
C5   1   0.114817   0.772307   -0.208268   11.00000   0.07536   0.07401 =
      0.04361   0.01547   -0.01344   -0.00161
AFIX  43
H5   2   0.055085   0.778511   -0.256678   11.00000   -1.20000
AFIX   0
C6   1   0.199669   0.850179   -0.176016   11.00000   0.09102   0.06040 =
      0.05615   0.02085   -0.01190   -0.00611
AFIX  43
H6   2   0.197231   0.909269   -0.201978   11.00000   -1.20000
AFIX   0
C7   1   0.289205   0.840560   -0.104515   11.00000   0.08374   0.05122 =
      0.05233   0.00275   -0.00733   -0.00587
AFIX  43
H7   2   0.348124   0.893463   -0.082612   11.00000   -1.20000
AFIX   0
C8   1   0.292632   0.754694   -0.065476   11.00000   0.06816   0.05254 =
      0.03611   -0.00046   -0.00834   0.00051
AFIX  43
H8   2   0.353845   0.750163   -0.017294   11.00000   -1.20000
AFIX   0
```

```
C9    1    0.206431    0.673458   -0.096136    11.00000    0.04371    0.04969 =
        0.03400   -0.00011    0.00425    0.00660
C10   1    0.208811    0.579934   -0.057408    11.00000    0.04164    0.05011 =
        0.03465   -0.00191    0.00207    0.00546
C11   1    0.291866    0.566535    0.015789    11.00000    0.04949    0.04829 =
        0.04010   -0.00015    0.00315    0.00327
AFIX  43
H11   2    0.343968    0.619554    0.040893    11.00000   -1.20000
AFIX   0
C12   1    0.379533    0.460351    0.125034    11.00000    0.04356    0.04903 =
        0.03774    0.00568    0.00510    0.00730
C13   1    0.462201    0.528157    0.172658    11.00000    0.07304    0.04085 =
        0.04574    0.00979   -0.00401   -0.00278
AFIX  43
H13   2    0.467179    0.592349    0.156691    11.00000   -1.20000
AFIX   0
C14   1    0.537379    0.500798    0.243863    11.00000    0.07131    0.04609 =
        0.04386    0.00120   -0.00593   -0.00302
AFIX  43
H14   2    0.594324    0.546593    0.275698    11.00000   -1.20000
AFIX   0
C15   1    0.529174    0.406454    0.268431    11.00000    0.04883    0.04457 =
        0.03357    0.00105    0.00473    0.00795
C16   1    0.447293    0.338205    0.221093    11.00000    0.06591    0.03984 =
        0.04224    0.00310    0.00184    0.00558
AFIX  43
H16   2    0.442650    0.274010    0.237083    11.00000   -1.20000
AFIX   0
C17   1    0.372273    0.365881    0.149795    11.00000    0.05983    0.04701 =
        0.04239   -0.00511   -0.00319    0.00163
AFIX  43
H17   2    0.315809    0.319999    0.117875    11.00000   -1.20000
AFIX   0
```

```
C18   1    0.371849    0.381591    0.444147    11.00000    0.05738    0.03751 =
        0.03402    0.00235   -0.00002    0.00302
C19   1    0.110644    0.296188    0.474215    11.00000    0.05894    0.06214 =
        0.08122   -0.00098    0.00802   -0.01077
AFIX  43
H19   2    0.016556    0.255785    0.477583    11.00000   -1.20000
AFIX   0
C20   1    0.137298    0.375007    0.516166    11.00000    0.06147    0.06202 =
        0.06277    0.00052    0.01600    0.00118
AFIX  43
H20   2    0.064756    0.396229    0.553157    11.00000   -1.20000
AFIX   0
HKLF 4 1 1 0 0 0 1 0 0 0 1




REM  NAPSULY_0m in P2(1)/c RED PRISM
REM wR2 = 0.1087, GooF = S = 1.018, Restrained GooF = 1.018 for all data
REM R1 = 0.0437 for 2548 Fo > 4sig(Fo) and 0.0852 for all 4040 data
REM 259 parameters refined using 0 restraints

END
```

CIF of ACPIN without hkl data

```
data_global

#==================================================================
# PROCESSING SUMMARY (IUCr Office Use only)

_publ_contact_author
;
```

Muhammad Nawaz Tahir

University of Sargodha

Department of Physics

Sargodha

Pakistan

;

_publ_contact_author_phone '0092 48 92 30 914'

_publ_contact_author_fax '0092 48 32 22 121'

_publ_contact_author_email 'dmntahir_uos@yahoo.com'

_publ_requested_journal ''

_journal_date_recd_electronic ?

_journal_date_to_coeditor ?

_journal_date_from_coeditor ?

_journal_date_accepted ?

_journal_date_printers_first ?

_journal_date_printers_final ?

_journal_date_proofs_out ?

_journal_date_proofs_in ?

_journal_coeditor_name ?

_journal_coeditor_code ?

_journal_paper_category ?

_journal_coeditor_notes

;

;

_journal_techeditor_code ?

_iucr_compatibility_tag ?

_journal_techeditor_notes

;

;

```
_journal_coden_ASTM              ?
_journal_name_full               ?
_journal_year                    ?
_journal_volume                  ?
_journal_issue                   ?
_journal_page_first              ?
_journal_page_last               ?

_journal_suppl_publ_number       ?
_journal_suppl_publ_pages        ?
#==============================================================

 loop_
_publ_author_name
_publ_author_address

'Muhammad Ashfaq'
;
Department of Physics
University of Sargodha
Sargodha
Pakistan
orcid id: 0000-0001-6663-8777
;

'Muhammad Nawaz Tahir'
;
Department of Physics
University of Sargodha
Sargodha
Pakistan
orcid id: 0000-0002-6815-9806
```

;

'Akbar Ali'
;
Department of Chemistry,
Government College University
Faisalabad,
Pakistan.
;

'Abida Naseem Malik'
;
Department of Physics
University of Sargodha
Sargodha
Pakistan
;
'Aleksey Kuznetsov'
;
Departamento de Quimica,
Campus Santiago Vitacura,
Universidad Tecnica Federico Santa Maria,
Av. Santa Maria 6400 Vitacura,
Postal code: 7660251,
Chile
;

_audit_creation_date ?
_audit_creation_method ?
#==

TEXT

_publ_section_title

;

(E)-1-(((4-amino-5-(4-chlorophenyl)-6-ethylpyrimidin-2-yl)iminio)methyl)

naphthalen-2-olate

;

_publ_section_abstract

;

;

_publ_section_comment

;

;

_publ_section_exptl_refinement

;

;

_publ_section_exptl_prep

;

;

_publ_section_related_literature

;

;

_publ_section_references

;

Bruker (2007). <i>SADABS</i>. Bruker AXS Inc., Madison, Wisconsin, USA.

Bruker (2007). <i>APEX2</i> and <i>SAINT</i>. Bruker AXS Inc.,
Madison, Wisconsin, USA.

Farrugia, L. J. (1997). <i>J. Appl. Cryst.</i> 30, 565.

Farrugia, L. J. (2012). <i>J. Appl. Cryst.</i> 45, 849--854.

Sheldrick, G. M. (2008). <i>Acta Cryst.</i> A64, 112--122.

Sheldrick, G. M. (2015). <i>Acta Cryst.</i> C71, 3--8.

Spek, A. L. (2009). <i>Acta Cryst.</i> D65, 148--155.

;

_publ_section_figure_captions

;

;

_publ_section_acknowledgements

;

;

data_pymtvan_twnew

_audit_creation_method 'SHELXL-2019/2'

_shelx_SHELXL_version_number '2019/2'

_chemical_name_common ?

_chemical_absolute_configuration '.'

_chemical_name_systematic

;

(E)-1-(((4-amino-5-(4-chlorophenyl)-6-ethylpyrimidin-2-yl)iminio)methyl)

naphthalen-2-olate

;

_chemical_formula_moiety 'C23 H19 Cl N4 O'

_chemical_formula_sum 'C23 H19 Cl N4 O'

_chemical_formula_iupac 'C23 H19 Cl N4 O'

_chemical_formula_weight 402.87

loop_

_atom_type_symbol

_atom_type_description

_atom_type_scat_dispersion_real

_atom_type_scat_dispersion_imag

_atom_type_scat_source

'C' 'C' 0.0033 0.0016

'International Tables Vol C Tables 4.2.6.8 and 6.1.1.4'

'H' 'H' 0.0000 0.0000

'International Tables Vol C Tables 4.2.6.8 and 6.1.1.4'

'N' 'N' 0.0061 0.0033

'International Tables Vol C Tables 4.2.6.8 and 6.1.1.4'

'O' 'O' 0.0106 0.0060

'International Tables Vol C Tables 4.2.6.8 and 6.1.1.4'

'Cl' 'Cl' 0.1484 0.1585

'International Tables Vol C Tables 4.2.6.8 and 6.1.1.4'

_space_group_crystal_system triclinic

_space_group_IT_number 2

_space_group_name_H-M_alt 'P -1'

_space_group_name_Hall '-P 1'

_shelx_space_group_comment

;

The symmetry employed for this shelxl refinement is uniquely defined

by the following loop, which should always be used as a source of

symmetry information in preference to the above space-group names.

They are only intended as comments.

;

```
loop_
 _space_group_symop_operation_xyz
 'x, y, z'
 '-x, -y, -z'

_cell_length_a                    4.5436(6)
_cell_length_b                    12.5255(16)
_cell_length_c                    17.673(2)
_cell_angle_alpha                 88.308(8)
_cell_angle_beta                  89.195(8)
_cell_angle_gamma                 82.611(9)
_cell_volume                      996.9(2)
_cell_formula_units_Z             2
_cell_measurement_reflns_used     2397
_cell_measurement_theta_min       1.640
_cell_measurement_theta_max       27.448
_cell_measurement_temperature     296(2)
_exptl_crystal_description        needle
_exptl_crystal_colour             'yellow'
_exptl_crystal_size_max     0.42
_exptl_crystal_size_mid     0.24
_exptl_crystal_size_min     0.20
_exptl_crystal_density_diffrn     1.324
_exptl_crystal_density_meas     ?
_exptl_crystal_density_method     'not measured'
_exptl_crystal_F_000              420
_exptl_absorpt_coefficient_mu     0.214
_exptl_absorpt_correction_type     multi-scan
_exptl_absorpt_process_details     '(SADABS; Bruker, 2007)'
_exptl_absorpt_correction_T_min   0.869
_exptl_absorpt_correction_T_max   0.894
_exptl_special_details
;
```

;

_diffrn_ambient_temperature 296(2)
_diffrn_radiation_type MoK\a
_diffrn_radiation_wavelength 0.71073
_diffrn_radiation_source 'fine-focus sealed tube'
_diffrn_radiation_monochromator graphite
_diffrn_measurement_device_type 'Bruker Kappa APEXII CCD'
_diffrn_measurement_method \w
_diffrn_detector_area_resol_mean 7.710
_diffrn_reflns_number 4351
_diffrn_reflns_av_unetI/netI 0.0554
_diffrn_reflns_av_R_equivalents ?
_diffrn_reflns_limit_h_min -5
_diffrn_reflns_limit_h_max 5
_diffrn_reflns_limit_k_min -16
_diffrn_reflns_limit_k_max 16
_diffrn_reflns_limit_l_min -1
_diffrn_reflns_limit_l_max 22
_diffrn_reflns_theta_min 1.640
_diffrn_reflns_theta_max 27.448
_diffrn_reflns_theta_full 25.242
_diffrn_measured_fraction_theta_max 0.958
_diffrn_measured_fraction_theta_full 0.966
_diffrn_reflns_Laue_measured_fraction_max 0.958
_diffrn_reflns_Laue_measured_fraction_full 0.966
_diffrn_reflns_point_group_measured_fraction_max 0.958
_diffrn_reflns_point_group_measured_fraction_full 0.966
_diffrn_standards_number 0
_diffrn_standards_interval_count ?
_diffrn_standards_interval_time ?
_diffrn_standards_decay_% ?

_refine_special_details

;

Refinement of <i>F</i>^2^ against ALL reflections. The weighted <i>R</i>-factor

<i>wR</i> and goodness of fit <i>S</i> are based on <i>F</i>^2^, conventional

<i>R</i>-factors <i>R</i> are based on <i>F</i>, with <i>F</i> set to zero for

negative <i>F</i>^2^. The threshold expression of <i>F</i>^2^ >

\s(<i>F</i>^2^) is used only for calculating <i>R</i>-factors(gt) <i>etc</i>.

and is not relevant to the choice of reflections for refinement.

<i>R</i>-factors based on <i>F</i>^2^ are statistically about twice as large

as those based on <i>F</i>, and <i>R</i>- factors based on ALL data will be

even larger.

;

_reflns_number_total 4351

_reflns_number_gt 2397

_reflns_threshold_expression I>2\s(I)

_refine_ls_structure_factor_coef Fsqd

_refine_ls_matrix_type full

_refine_ls_R_factor_all 0.1311

_refine_ls_R_factor_gt 0.0763

_refine_ls_wR_factor_ref 0.2358

_refine_ls_wR_factor_gt 0.2131

_refine_ls_goodness_of_fit_ref 1.139

_refine_ls_restrained_S_all 1.139

_refine_ls_number_reflns 4351

_refine_ls_number_parameters 264

_refine_ls_number_restraints 0

_refine_ls_extinction_method none

_refine_ls_extinction_coef .

_refine_ls_hydrogen_treatment constr

_refine_ls_weighting_scheme calc

_refine_ls_weighting_details

'w=1/[\s^2^(Fo^2^)+(0.0757P)^2^+0.7915P] where P=(Fo^2^+2Fc^2^)/3'

```
_atom_sites_solution_hydrogens     geom
_atom_sites_solution_primary     direct
_atom_sites_solution_secondary     difmap
_refine_ls_shift/su_max      0.000
_refine_ls_shift/su_mean      0.000
_computing_data_collection     'APEX2 (Bruker, 2007)'
_computing_cell_refinement     'SAINT (Bruker, 2007)'
_computing_data_reduction      'SAINT (Bruker, 2007)'
_computing_structure_solution     'SHELXT2014 (Sheldrick, 2008)'
_computing_structure_refinement   'SHELXL-2019/2  (Sheldrick, 2015)'
_computing_molecular_graphics
'ORTEP-3 for Windows (Farrugia, 2012) and PLATON (Spek, 2009)'
_computing_publication_material
;
WinGX (Farrugia, 2012) and PLATON (Spek, 2009)
;


loop_
 _atom_site_label
 _atom_site_type_symbol
 _atom_site_fract_x
 _atom_site_fract_y
 _atom_site_fract_z
 _atom_site_U_iso_or_equiv
 _atom_site_adp_type
 _atom_site_occupancy
 _atom_site_site_symmetry_order
 _atom_site_calc_flag
 _atom_site_refinement_flags_posn
 _atom_site_refinement_flags_adp
 _atom_site_refinement_flags_occupancy
 _atom_site_disorder_assembly
```

_atom_site_disorder_group

Cl1 Cl 0.8437(5) 0.66072(16) -0.44259(7) 0.1244(8) Uani 1 1 d . . . . .

O1 O 1.5052(8) 0.6311(2) 0.22359(17) 0.0647(9) Uani 1 1 d . . . . .

N1 N 1.1691(8) 0.7121(2) 0.11607(17) 0.0506(9) Uani 1 1 d . . . . .

H1 H 1.316681 0.667310 0.131723 0.061 Uiso 1 1 calc R U . . .

N2 N 1.2336(8) 0.6206(3) 0.00662(17) 0.0515(9) Uani 1 1 d . . . . .

N3 N 1.3058(9) 0.5222(3) -0.09936(18) 0.0620(10) Uani 1 1 d . . . . .

H3A H 1.422638 0.476107 -0.073272 0.074 Uiso 1 1 calc R U . . .

H3B H 1.275403 0.511139 -0.146252 0.074 Uiso 1 1 calc R U . . .

N4 N 0.8944(8) 0.7821(2) 0.01133(18) 0.0490(8) Uani 1 1 d . . . . .

C1 C 1.3595(10) 0.6975(3) 0.2669(2) 0.0519(10) Uani 1 1 d . . . . .

C2 C 1.4275(11) 0.6933(4) 0.3474(2) 0.0606(12) Uani 1 1 d . . . . .

H2 H 1.576803 0.641685 0.365800 0.073 Uiso 1 1 calc R U . . .

C3 C 1.2799(11) 0.7618(4) 0.3950(2) 0.0606(12) Uani 1 1 d . . . . .

H3 H 1.330356 0.756311 0.445923 0.073 Uiso 1 1 calc R U . . .

C4 C 1.0470(10) 0.8437(3) 0.3713(2) 0.0530(11) Uani 1 1 d . . . . .

C5 C 0.8982(12) 0.9153(4) 0.4231(2) 0.0629(13) Uani 1 1 d . . . . .

H5 H 0.948896 0.908272 0.473926 0.075 Uiso 1 1 calc R U . . .

C6 C 0.6814(12) 0.9948(4) 0.4005(3) 0.0683(13) Uani 1 1 d . . . . .

H6 H 0.587866 1.042410 0.435310 0.082 Uiso 1 1 calc R U . . .

C7 C 0.6019(11) 1.0036(4) 0.3247(2) 0.0625(12) Uani 1 1 d . . . . .

H7 H 0.451165 1.056642 0.308993 0.075 Uiso 1 1 calc R U . . .

C8 C 0.7432(10) 0.9353(3) 0.2731(2) 0.0554(11) Uani 1 1 d . . . . .

H8 H 0.687906 0.943491 0.222608 0.067 Uiso 1 1 calc R U . . .

C9 C 0.9688(10) 0.8531(3) 0.2940(2) 0.0455(9) Uani 1 1 d . . . . .

C10 C 1.1248(9) 0.7779(3) 0.2408(2) 0.0441(9) Uani 1 1 d . . . . .

C11 C 1.0389(10) 0.7792(3) 0.1662(2) 0.0479(10) Uani 1 1 d . . . . .

H11 H 0.881995 0.829698 0.150473 0.057 Uiso 1 1 calc R U . . .

C12 C 1.0917(10) 0.7062(3) 0.0402(2) 0.0459(9) Uani 1 1 d . . . . .

C13 C 1.1704(10) 0.6115(3) -0.0668(2) 0.0488(10) Uani 1 1 d . . . . .

C14 C 0.9780(9) 0.6917(3) -0.1065(2) 0.0434(9) Uani 1 1 d . . . . .

C15 C 0.8397(9) 0.7750(3) -0.0636(2) 0.0448(9) Uani 1 1 d . . . . .

C16 C 0.6198(10) 0.8645(3) -0.0940(2) 0.0551(11) Uani 1 1 d . . . . .

H16A H 0.438920 0.867660 -0.063915 0.066 Uiso 1 1 calc R U . . .

H16B H 0.571511 0.848972 -0.145513 0.066 Uiso 1 1 calc R U . . .

C17 C 0.7319(12) 0.9725(3) -0.0936(3) 0.0760(15) Uani 1 1 d . . . . .

H17A H 0.808426 0.983545 -0.044450 0.114 Uiso 1 1 calc R U . . .

H17B H 0.571904 1.028182 -0.105070 0.114 Uiso 1 1 calc R U . . .

H17C H 0.886726 0.974607 -0.130896 0.114 Uiso 1 1 calc R U . . .

C18 C 0.9410(9) 0.6853(3) -0.1905(2) 0.0461(9) Uani 1 1 d . . . . .

C19 C 1.0302(11) 0.7619(3) -0.2399(2) 0.0570(11) Uani 1 1 d . . . . .

H19 H 1.112091 0.819797 -0.220837 0.068 Uiso 1 1 calc R U . . .

C20 C 1.0012(12) 0.7551(4) -0.3177(2) 0.0683(13) Uani 1 1 d . . . . .

H20 H 1.061097 0.807895 -0.350439 0.082 Uiso 1 1 calc R U . . .

C21 C 0.8825(12) 0.6690(4) -0.3453(2) 0.0665(13) Uani 1 1 d . . . . .

C22 C 0.7958(13) 0.5910(4) -0.2986(3) 0.0749(15) Uani 1 1 d . . . . .

H22 H 0.716106 0.532999 -0.318188 0.090 Uiso 1 1 calc R U . . .

C23 C 0.8277(11) 0.5989(4) -0.2210(2) 0.0635(13) Uani 1 1 d . . . . .

H23 H 0.771632 0.544828 -0.188875 0.076 Uiso 1 1 calc R U . . .

loop_

_atom_site_aniso_label

_atom_site_aniso_U_11

_atom_site_aniso_U_22

_atom_site_aniso_U_33

_atom_site_aniso_U_23

_atom_site_aniso_U_13

_atom_site_aniso_U_12

Cl1 0.189(2) 0.1558(16) 0.0369(7) -0.0017(8) -0.0176(9) -0.0536(14)

O1 0.076(2) 0.0589(18) 0.0561(19) -0.0029(14) -0.0072(16) 0.0057(16)

N1 0.066(2) 0.0479(18) 0.0349(17) -0.0006(14) -0.0026(15) 0.0031(16)

N2 0.070(2) 0.0467(19) 0.0353(17) 0.0002(14) 0.0037(16) 0.0028(17)

N3 0.086(3) 0.058(2) 0.0346(18) -0.0019(15) 0.0001(17) 0.0190(19)

N4 0.058(2) 0.0459(18) 0.0418(18) -0.0051(14) -0.0021(15) 0.0005(16)

C1 0.069(3) 0.049(2) 0.039(2) 0.0005(18) -0.005(2) -0.012(2)

C2 0.072(3) 0.062(3) 0.047(2) 0.007(2) -0.012(2) -0.004(2)

C3 0.079(3) 0.069(3) 0.036(2) 0.004(2) -0.018(2) -0.020(3)

C4 0.067(3) 0.058(2) 0.038(2) -0.0038(18) 0.0008(19) -0.024(2)

C5 0.086(4) 0.074(3) 0.034(2) -0.009(2) 0.001(2) -0.026(3)

C6 0.082(4) 0.071(3) 0.053(3) -0.019(2) 0.015(2) -0.014(3)

C7 0.069(3) 0.065(3) 0.051(3) -0.008(2) 0.005(2) -0.002(2)

C8 0.065(3) 0.061(3) 0.041(2) -0.0016(19) 0.000(2) -0.008(2)

C9 0.056(3) 0.049(2) 0.0337(19) -0.0009(16) 0.0001(17) -0.0170(19)

C10 0.055(3) 0.043(2) 0.0352(19) 0.0019(15) -0.0002(17) -0.0104(18)

C11 0.063(3) 0.040(2) 0.040(2) 0.0009(16) -0.0051(19) -0.0041(18)

C12 0.061(3) 0.045(2) 0.0326(19) 0.0024(16) -0.0010(17) -0.0079(19)

C13 0.063(3) 0.046(2) 0.036(2) -0.0004(16) 0.0069(18) -0.0004(19)

C14 0.051(3) 0.044(2) 0.0357(19) 0.0006(16) -0.0001(17) -0.0093(18)

C15 0.053(3) 0.047(2) 0.0350(19) -0.0024(16) -0.0052(17) -0.0060(18)

C16 0.056(3) 0.060(3) 0.046(2) -0.0112(19) -0.0108(19) 0.006(2)

C17 0.088(4) 0.052(3) 0.085(4) 0.002(2) -0.032(3) 0.007(2)

C18 0.053(3) 0.045(2) 0.039(2) 0.0001(16) -0.0007(17) -0.0042(18)

C19 0.073(3) 0.057(3) 0.044(2) 0.0058(19) -0.009(2) -0.016(2)

C20 0.092(4) 0.069(3) 0.046(2) 0.015(2) -0.004(2) -0.021(3)

C21 0.084(4) 0.082(3) 0.034(2) -0.003(2) -0.004(2) -0.013(3)

C22 0.107(5) 0.079(3) 0.045(3) -0.009(2) -0.006(3) -0.035(3)

C23 0.087(4) 0.063(3) 0.045(2) 0.002(2) 0.004(2) -0.028(2)

_geom_special_details

;

All esds (except the esd in the dihedral angle between two l.s. planes)

are estimated using the full covariance matrix. The cell esds are taken

into account individually in the estimation of esds in distances, angles

and torsion angles; correlations between esds in cell parameters are only

used when they are defined by crystal symmetry. An approximate (isotropic)

treatment of cell esds is used for estimating esds involving l.s. planes.

;

loop_

_geom_bond_atom_site_label_1

_geom_bond_atom_site_label_2

_geom_bond_distance

_geom_bond_site_symmetry_2

_geom_bond_publ_flag

Cl1 C21 1.739(4) . ?

O1 C1 1.263(5) . ?

N1 C11 1.320(5) . ?

N1 C12 1.397(5) . ?

N1 H1 0.8600 . ?

N2 C12 1.330(5) . ?

N2 C13 1.344(5) . ?

N3 C13 1.346(5) . ?

N3 H3A 0.8600 . ?

N3 H3B 0.8600 . ?

N4 C12 1.316(5) . ?

N4 C15 1.358(5) . ?

C1 C10 1.440(6) . ?

C1 C2 1.458(5) . ?

C2 C3 1.332(6) . ?

C2 H2 0.9300 . ?

C3 C4 1.433(6) . ?

C3 H3 0.9300 . ?

C4 C5 1.404(6) . ?

C4 C9 1.416(5) . ?

C5 C6 1.362(7) . ?

C5 H5 0.9300 . ?

C6 C7 1.389(6) . ?

C6 H6 0.9300 . ?

C7 C8 1.366(6) . ?

C7 H7 0.9300 . ?

C8 C9 1.401(6) . ?

C8 H8 0.9300 . ?

C9 C10 1.461(5) . ?

C10 C11 1.379(5) . ?

C11 H11 0.9300 . ?

C13 C14 1.419(5) . ?

C14 C15 1.387(5) . ?

C14 C18 1.501(5) . ?

C15 C16 1.495(5) . ?

C16 C17 1.506(6) . ?

C16 H16A 0.9700 . ?

C16 H16B 0.9700 . ?

C17 H17A 0.9600 . ?

C17 H17B 0.9600 . ?

C17 H17C 0.9600 . ?

C18 C19 1.375(5) . ?

C18 C23 1.383(6) . ?

C19 C20 1.389(6) . ?

C19 H19 0.9300 . ?

C20 C21 1.371(6) . ?

C20 H20 0.9300 . ?

C21 C22 1.354(6) . ?

C22 C23 1.388(6) . ?

C22 H22 0.9300 . ?

C23 H23 0.9300 . ?

loop_

_geom_angle_atom_site_label_1

_geom_angle_atom_site_label_2

_geom_angle_atom_site_label_3

_geom_angle

_geom_angle_site_symmetry_1

_geom_angle_site_symmetry_3

_geom_angle_publ_flag

C11 N1 C12 126.7(4) . . ?

C11 N1 H1 116.7 . . ?

C12 N1 H1 116.7 . . ?

C12 N2 C13 115.6(3) . . ?

C13 N3 H3A 120.0 . . ?

C13 N3 H3B 120.0 . . ?

H3A N3 H3B 120.0 . . ?

C12 N4 C15 115.8(3) . . ?

O1 C1 C10 123.1(3) . . ?

O1 C1 C2 119.3(4) . . ?

C10 C1 C2 117.6(4) . . ?

C3 C2 C1 121.3(4) . . ?

C3 C2 H2 119.3 . . ?

C1 C2 H2 119.3 . . ?

C2 C3 C4 123.0(4) . . ?

C2 C3 H3 118.5 . . ?

C4 C3 H3 118.5 . . ?

C5 C4 C9 119.5(4) . . ?

C5 C4 C3 121.4(4) . . ?

C9 C4 C3 119.1(4) . . ?

C6 C5 C4 121.6(4) . . ?

C6 C5 H5 119.2 . . ?

C4 C5 H5 119.2 . . ?

C5 C6 C7 119.1(4) . . ?

C5 C6 H6 120.5 . . ?

C7 C6 H6 120.5 . . ?

C8 C7 C6 120.6(5) . . ?

C8 C7 H7 119.7 . . ?

C6 C7 H7 119.7 . . ?

C7 C8 C9 122.0(4) . . ?

C7 C8 H8 119.0 . . ?

C9 C8 H8 119.0 . . ?

C8 C9 C4 117.2(4) . . ?

C8 C9 C10 123.9(3) . . ?

C4 C9 C10 119.0(4) . . ?

C11 C10 C1 119.3(4) . . ?

C11 C10 C9 120.5(4) . . ?

C1 C10 C9 120.1(3) . . ?

N1 C11 C10 123.0(4) . . ?

N1 C11 H11 118.5 . . ?

C10 C11 H11 118.5 . . ?

N4 C12 N2 128.5(3) . . ?

N4 C12 N1 118.6(3) . . ?

N2 C12 N1 113.0(3) . . ?

N2 C13 N3 115.0(4) . . ?

N2 C13 C14 121.8(3) . . ?

N3 C13 C14 123.2(3) . . ?

C15 C14 C13 116.1(3) . . ?

C15 C14 C18 123.7(3) . . ?

C13 C14 C18 120.2(3) . . ?

N4 C15 C14 122.1(4) . . ?

N4 C15 C16 113.6(3) . . ?

C14 C15 C16 124.3(3) . . ?

C15 C16 C17 113.0(4) . . ?

C15 C16 H16A 109.0 . . ?

C17 C16 H16A 109.0 . . ?

C15 C16 H16B 109.0 . . ?

C17 C16 H16B 109.0 . . ?

H16A C16 H16B 107.8 . . ?

C16 C17 H17A 109.5 . . ?

C16 C17 H17B 109.5 . . ?

H17A C17 H17B 109.5 . . ?

C16 C17 H17C 109.5 . . ?

H17A C17 H17C 109.5 . . ?

H17B C17 H17C 109.5 . . ?

C19 C18 C23 117.5(4) . . ?

C19 C18 C14 121.6(4) . . ?

C23 C18 C14 120.8(3) . . ?

C18 C19 C20 121.7(4) . . ?

C18 C19 H19 119.2 . . ?

C20 C19 H19 119.2 . . ?

C21 C20 C19 118.7(4) . . ?

C21 C20 H20 120.7 . . ?

C19 C20 H20 120.7 . . ?

C22 C21 C20 121.5(4) . . ?

C22 C21 Cl1 119.7(4) . . ?

C20 C21 Cl1 118.8(4) . . ?

C21 C22 C23 119.0(4) . . ?

C21 C22 H22 120.5 . . ?

C23 C22 H22 120.5 . . ?

C18 C23 C22 121.6(4) . . ?

C18 C23 H23 119.2 . . ?

C22 C23 H23 119.2 . . ?

loop_

_geom_hbond_atom_site_label_D

_geom_hbond_atom_site_label_H

_geom_hbond_atom_site_label_A

_geom_hbond_distance_DH

_geom_hbond_distance_HA

_geom_hbond_distance_DA

_geom_hbond_angle_DHA

_geom_hbond_site_symmetry_A

_geom_hbond_publ_flag

N1 H1 O1 0.86 1.86 2.557(4) 136.6 . yes

N3 H3A N2 0.86 2.19 3.037(5) 170.8 2_865 yes

N3 H3B O1 0.86 2.38 3.003(5) 129.2 2_865 yes

_refine_diff_density_max 0.317

_refine_diff_density_min -0.275

_refine_diff_density_rms 0.079

_shelx_res_file

;

TITL PYMTVAN_0m in P-1 YELLOW NEEDLE

shelx.res

created by SHELXL-2019/2 at 14:15:37 on 18-Feb-2023

CELL 0.71073 4.5436 12.5255 17.6725 88.308 89.195 82.611

ZERR 2.00 0.0006 0.0016 0.0022 0.008 0.008 0.009

LATT 1

SFAC C H N O Cl

UNIT 46 38 8 2 2

L.S. 20

ACTA

LIST 4

BOND $H

HTAB

HTAB N1 O1

EQIV $1 -x+3, -y+1, -z

HTAB N3 N2_$1

HTAB N3 O1_$1

FMAP 2

PLAN 10

SIZE 0.40 0.24 0.20

TEMP 23.000

MPLA 9 C12 N2 C13 C14 C15 N4 C16 C17 N3

MPLA 7 C18 C19 C20 C21 C22 C23 Cl1

MPLA 11 C1 C2 C3 C4 C5 C6 C7 C8 C9 C10 C11 C12 O1

MPLA 9 C12 N2 C13 C14 C15 N4 C16 C17 N3

WGHT 0.075700 0.791500

BASF 0.18748

FVAR 10.25644

```
CL1   5    0.843720    0.660723   -0.442590    11.00000    0.18910    0.15579 =
        0.03694   -0.00168   -0.01765   -0.05358
O1    4    1.505207    0.631141    0.223588    11.00000    0.07565    0.05889 =
        0.05611   -0.00290   -0.00718    0.00569
N1    3    1.169096    0.712051    0.116073    11.00000    0.06622    0.04789 =
        0.03492   -0.00057   -0.00264    0.00309
AFIX  43
H1    2    1.316681    0.667310    0.131723    11.00000   -1.20000
AFIX   0
N2    3    1.233611    0.620577    0.006622    11.00000    0.06953    0.04674 =
        0.03526    0.00020    0.00371    0.00276
N3    3    1.305780    0.522156   -0.099363    11.00000    0.08581    0.05811 =
        0.03457   -0.00187    0.00007    0.01899
AFIX  93
H3A   2    1.422638    0.476107   -0.073272    11.00000   -1.20000
H3B   2    1.275403    0.511139   -0.146252    11.00000   -1.20000
AFIX   0
N4    3    0.894449    0.782108    0.011335    11.00000    0.05776    0.04586 =
        0.04184   -0.00506   -0.00208    0.00046
C1    1    1.359490    0.697474    0.266882    11.00000    0.06921    0.04862 =
        0.03885    0.00054   -0.00460   -0.01164
C2    1    1.427473    0.693255    0.347355    11.00000    0.07188    0.06156 =
        0.04700    0.00682   -0.01196   -0.00411
AFIX  43
H2    2    1.576803    0.641685    0.365800    11.00000   -1.20000
AFIX   0
C3    1    1.279931    0.761810    0.395009    11.00000    0.07914    0.06919 =
        0.03637    0.00406   -0.01830   -0.02048
AFIX  43
H3    2    1.330356    0.756311    0.445923    11.00000   -1.20000
AFIX   0
C4    1    1.047010    0.843650    0.371347    11.00000    0.06659    0.05842 =
        0.03825   -0.00378    0.00076   -0.02433
```

```
C5    1    0.898223    0.915265    0.423055    11.00000    0.08559    0.07381 =
         0.03386   -0.00880    0.00065   -0.02634
AFIX  43
H5    2    0.948896    0.908272    0.473926    11.00000   -1.20000
AFIX   0
C6    1    0.681392    0.994754    0.400479    11.00000    0.08208    0.07121 =
         0.05332   -0.01888    0.01469   -0.01382
AFIX  43
H6    2    0.587866    1.042410    0.435310    11.00000   -1.20000
AFIX   0
C7    1    0.601943    1.003635    0.324724    11.00000    0.06935    0.06545 =
         0.05101   -0.00796    0.00462   -0.00178
AFIX  43
H7    2    0.451165    1.056642    0.308993    11.00000   -1.20000
AFIX   0
C8    1    0.743179    0.935250    0.273123    11.00000    0.06461    0.06087 =
         0.04087   -0.00159    0.00019   -0.00830
AFIX  43
H8    2    0.687906    0.943491    0.222608    11.00000   -1.20000
AFIX   0
C9    1    0.968758    0.853075    0.293964    11.00000    0.05619    0.04942 =
         0.03367   -0.00095    0.00007   -0.01702
C10   1    1.124755    0.777884    0.240784    11.00000    0.05516    0.04292 =
         0.03515    0.00189   -0.00020   -0.01039
C11   1    1.038895    0.779186    0.166198    11.00000    0.06320    0.04007 =
         0.03971    0.00086   -0.00515   -0.00412
AFIX  43
H11   2    0.881995    0.829698    0.150473    11.00000   -1.20000
AFIX   0
C12   1    1.091721    0.706242    0.040238    11.00000    0.06054    0.04476 =
         0.03257    0.00237   -0.00097   -0.00791
C13   1    1.170420    0.611475   -0.066810    11.00000    0.06281    0.04575 =
         0.03590   -0.00042    0.00692   -0.00035
```

```
C14   1    0.978035    0.691656   -0.106490    11.00000    0.05104    0.04426 =
         0.03574    0.00055   -0.00008   -0.00927
C15   1    0.839686    0.774960   -0.063588    11.00000    0.05277    0.04665 =
         0.03496   -0.00239   -0.00522   -0.00602
C16   1    0.619837    0.864460   -0.094022    11.00000    0.05594    0.06020 =
         0.04648   -0.01122   -0.01085    0.00573
AFIX  23
H16A  2    0.438920    0.867660   -0.063915    11.00000   -1.20000
H16B  2    0.571511    0.848972   -0.145513    11.00000   -1.20000
AFIX   0
C17   1    0.731879    0.972490   -0.093568    11.00000    0.08795    0.05179 =
         0.08462    0.00233   -0.03228    0.00698
AFIX 137
H17A  2    0.808426    0.983545   -0.044450    11.00000   -1.50000
H17B  2    0.571904    1.028182   -0.105070    11.00000   -1.50000
H17C  2    0.886726    0.974607   -0.130896    11.00000   -1.50000
AFIX   0
C18   1    0.940957    0.685305   -0.190453    11.00000    0.05336    0.04536 =
         0.03893    0.00012   -0.00072   -0.00422
C19   1    1.030220    0.761935   -0.239937    11.00000    0.07253    0.05666 =
         0.04353    0.00583   -0.00932   -0.01588
AFIX  43
H19   2    1.112091    0.819797   -0.220837    11.00000   -1.20000
AFIX   0
C20   1    1.001206    0.755110   -0.317715    11.00000    0.09196    0.06916 =
         0.04561    0.01483   -0.00366   -0.02119
AFIX  43
H20   2    1.061097    0.807895   -0.350439    11.00000   -1.20000
AFIX   0
C21   1    0.882531    0.669017   -0.345280    11.00000    0.08426    0.08161 =
         0.03437   -0.00273   -0.00423   -0.01290
C22   1    0.795820    0.590999   -0.298596    11.00000    0.10743    0.07921 =
         0.04463   -0.00893   -0.00591   -0.03502
```

```
AFIX  43
H22   2    0.716106    0.532999   -0.318188    11.00000   -1.20000
AFIX   0
C23   1    0.827735    0.598919   -0.221049    11.00000    0.08686    0.06283 =
        0.04518    0.00211    0.00356   -0.02785
AFIX  43
H23   2    0.771632    0.544828   -0.188875    11.00000   -1.20000
AFIX   0
HKLF 5


REM  PYMTVAN_0m in P-1 YELLOW NEEDLE
REM wR2 = 0.2358, GooF = S = 1.139, Restrained GooF = 1.139 for all data
REM R1 = 0.0763 for 2397 Fo > 4sig(Fo) and 0.1311 for all 4351 data
REM 264 parameters refined using 0 restraints

END
```

Table S5: Selected structural parameters for the compounds **DSPIN** and **ACPIN**, calculated using the B3LYP/6-311+G(d,p) approach with the implicit solvent effects from ethanol.

**Selected distances (Å) in DSPIN**		**Selected distances (Å) in ACPIN**	
H11—H13	2.070	H11—N4	2.394
H11—N1	2.055	H1—N2	2.468
H13—N1	2.744	H3—N2	2.456
N2—H3	2.534	-	-
**Selected dihedral angles (°) in DSPIN**		**Selected dihedral angles (°) in ACPIN**	
N1—C11—C10—C1	-0.566	N1—C11—C10—C1	-0.042
C11—C10—C1—O1	0.658	C11—C10—C1—O1	0.257
H1—N1—C1—O1	-1.358	H1—N1—C1—O1	0.102
H13—C13—C11—H1	-13.103	H11—C11—C12—N4	-0.139
O3—S1—C18—S2	-2.349	C15—C14—C18—C19	75.940
C10—C11—N1—C12	179.224	C10—C11—N1—C12	179.570

Table S6. Second-order perturbation theory analysis results for the compounds **DSPIN** and **ACPIN**, calculated using the B3LYP/6-311+G(d,p) approach with the implicit solvent effects from ethanol.

Compound	Donor	Type	Acceptor	Type	E(2),[a] kcal/mol	E(j)-E(i), A.U.	F(i,j), A.U.
**DSPIN**	S1-C15	σ	C13-C14	π*	11.88	0.05	0.024
	S2-C18	σ	C13-C14	π*	73.93	0.03	0.048
	S2-C19	σ	C13-C14	π*	488.92	0.01	0.078
	O1-C1	π	C10	LP*	16.04	0.09	0.052
	N1-C11	σ	C13-C14	π*	45.75	0.23	0.100
	N1-C11	σ	C16-H16	σ*	721.29	0.03	0.137
	N1-C11	σ	C16-C17	σ*	10.00	0.84	0.082
	N1-C11	σ	C10	LP*	67.16	0.11	0.113
	N1-C11	π	O1-C1	π*	16.96	0.22	0.059
	N1-C11	π	N1-C11	π*	11.58	0.16	0.043
	N1-C11	π	N3-C18	σ*	11.91	0.53	0.072
	N1-C11	π	C3-C4	σ*	14.40	0.52	0.079
	N1-C11	π	C8-H8	σ*	19.88	0.42	0.084
	N1-C11	π	C12-C17	π*	28.92	0.36	0.098
	N1-C11	π	C14-H14	σ*	11.58	0.54	0.072
	N1-C11	π	C14-C15	σ*	14.94	0.66	0.090
	N1-C11	π	C16-C17	σ*	39.19	0.34	0.106
	N1-C11	π	C19-C20	σ*	14.83	1.65	0.143
	N1-C12	σ	O1-C1	σ*	12.76	1.16	0.109
	N1-C12	σ	O1-C1	π*	11.77	0.72	0.091
	N1-C12	σ	N1-C11	π*	12.79	0.66	0.094
	N1-C12	σ	N3-H3	σ*	14.54	1.03	0.110
	N1-C12	σ	N3-C18	σ*	18.32	1.03	0.124
	N1-C12	σ	N3-C20	σ*	11.80	1.09	0.101
	N1-C12	σ	C3-C4	σ*	22.97	1.02	0.137
	N1-C12	σ	C5-H5	σ*	11.25	1.13	0.101
	N1-C12	σ	C8-H8	σ*	29.43	0.92	0.147

	N1-C12	σ	C13-C14	σ*	232.83	0.23	0.225
	N1-C12	σ	C14-H14	σ*	18.62	1.04	0.124
	N1-C12	σ	C14-C15	σ*	27.12	1.15	0.158
	N1-C12	σ	C16-H16	σ*	3398.88	0.03	0.297
	N1-C12	σ	C16-C17	σ*	72.22	0.84	0.220
	N1-C12	σ	C19-C20	σ*	33.24	2.15	0.239
	N3-H3	σ	O1-C1	π*	11.45	0.54	0.078
	N3-H3	σ	N3-C18	σ*	15.56	0.85	0.103
	N3-H3	σ	C7-H7	σ*	20.49	1.03	0.130
	N3-H3	σ	C13-C14	π*	411.36	0.06	0.145
	N3-C18	σ	C14-C15	σ*	13.79	1.15	0.113
	N3-C18	σ	C16-H16	σ*	1845.82	0.03	0.208
	N3-C18	σ	C16-C17	σ*	11.91	0.84	0.089
	N3-C18	σ	C19-C20	σ*	14.53	2.15	0.158
	N3-C20	σ	C10	LP*	58.17	0.73	0.279
	N3-C20	σ	S1-N2	σ*	12.45	1.09	0.109
	N3-C20	σ	O1-C1	σ*	64.05	1.28	0.256
	N3-C20	σ	O1-C1	π*	87.73	0.84	0.269
	N3-C20	σ	N1-C10	π*	51.63	0.78	0.205
	N3-C20	σ	N1-C12	σ*	17.15	1.35	0.136
	N3-C20	σ	N2-C18	σ*	50.01	1.40	0.236
	N3-C20	σ	N3-H3	σ*	83.44	1.15	0.277
	N3-C20	σ	N3-C18	σ*	151.39	1.15	0.375
	N3-C20	σ	N3-C20	σ*	36.47	1.21	0.187
	N3-C20	σ	C1-C10	σ*	12.80	1.40	0.121
	N3-C20	σ	C2-H2	σ*	18.56	1.36	0.142
	N3-C20	σ	C3-C4	σ*	117.54	1.14	0.327
	N3-C20	σ	C4-C9	σ*	19.73	1.43	0.151
	N3-C20	σ	C5-H5	σ*	45.14	1.25	0.212
	N3-C20	σ	C6-H6	σ*	11.61	1.38	0.113
	N3-C20	σ	C6-C7	σ*	30.48	1.46	0.188
	N3-C20	σ	C7-H7	σ*	108.06	1.32	0.338

	N3-C20	σ	C8-H8	σ*	131.77	1.04	0.330
	N3-C20	σ	C9-C10	σ*	22.32	1.42	0.160
	N3-C20	σ	C11-H11	σ*	24.91	1.28	0.159
	N3-C20	σ	C13-C14	π*	969.38	0.35	0.563
	N3-C20	σ	C14-H14	σ*	83.01	1.16	0.277
	N3-C20	σ	C14-C15	σ*	78.10	1.27	0.282
	N3-C20	σ	C15-C16	π*	12.92	0.96	0.110
	N3-C20	σ	C16-H16	σ*	2881.41	0.15	0.590
	N3-C20	σ	C16-C17	σ*	196.69	0.96	0.388
	N3-C20	σ	C19-C20	σ*	207.91	2.27	0.613
	N3-C20	σ	C19-C20	π*	13.27	2.57	0.174
	C1-C2	σ	O1-C1	σ*	24.75	1.01	0.141
	C1-C2	σ	O1-C1	π*	13.65	0.56	0.087
	C1-C2	σ	N1-C10	π*	16.22	0.50	0.092
	C1-C2	σ	N3-H3	σ*	16.27	0.88	0.107
	C1-C2	σ	N3-C18	σ*	19.40	0.87	0.117
	C1-C2	σ	N3-C20	σ*	12.29	0.93	0.096
	C1-C2	σ	C1-C10	σ*	10.15	1.13	0.096
	C1-C2	σ	C3-C4	σ*	24.50	0.86	0.130
	C1-C2	σ	C5-H5	σ*	12.33	0.97	0.098
	C1-C2	σ	C8-H8	σ*	31.78	0.76	0.139
	C1-C2	σ	C13-C14	π*	652.58	0.08	0.214
	C1-C2	σ	C14-H14	σ*	20.55	0.88	0.120
	C1-C2	σ	C14-C15	σ*	27.87	1.00	0.149
	C1-C2	σ	C16-C17	σ*	55.81	0.68	0.175
	C1-C2	σ	C19-C20	σ*	32.91	1.99	0.229
	C2-C3	π	C10	LP*	27.40	0.16	0.078
	C2-C3	π	O1-C1	π*	86.00	0.11	0.093
	C3-C4	σ	C13-C14	π*	96.51	0.03	0.050
	C5-C6	π	C10	LP*	41.86	0.15	0.088
	C5-C6	π	C7-C8	π*	20.55	0.28	0.068
	C7-C8	π	C9	LP*	19.68	0.35	0.091

	C7-C8	π	C10	LP*	130.61	0.19	0.185
	C7-C8	π	S1-N2	σ*	38.33	0.55	0.133
	C7-C8	π	O1-C1	σ*	70.65	0.74	0.221
	C7-C8	π	O1-C1	π*	131.51	0.30	0.182
	C7-C8	π	N1-C11	π*	168.05	0.24	0.188
	C7-C8	π	N1-C12	σ*	14.71	0.81	0.105
	C7-C8	π	N2-C18	σ*	35.24	0.86	0.167
	C7-C8	π	N3-H3	σ*	76.43	0.61	0.208
	C7-C8	π	N3-C18	σ*	113.45	0.61	0.252
	C7-C8	π	N3-C20	σ*	64.25	0.67	0.200
	C7-C8	π	C1-C10	σ*	18.61	0.87	0.121
	C7-C8	π	C2-H2	σ*	25.20	0.82	0.139
	C7-C8	π	C3-C4	σ*	130.11	0.60	0.268
	C7-C8	π	C4-C9	σ*	23.14	0.89	0.138
	C7-C8	π	C5-H5	σ*	59.27	0.71	0.198
	C7-C8	π	C5-C6	σ*	12.10	0.49	0.069
	C7-C8	π	C6-H6	σ*	14.18	0.84	0.105
	C7-C8	π	C6-C7	σ*	25.16	0.92	0.146
	C7-C8	π	C7-H7	σ*	39.74	0.78	0.170
	C7-C8	π	C8-H8	σ*	170.45	0.50	0.281
	C7-C8	π	C9-C10	σ*	17.31	0.88	0.119
	C7-C8	π	C11-H11	σ*	36.39	0.74	0.158
	C7-C8	π	C12-C13	σ*	10.35	0.96	0.096
	C7-C8	π	C12-C17	π*	16.40	0.44	0.077
	C7-C8	π	C14-H14	σ*	109.14	0.62	0.250
	C7-C8	π	C14-C15	σ*	145.20	0.73	0.314
	C7-C8	π	C15-C16	π*	35.76	0.42	0.112
	C7-C8	π	C16-C17	σ*	311.99	0.42	0.349
	C7-C8	π	C19-C20	σ*	168.27	1.73	0.520
	C7-C8	π	C19-C20	π*	41.34	2.03	0.264
	C8-H8	σ	C4-C9	σ*	11.67	0.97	0.095
	C8-H8	σ	C6-C7	σ*	12.28	0.99	0.099

	C8-H8	σ	C8-H8	σ*	16.39	0.58	0.087
	C8-H8	σ	C16-C17	σ*	24.59	0.50	0.099
	C8-C9	σ	C13-C14	π*	231.30	0.06	0.113
	C8-C9	σ	C16-C17	σ*	16.68	0.67	0.094
	C8-C9	σ	C19-C20	σ*	12.61	1.98	0.141
	C12-C13	σ	C13-C14	π*	19.77	0.08	0.038
	C12-C17	σ	O1-C1	σ*	11.17	1.04	0.097
	C12-C17	σ	O1-C1	π*	10.20	0.60	0.077
	C12-C17	σ	N1-C11	σ*	12.24	0.54	0.083
	C12-C17	σ	N3-H3	σ*	11.74	0.91	0.092
	C12-C17	σ	N3-C18	σ*	15.15	0.91	0.105
	C12-C17	σ	C3-C4	σ*	18.64	0.90	0.115
	C12-C17	σ	C8-H8	σ*	23.39	0.80	0.122
	C12-C17	σ	C13-C14	π*	333.15	0.11	0.184
	C12-C17	σ	C14-H14	σ*	15.18	0.92	0.106
	C12-C17	σ	C14-C15	σ*	20.97	1.03	0.131
	C12-C17	σ	C16-C17	σ*	72.89	0.72	0.205
	C12-C17	σ	C19-C20	σ*	32.78	2.03	0.231
	C12-C17	π	C10	LP*	82.01	0.01	0.038
	C12-C17	π	O1-C1	π*	16.42	0.12	0.040
	C12-C17	π	C8-H8	σ*	12.14	0.32	0.062
	C12-C17	π	C15-C16	π*	12.95	0.24	0.050
	C12-C17	π	C16-C17	σ*	24.11	0.24	0.076
	C13-C14	π	C12-C17	π*	24.51	0.25	0.071
	C13-C14	π	C15-C16	π*	21.09	0.23	0.063
	C14-C15	σ	C8-H8	σ*	11.40	0.80	0.085
	C14-C15	σ	C13-C14	π*	158.26	0.11	0.126
	C14-C15	σ	C14-H14	σ*	16.31	0.91	0.109
	C14-C15	σ	C14-C15	σ*	10.33	1.03	0.092
	C14-C15	σ	C16-C17	σ*	20.20	0.72	0.108
	C14-C15	σ	C19-C20	σ*	16.24	2.03	0.162
	C15-C16	π	C12-C17	π*	16.49	0.26	0.059

	C19-H19	σ	C10	LP*	17.35	0.33	0.102
	C19-H19	σ	O1-C1	π*	21.29	0.44	0.096
	C19-H19	σ	N3-C18	σ*	35.50	0.75	0.147
	C19-H19	σ	C3-C4	σ*	18.19	0.74	0.104
	C19-H19	σ	C7-H7	σ*	24.00	0.93	0.133
	C19-H19	σ	C8-H8	σ*	20.22	0.64	0.102
	C19-H19	σ	C14-H14	σ*	10.37	0.76	0.079
	C19-H19	σ	C16-C17	σ*	29.30	0.56	0.115
	C19-H19	σ	C10	LP*	32.14	0.41	0.156
	C19-C20	σ	O1-C1	σ*	14.12	0.97	0.105
	C19-C20	σ	O1-C1	π*	0.97	0.52	0.146
	C19-C20	σ	N1-C11	π*	12.50	0.46	0.078
	C19-C20	σ	N3-H3	σ*	37.62	0.84	0.159
	C19-C20	σ	N3-C18	σ*	83.30	0.84	0.237
	C19-C20	σ	C3-C4	σ*	32.01	0.82	0.145
	C19-C20	σ	C7-H7	σ*	53.34	1.01	0.207
	C19-C20	σ	C8-H8	σ*	32.23	0.73	0.137
	C19-C20	σ	C13-C14	π*	1909.55	0.04	0.262
	C19-C20	σ	C14-H14	σ*	17.35	0.84	0.108
	C19-C20	σ	C14-C15	σ*	12.04	0.96	0.096
	C19-C20	σ	C16-C17	σ*	42.21	0.65	0.147
	C19-C20	σ	C19-C20	σ*	11.45	1.96	0.134
	C19-C20	σ	C19-C20	π*	12.62	2.25	0.159
	C19-C20	σ	C10	LP*	10.99	0.04	0.029
	C19-C20	π	N1-C11	π*	10.25	0.09	0.03
	C19-C20	π	C16-C17	σ*	15.59	0.28	0.059
	C19-C20	π	C19-C20	σ*	18.87	1.59	0.156
	C20-H20	σ	S1-N2	σ*	108.32	0.30	0.169
	C20-H20	σ	S1-C15	σ*	11.29	0.36	0.060
	C20-H20	σ	S2-C18	σ*	15.63	0.34	0.066
	C20-H20	σ	S2-C19	σ*	12.63	0.40	0.063
	C20-H20	σ	O1-C1	σ*	153.06	0.50	0.247

	C20-H20	σ	O1-C1	π*	514.97	0.05	0.160
	C20-H20	σ	N1-C12	σ*	25.31	0.56	0.107
	C20-H20	σ	N2-C18	σ*	58.84	0.61	0.169
	C20-H20	σ	N3-H3	σ*	127.53	0.36	0.193
	C20-H20	σ	N3-C18	σ*	123.77	0.36	0.190
	C20-H20	σ	N3-C20	σ*	178.70	0.42	0.245
	C20-H20	σ	C1-C10	σ*	35.48	0.62	0.133
	C20-H20	σ	C2-H2	σ*	47.34	0.57	0.147
	C20-H20	σ	C3-C4	σ*	280.99	0.35	0.281
	C20-H20	σ	C4-C5	σ*	17.90	0.71	0.101
	C20-H20	σ	C4-C9	σ*	43.58	0.64	0.150
	C20-H20	σ	C5-H5	σ*	128.94	0.46	0.219
	C20-H20	σ	C6-H6	σ*	32.84	0.60	0.125
	C20-H20	σ	C6-C7	σ*	44.44	0.67	0.15
	C20-H20	σ	C7-H7	σ*	26.15	0.54	0.106
	C20-H20	σ	C8-H8	σ*	483.59	0.25	0.313
	C20-H20	σ	C9-C10	σ*	35.16	0.64	0.134
	C20-H20	σ	C11-H11	σ*	94.60	0.49	0.193
	C20-H20	σ	C12-C13	σ*	43.74	0.71	0.157
	C20-H20	σ	C12-C17	σ*	13.27	0.73	0.088
	C20-H20	σ	C12-C17	π*	40.87	0.19	0.086
	C20-H20	σ	C14-H14	σ*	232.13	0.37	0.263
	C20-H20	σ	C14-C15	σ*	297.77	0.49	0.340
	C20-H20	σ	C15-C16	σ*	16.07	0.81	0.102
	C20-H20	σ	C15-C16	π*	85.43	0.17	0.119
	C20-H20	σ	C16-C17	σ*	1183.19	0.17	0.404
	C20-H20	σ	C19-C20	σ*	254.36	1.48	0.550
	C20-H20	σ	C19-C20	π*	41.82	1.78	0.256
	S2	LP	N2-C18	π*	35.28	0.21	0.082
	O1	LP	N1-H1	σ*	16.20	0.67	0.094
	O1	LP	C1-C2	σ*	15.17	0.79	0.099
	O1	LP	C1-C10	σ*	12.60	0.70	0.085

	O2	LP	C10	LP*	27.79	0.05	0.046
	O2	LP	S1-N2	σ*	33.71	0.41	0.106
	O2	LP	S1-C15	σ*	16.06	0.47	0.078
	O2	LP	O1-C1	π*	15.95	0.16	0.048
	O2	LP	N1-C11	π*	26.86	0.10	0.050
	O2	LP	C3-C4	σ*	12.82	0.46	0.071
	O2	LP	C8-H8	σ*	18.50	0.36	0.076
	O2	LP	C14-C15	σ*	14.20	0.59	0.085
	O2	LP	C16-C17	σ*	38.46	0.28	0.096
	O2	LP	C19-C20	σ*	11.85	1.59	0.128
	O2	LP	S1-O3	σ*	21.00	0.56	0.097
	O2	LP	S1-N2	σ*	10.24	0.39	0.057
	O3	LP	S1-C15	σ*	15.65	0.46	0.076
	O3	LP	S1-O2	σ*	16.80	0.57	0.088
	O3	LP	S1-N2	σ*	19.54	0.40	0.079
	N2	LP	S2-C18	σ*	19.06	0.54	0.092
	N2	LP	N2-C18	π*	68.12	0.24	0.117
	C16	LP	C2-C3	π*	48.99	0.15	0.099
	C16	LP	C5-C6	π*	63.96	0.14	0.104
	C10	LP	C9	LP*	110.94	0.16	0.123
	C10	LP	S1-N2	σ*	33.64	0.36	0.119
	C10	LP	O1-C1	σ*	48.54	0.55	0.193
	C10	LP	O1-C1	π*	14.12	0.11	0.040
	C10	LP	N1-C11	π*	23.53	0.05	0.034
	C10	LP	N2-C18	σ*	24.53	0.67	0.150
	C10	LP	N3-H3	σ*	61.89	0.42	0.189
	C10	LP	N3-C18	σ*	73.59	0.42	0.204
	C10	LP	N3-C20	σ*	42.25	0.48	0.167
	C10	LP	C1-C10	σ*	12.17	0.68	0.105
	C10	LP	C2-H2	σ*	16.26	0.63	0.119
	C10	LP	C3-C4	σ*	94.51	0.41	0.230
	C10	LP	C4-C9	σ*	14.95	0.70	0.119

	C10	LP	C5-H5	σ*	40.85	0.52	0.171
	C10	LP	C6-C7	σ*	15.75	0.73	0.126
	C10	LP	C7-H7	σ*	22.29	0.59	0.135
	C10	LP	C8-H8	σ*	138.98	0.31	0.244
	C10	LP	C9-C10	σ*	11.39	0.69	0.104
	C10	LP	C11-H11	σ*	25.91	0.55	0.140
	C10	LP	C12-C17	π*	16.34	0.25	0.065
	C10	LP	C14-H14	σ*	77.78	0.43	0.214
	C10	LP	C14-C15	σ*	98.43	0.54	0.270
	C10	LP	C15-C16	π*	39.87	0.23	0.097
	C10	LP	C16-C17	σ*	292.73	0.23	0.305
	C10	LP	C19-C20	σ*	89.46	1.54	0.435
	C10	LP	C19-C20	π*	23.71	1.84	0.226
**ACPIN**	Cl1-C21	σ	C22-C23	σ*	10.92	1.07	0.097
	N1-C11	σ	C1	LP*	15.24	0.75	0.123
	N1-C11	σ	Cl1-C21	σ*	22.06	0.98	0.132
	N1-C11	σ	O1-C1	σ*	13.43	1.41	0.123
	N1-C11	σ	C1-C2	σ*	54.37	1.09	0.219
	N1-C11	σ	C1-C10	σ*	23.67	1.28	0.157
	N1-C11	σ	C4-C5	σ*	94.15	0.96	0.269
	N1-C11	σ	C4-C5	π*	68.66	0.66	0.213
	N1-C11	σ	C4-C9	σ*	44.00	1.18	0.204
	N1-C11	σ	C6-C7	σ*	18.50	1.45	0.146
	N1-C11	σ	C6-C7	π*	17.24	0.92	0.125
	N1-C11	σ	C7-C8	σ*	135.10	0.84	0.301
	N1-C11	σ	C8-H8	σ*	175.12	0.68	0.308
	N1-C11	σ	C8-C9	σ*	25.94	1.31	0.165
	N1-C11	σ	C9-C10	σ*	30.07	1.33	0.179
	N1-C11	σ	C10-C11	π*	13.04	0.91	0.105
	N1-C11	σ	C13-C18	σ*	10.20	1.41	0.108
	N1-C11	σ	C15-C16	σ*	37.42	1.22	0.191
	N1-C11	σ	C16-H16A	σ*	213.17	0.59	0.317

	N1-C11	σ	C17-H17A	σ*	13.95	1.33	0.122
	N1-C11	σ	C18-C19	π*	125.52	0.64	0.278
	N1-C11	σ	C18-C23	σ*	11.38	3.35	0.175
	N1-C11	σ	C19-C20	σ*	111.63	1.04	0.304
	N1-C11	σ	C20-H20	σ*	19.91	1.28	0.143
	N1-C11	σ	C20-C21	σ*	163.51	0.87	0.339
	N1-C11	σ	C20-C21	π*	4137.13	0.04	0.388
	N1-C11	σ	C22-C23	σ*	318.92	1.34	0.584
	N1-C11	σ	C22-C23	π*	210.62	1.15	0.476
	N1-C12	σ	C1	LP*	36.66	0.97	0.217
	N1-C12	σ	Cl1-C21	σ*	55.54	1.20	0.231
	N1-C12	σ	O1-C1	σ*	31.19	1.63	0.202
	N1-C12	σ	N1-C11	σ*	11.22	1.69	0.123
	N1-C12	σ	N3-H3A	σ*	16.50	1.57	0.144
	N1-C12	σ	N3-C13	σ*	23.81	1.59	0.174
	N1-C12	σ	N4-C12	σ*	28.51	1.67	0.196
	N1-C12	σ	N4-C15	σ*	11.63	1.70	0.126
	N1-C12	σ	C1-C2	σ*	142.93	1.31	0.389
	N1-C12	σ	C1-C10	σ*	58.66	1.50	0.267
	N1-C12	σ	C2-C3	σ*	21.08	1.75	0.172
	N1-C12	σ	C2-C3	π*	22.85	1.18	0.15
	N1-C12	σ	C3-H3	σ*	25.36	1.56	0.17
	N1-C12	σ	C4-C5	σ*	233.44	1.17	0.468
	N1-C12	σ	C4-C5	π*	159.87	0.88	0.374
	N1-C12	σ	C4-C9	σ*	111.39	1.39	0.353
	N1-C12	σ	C6-C7	σ*	50.19	1.66	0.258
	N1-C12	σ	C6-C7	π*	43.05	1.14	0.219
	N1-C12	σ	C7-C8	σ*	342.15	1.05	0.536
	N1-C12	σ	C8-H8	σ*	404.57	0.89	0.537
	N1-C12	σ	C8-C9	σ*	76.17	1.52	0.305
	N1-C12	σ	C9-C10	σ*	44.21	1.55	0.234
	N1-C12	σ	C10-C11	π*	32.57	1.13	0.185

	N1-C12	σ	C13-C14	σ*	25.55	1.62	0.183
	N1-C12	σ	C15-C16	σ*	102.89	1.43	0.344
	N1-C12	σ	C16-H16A	σ*	486.04	0.81	0.560
	N1-C12	σ	C16-H16B	σ*	3645.75	0.19	0.739
	N1-C12	σ	C17-H17A	σ*	36.77	1.55	0.213
	N1-C12	σ	C18-C19	π*	292.45	0.86	0.490
	N1-C12	σ	C18-C23	σ*	32.68	3.57	0.30
	N1-C12	σ	C19-C20	σ*	284.68	1.26	0.534
	N1-C12	σ	C20-H20	σ*	52.51	1.50	0.251
	N1-C12	σ	C20-C21	σ*	404.70	1.09	0.595
	N1-C12	σ	C20-C21	π*	1853.10	0.25	0.678
	N1-C12	σ	C22-C23	σ*	854.05	1.56	1.029
	N1-C12	σ	C22-C23	π*	549.35	1.36	0.838
	N2-C12	σ	C8-H8	σ*	11.48	0.55	0.071
	N2-C12	σ	C16-H16B	σ*	15.19	0.47	0.075
	N2-C12	σ	C22-C23	σ*	19.62	1.21	0.138
	N2-C12	σ	C22-C23	π*	11.89	1.02	0.107
	N2-C13	π	N4-C12	π*	40.61	0.31	0.103
	N2-C13	σ	C1-C2	σ*	22.74	1.02	0.137
	N2-C13	σ	C1-C10	σ*	14.43	1.21	0.119
	N2-C13	σ	C4-C5	σ*	54.14	0.89	0.196
	N2-C13	σ	C4-C5	π*	36.89	0.59	0.148
	N2-C13	σ	C4-C9	σ*	23.23	1.11	0.144
	N2-C13	σ	C6-C7	σ*	10.39	1.38	0.107
	N2-C13	σ	C6-C7	π*	10.59	0.85	0.094
	N2-C13	σ	C7-C8	σ*	71.83	0.77	0.21
	N2-C13	σ	C8-H8	σ*	87.74	0.61	0.206
	N2-C13	σ	C8-C9	σ*	16.17	1.24	0.127
	N2-C13	σ	C13-C14	σ*	13.28	1.34	0.120
	N2-C13	σ	C15-C16	σ*	14.75	1.15	0.117
	N2-C13	σ	C16-H16B	σ*	128.65	0.52	0.232
	N2-C13	σ	C18-C19	π*	44.47	0.57	0.156

	N2-C13	σ	C19-C20	σ*	76.74	0.97	0.244
	N2-C13	σ	C20-C21	σ*	111.52	0.80	0.269
	N2-C13	σ	C22-C23	σ*	198.68	1.27	0.449
	N2-C13	σ	C22-C23	π*	130.96	1.08	0.364
	N4-C12	π	C8-H8	σ*	22.95	0.01	0.016
	C2-C3	π	C1	LP*	169.24	0.04	0.085
	C4-C5	π	C1	LP*	13.36	0.02	0.014
	C4-C5	π	C2-C3	π*	32.58	0.23	0.082
	C4-C5	π	C6-C7	π*	49.83	0.18	0.086
	C4-C5	π	C7-C8	σ*	18.42	0.10	0.043
	C4-C5	π	C8-C9	π*	22.75	0.28	0.071
	C4-C5	π	C20-C21	σ*	21.16	0.14	0.053
	C4-C5	π	C22-C23	σ*	12.57	0.60	0.086
	C4-C5	π	C22-C23	π*	14.04	0.41	0.069
	C4-C9	σ	C4-C5	σ*	27.48	0.65	0.120
	C4-C9	σ	C8-H8	σ*	42.41	0.37	0.113
	C4-C9	σ	C16-H16B	σ*	19.54	0.29	0.067
	C4-C9	σ	C20-C21	σ*	11.46	0.57	0.072
	C4-C9	σ	C22-C23	σ*	12.72	1.04	0.103
	C4-C9	σ	C22-C23	π*	14.59	0.84	0.107
	C6-H6	σ	C4-C5	σ*	44.10	0.50	0.132
	C6-H6	σ	C4-C5	π*	19.45	0.20	0.063
	C6-H6	σ	C8-H8	σ*	52.16	0.22	0.095
	C6-H6	σ	C16-H16B	σ*	80.08	0.13	0.091
	C6-H6	σ	C18-C19	π*	34.71	0.18	0.077
	C6-H6	σ	C19-C20	σ*	17.42	0.58	0.090
	C6-H6	σ	C20-C21	σ*	30.13	0.41	0.100
	C6-H6	σ	C22-C23	σ*	37.78	0.88	0.163
	C6-H6	σ	C22-C23	π*	32.30	0.69	0.144
	C6-C7	σ	C8-H8	σ*	10.88	0.38	0.058
	C6-C7	σ	C8-C9	π*	18.83	0.28	0.065
	C7-H7	σ	C8-H8	σ*	14.63	0.21	0.049

	C8-C9	π	C1	LP*	22.37	0.02	0.019
	C8-C9	π	C6-C7	π*	25.79	0.19	0.062
	C8-C9	π	C10-C11	π*	10.53	0.18	0.040
	C10-C11	π	C1	LP*	233.38	0.03	0.093
	C10-C11	π	C8-C9	π*	18.92	0.30	0.068
	C13-C14	σ	C14-C15	σ*	13.60	0.93	0.101
	C13-C14	σ	C18-C19	σ*	33.62	0.36	0.106
	C14-C15	π	N2-C13	π*	33.02	0.25	0.085
	C14-C15	π	N4-C12	π*	10.48	0.26	0.048
	C14-C15	π	C7-C8	σ*	11.60	0.12	0.036
	C14-C18	σ	Cl1-C21	σ*	10.20	0.63	0.072
	C14-C18	σ	C1-C2	σ*	29.89	0.75	0.134
	C14-C18	σ	C4-C5	π*	20.60	0.32	0.080
	C14-C18	σ	C7-C8	σ*	34.08	0.49	0.116
	C14-C18	σ	C8-H8	σ*	68.48	0.33	0.135
	C14-C18	σ	C15-C16	σ*	19.75	0.87	0.117
	C14-C18	σ	C16-H16B	σ*	44.62	0.25	0.09
	C14-C18	σ	C18-C19	π*	140.64	0.29	0.198
	C17-H17A	σ	C16-H16B	σ*	15.28	0.10	0.035
	C18-C19	σ	C18-C19	π*	19.84	0.36	0.082
	C18-C19	π	C22-C23	π*	21.82	0.42	0.086
	C18-C23	σ	C1	LP*	47.34	0.50	0.177
	C18-C23	σ	Cl1-C21	σ*	71.39	0.73	0.204
	C18-C23	σ	O1-C1	σ*	32.25	1.17	0.174
	C18-C23	σ	N3-H3B	σ*	13.36	1.10	0.109
	C18-C23	σ	N3-C13	σ*	23.52	1.13	0.146
	C18-C23	σ	N4-C12	σ*	16.67	1.21	0.127
	C18-C23	σ	C1-C2	σ*	186.71	0.85	0.356
	C18-C23	σ	C1-C10	σ*	36.44	1.03	0.174
	C18-C23	σ	C2-C3	σ*	13.89	1.29	0.120
	C18-C23	σ	C2-C3	π*	23.00	0.72	0.119
	C18-C23	σ	C3-H3	σ*	20.72	1.10	0.135

	C18-C23	σ	C4-C5	σ*	192.97	0.71	0.331
	C18-C23	σ	C4-C5	π*	207.61	0.42	0.292
	C18-C23	σ	C4-C9	σ*	100.53	0.93	0.273
	C18-C23	σ	C6-C7	σ*	32.05	1.20	0.176
	C18-C23	σ	C6-C7	π*	27.87	0.67	0.135
	C18-C23	σ	C7-C8	σ*	385.71	0.59	0.428
	C18-C23	σ	C8-H8	σ*	581.82	0.43	0.448
	C18-C23	σ	C8-C9	σ*	52.23	1.06	0.210
	C18-C23	σ	C9-C10	σ*	35.78	1.08	0.176
	C18-C23	σ	C10-C11	π*	21.98	0.67	0.116
	C18-C23	σ	C13-C14	σ*	25.86	1.16	0.155
	C18-C23	σ	C15-C16	σ*	166.52	0.97	0.359
	C18-C23	σ	C16-H16B	σ*	604.48	0.34	0.409
	C18-C23	σ	C17-H17A	σ*	63.59	1.08	0.236
	C18-C23	σ	C18-C19	π*	758.97	0.39	0.532
	C18-C23	σ	C18-C23	σ*	17.47	3.11	0.208
	C18-C23	σ	C19-C20	σ*	126.43	0.79	0.284
	C18-C23	σ	C20-H20	σ*	41.35	1.04	0.186
	C18-C23	σ	C20-C21	σ*	214.08	0.63	0.327
	C18-C23	σ	C22-C23	σ*	389.83	1.09	0.585
	C18-C23	σ	C22-C23	π*	384.68	0.90	0.567
	C19-C20	σ	C20-C21	σ*	11.52	0.59	0.074
	C20-C21	π	C22-C23	π*	14.87	0.44	0.072
	C21-C22	σ	C18-C19	π*	26.13	0.39	0.099
	C21-C22	σ	C20-C21	σ*	19.11	0.62	0.098
	C22-H22	σ	C1-C2	σ*	36.67	0.65	0.139
	C22-H22	σ	C4-C5	σ*	18.85	0.52	0.088
	C22-H22	σ	C4-C5	π*	39.21	0.22	0.093
	C22-H22	σ	C4-C9	σ*	11.18	0.74	0.081
	C22-H22	σ	C7-C8	σ*	57.89	0.40	0.136
	C22-H22	σ	C8-H8	σ*	120.41	0.24	0.151
	C22-H22	σ	C15-C16	σ*	24.66	0.78	0.124

	C22-H22	σ	C16-H16B	σ*	107.54	0.15	0.115
	C22-H22	σ	C17-H17A	σ*	11.80	0.89	0.092
	C22-H22	σ	C18-C19	π*	225.24	0.20	0.208
	C22-H22	σ	C22-C23	σ*	60.17	0.90	0.208
	C22-H22	σ	C22-C23	π*	22.57	0.71	0.122
	C22-C23	σ	C1	LP*	48.73	0.48	0.174
	C22-C23	σ	Cl1-C21	σ*	121.29	0.70	0.261
	C22-C23	σ	O1-C1	σ*	32.26	1.14	0.172
	C22-C23	σ	N3-H3B	σ*	12.91	1.08	0.106
	C22-C23	σ	N3-C13	σ*	25.70	1.10	0.151
	C22-C23	σ	N4-C12	σ*	16.29	1.18	0.124
	C22-C23	σ	C1-C2	σ*	198.02	0.82	0.360
	C22-C23	σ	C1-C10	σ*	45.20	1.01	0.192
	C22-C23	σ	C2-C3	σ*	13.83	1.26	0.118
	C22-C23	σ	C2-C3	π*	22.92	0.69	0.116
	C22-C23	σ	C3-H3	σ*	20.17	1.07	0.132
	C22-C23	σ	C4-C5	σ*	197.10	0.68	0.328
	C22-C23	σ	C4-C5	π*	226.23	0.39	0.294
	C22-C23	σ	C4-C9	σ*	103.13	0.90	0.272
	C22-C23	σ	C6-C7	σ*	38.19	1.17	0.190
	C22-C23	σ	C6-C7	π*	34.95	0.65	0.148
	C22-C23	σ	C7-C8	σ*	402.60	0.56	0.427
	C22-C23	σ	C8-H8	σ*	624.78	0.40	0.449
	C22-C23	σ	C8-C9	σ*	60.54	1.03	0.224
	C22-C23	σ	C9-C10	σ*	35.87	1.05	0.174
	C22-C23	σ	C10-C11	π*	23.06	0.64	0.116
	C22-C23	σ	C13-C14	σ*	22.97	1.13	0.144
	C22-C23	σ	C15-C16	σ*	139.41	0.94	0.324
	C22-C23	σ	C16-H16B	σ*	642.46	0.32	0.404
	C22-C23	σ	C17-H17A	σ*	63.66	1.06	0.233
	C22-C23	σ	C18-C19	π*	827.31	0.36	0.535
	C22-C23	σ	C19-C20	σ*	138.68	0.76	0.292

	C22-C23	σ	C20-H20	σ*	54.36	1.01	0.210
	C22-C23	σ	C20-C21	σ*	266.14	0.60	0.356
	C22-C23	σ	C22-C23	σ*	832.74	1.07	0.844
	C22-C23	σ	C22-C23	π*	393.95	0.87	0.564
	C23-H23	σ	Cl1-C21	σ*	77.20	0.08	0.072
	C23-H23	σ	O2-C1	σ*	21.79	0.52	0.095
	C23-H23	σ	N3-H3B	σ*	20.65	0.46	0.087
	C23-H23	σ	N3-C13	σ*	18.35	0.48	0.084
	C23-H23	σ	N4-C12	σ*	16.21	0.56	0.085
	C23-H23	σ	C1-C2	σ*	135.13	0.20	0.147
	C23-H23	σ	C1-C10	σ*	75.75	0.39	0.154
	C23-H23	σ	C2-C3	σ*	17.67	0.64	0.095
	C23-H23	σ	C2-C3	π*	83.12	0.07	0.072
	C23-H23	σ	C3-H3	σ*	22.83	0.45	0.091
	C23-H23	σ	C4-C5	σ*	1349.18	0.06	0.260
	C23-H23	σ	C4-C9	σ*	147.28	0.28	0.183
	C23-H23	σ	C6-C7	σ*	39.33	0.55	0.132
	C23-H23	σ	C6-C7	π*	492.58	0.03	0.113
	C23-H23	σ	C8-C9	σ*	79.59	0.41	0.162
	C23-H23	σ	C9-C10	σ*	37.99	0.43	0.115
	C23-H23	σ	C10-C11	π*	654.23	0.02	0.107
	C23-H23	σ	C13-C14	σ*	19.16	0.51	0.089
	C23-H23	σ	C15-C16	σ*	92.33	0.32	0.154
	C23-H23	σ	C17-H17A	σ*	22.22	0.44	0.088
	C23-H23	σ	C18-C23	σ*	12.83	2.46	0.159
	C23-H23	σ	C19-C20	σ*	806.57	0.15	0.306
	C23-H23	σ	C20-H20	σ*	38.13	0.39	0.109
	C23-H23	σ	C22-C23	σ*	701.89	0.45	0.500
	C23-H23	σ	C22-C23	π*	709.38	0.25	0.409
	Cl1	LP	C20-C21	σ*	11.15	0.83	0.086
	Cl1	LP	C8-H8	σ*	11.24	0.02	0.013
	Cl1	LP	C20-C21	σ*	27.33	0.21	0.068

	Cl1	LP	C7-C8	σ*	10.18	0.18	0.039
	Cl1	LP	C8-H8	σ*	94.00	0.02	0.039
	Cl1	LP	C20-C21	σ*	11.49	0.22	0.045
	Cl1	LP	C22-C23	σ*	12.04	0.68	0.082
	Cl1	LP	C22-C23	π*	10.13	0.49	0.067
	O1	LP	N1-H1	σ*	11.43	0.67	0.079
	O1	LP	C1-C2	σ*	32.45	0.39	0.102
	O1	LP	C1-C10	σ*	15.72	0.58	0.086
	O1	LP	C1	LP*	1242.28	0.02	0.155
	N1	LP	N4-C12	π*	50.69	0.26	0.103
	N1	LP	C10-C11	π*	66.53	0.20	0.107
	N2	LP	N4-C12	σ*	13.02	0.83	0.094
	N2	LP	C13-C14	σ*	10.38	0.79	0.082
	N3	LP	N2-C13	σ*	53.91	0.26	0.112
	N4	LP	C1	LP*	46.37	0.23	0.115
	N4	LP	Cl1-C21	σ*	40.17	0.46	0.123
	N4	LP	O1-C1	σ*	15.88	0.89	0.109
	N4	LP	N1-C12	σ*	5.40	0.84	0.103
	N4	LP	N3-C13	σ*	14.40	0.85	0.101
	N4	LP	N4-C12	σ*	11.13	0.93	0.092
	N4	LP	C1-C2	σ*	90.14	0.57	0.205
	N4	LP	C1-C10	σ*	36.56	0.76	0.150
	N4	LP	C2-C3	π*	17.37	0.44	0.080
	N4	LP	C3-H3	σ*	12.81	0.82	0.093
	N4	LP	C4-C5	σ*	172.63	0.43	0.249
	N4	LP	C4-C5	π*	292.13	0.14	0.197
	N4	LP	C4-C9	σ*	66.76	0.65	0.190
	N4	LP	C6-C7	σ*	25.18	0.92	0.139
	N4	LP	C6-C7	π*	36.66	0.40	0.116
	N4	LP	C7-C8	σ*	305.10	0.32	0.283
	N4	LP	C8-H8	σ*	631.04	0.15	0.284
	N4	LP	C8-C9	σ*	40.88	0.78	0.163

	N4	LP	C9-C10	σ*	22.42	0.81	0.122
	N4	LP	C10-C11	π*	27.77	0.39	0.098
	N4	LP	C13-C14	σ*	5.93	0.88	0.107
	N4	LP	C15-C16	σ*	38.06	0.69	0.148
	N4	LP	C16-H16B	σ*	1709.60	0.07	0.312
	N4	LP	C17-H17A	σ*	17.64	0.81	0.109
	N4	LP	C18-C19	π*	659.02	0.12	0.265
	N4	LP	C19-C20	σ*	185.98	0.52	0.282
	N4	LP	C20-H20	σ*	29.51	0.76	0.137
	N4	LP	C20-C21	σ*	342.64	0.35	0.315
	N4	LP	C22-C23	σ*	460.09	0.82	0.559
	N4	LP	C22-C23	π*	336.03	0.62	0.433

[a]The interaction energy threshold is 10 kcal/mol.
